# Tom20 senses iron-activated ROS signaling to promote melanoma cell pyroptosis 

**DOI:** 10.1038/s41422-018-0090-y

**Published:** 2018-10-04

**Authors:** Bo Zhou, Jia-yuan Zhang, Xian-shuo Liu, Hang-zi Chen, Yuan-li Ai, Kang Cheng, Ru-yue Sun, Dawang Zhou, Jiahuai Han, Qiao Wu

**Affiliations:** State Key Laboratory of Cellular Stress Biology, School of Life Sciences, Xiamen University, Xiamen, Fujian 361005 China

## Abstract

Iron has been shown to trigger oxidative stress by elevating reactive oxygen species (ROS) and to participate in different modes of cell death, such as ferroptosis, apoptosis and necroptosis. However, whether iron-elevated ROS is also linked to pyroptosis has not been reported. Here, we demonstrate that iron-activated ROS can induce pyroptosis via a Tom20-Bax-caspase-GSDME pathway. In melanoma cells, iron enhanced ROS signaling initiated by CCCP, causing the oxidation and oligomerization of the mitochondrial outer membrane protein Tom20. Bax is recruited to mitochondria by oxidized Tom20, which facilitates cytochrome c release to cytosol to activate caspase-3, eventually triggering pyroptotic death by inducing GSDME cleavage. Therefore, ROS acts as a causative factor and Tom20 senses ROS signaling for iron-driven pyroptotic death of melanoma cells. Since iron activates ROS for GSDME-dependent pyroptosis induction and melanoma cells specifically express a high level of GSDME, iron may be a potential candidate for melanoma therapy. Based on the functional mechanism of iron shown above, we further demonstrate that iron supplementation at a dosage used in iron-deficient patients is sufficient to maximize the anti-tumor effect of clinical ROS-inducing drugs to inhibit xenograft tumor growth and metastasis of melanoma cells through GSDME-dependent pyroptosis. Moreover, no obvious side effects are observed in the normal tissues and organs of mice during the combined treatment of clinical drugs and iron. This study not only identifies iron as a sensitizer amplifying ROS signaling to drive pyroptosis, but also implicates a novel iron-based intervention strategy for melanoma therapy.

## Introduction

Reactive oxygen species (ROS) have been reported to be associated with cancer development and cancer cell death. At low to moderate levels, ROS promote tumor development by inducing DNA mutations and genomic instability or acting as signaling molecules that accelerate cancer cell proliferation, survival and metastasis.^1,2^ In contrast, excessive levels of ROS enhance cellular oxidative stress, which causes damage to DNA, proteins or lipids, leading to apoptotic or necroptotic cell death.^3,4^ For example, following treatment of apoptotic stimuli, the ROS-initiated oxidation of cardiolipin, which is a lipid located on the inner mitochondrial membrane, results in cytochrome c release, caspase activation and apoptotic cell death.^5^ Receptor-interacting protein kinase 3 (RIP3)-induced mitochondrial ROS generation leads to necroptosis in response to TNF-α stimulation.^6,7^ Therefore, boosting ROS in cancer cells by chemotherapeutic drugs has been applied in clinical cancer therapy.^2^

There are numerous ROS sources in cells, including iron-dependent ROS activation. First, iron is an essential component of several ROS-producing enzymes, such as NADPH oxidases (NOXs), lipoxygenases (LOXs), cytochrome P450 (CYP) enzymes and the mitochondrial electron transport chain subunits.^4^ Second, labile iron pools in cells directly catalyze ROS generation via the Fenton reaction.^4^ In most cells, excessive intracellular iron is stored in ferritin, where iron is safely sequestrated from being involved in ROS generation reactions.^8^ Ferritin comprises two subunits, the ferritin heavy chain (FTH) and ferritin light chain (FTL). The disruption of ferritin results in the elevation of ROS and cell death in an iron-dependent manner.^9,10^ Due to the important role of iron in the elevation of oxidative stress, targeting iron has emerged as a potential cancer therapy.^4^ However, the mechanism by which iron-induced ROS promote cell death remains ambiguous.

Apoptosis, necroptosis and ferroptosis have been shown to be associated with iron-triggered cell death via the ROS pathway,^11^ suggesting that iron likely plays a role in ROS signaling. Here, we further demonstrate that iron induces another type of cancer cell death, pyroptosis. Pyroptosis is a form of lytic programmed cell death initiated by inflammasomes, which activate caspase-1 or caspase-11/4/5 to cleave gasdermin D (GSDMD). The N-terminal pore-forming domain (PFD) of GSDMD oligomerizes to form nonselective pores in the membrane that drive cell swelling and membrane rupture.^12–15^ Recently, GSDME (original name: deafness autosomal dominant 5, DFNA5^16^) was also reported to be involved in pyroptosis induction. Following treatment with certain apoptotic stimuli, activated caspase-3 cleaves GSDME to release its PFD for pore formation, consequently triggering secondary necrosis after apoptosis or pyroptosis.^17,18^ Despite the well-known anti-infection effect of pyroptosis in immune cells, whether the induction of pyroptosis could be adopted in cancer therapy remains unclear.

Melanoma is among the most aggressive human cancers.^19^ Due to genetic or epigenetic alterations, melanoma cells are resistant to apoptotic induction.^20,21^ Therefore, developing new strategies for melanoma therapy is important. We previously reported that the induction of autophagic cell death by the small-molecule compound THPN could be an option for melanoma treatment.^22,23^ Here, we further combined iron with ROS-inducing drugs that can boost the cellular ROS level to activate the Tom20-Bax-caspase-GSDME signaling pathway and ultimately induce the pyroptotic death of melanoma cells. This antineoplastic effect of iron was further verified in mouse models. Collectively, our study illuminates that iron-based pyroptosis induction may be a novel, promising therapeutic strategy for the clinical treatment of melanoma.

## Results

### Iron boosts ROS to induce pyroptosis in melanoma cells

Iron is an essential factor for body homeostasis and participates in the regulation of cell survival. It has been previously reported that iron storage protein ferritin contributes to the melanoma progression by modulating cell growth and sensitivity to oxidative stress.^24^ Based on this finding, ferritin light chain protein (FTL) or ferritin heavy chain 1 (FTH1), two components of ferritin, was separately knocked down in melanoma A375 cells (Supplementary information, Fig. S1a), and several reagents that activate oxidative stress, including carbonyl cyanide m-chlorophenyl hydrazone (CCCP),^25^ NaAsO_2_,^26^ H_2_O_2_, antimycin A,^27^ and oligomycin,^28^ were used to evaluate the role of iron metabolism in cell death. Although these reagents hardly influenced A375 cell survival, they induced cell death in the FTL- or FTH1-knockdown A375 cells (Supplementary information, Fig. S1b). Among these compounds, we noted that CCCP treatment was not only most effective in cell death induction, but also obviously induced the characteristic morphological features of pyroptosis, as evidenced by cell swelling, large bubbles blowing from the plasma in the FTL- or FTH1-knockdown A375 cells (Supplementary information, Fig. S1c, pyroptotic cells were indicated by red arrows). Therefore, it is likely that CCCP may specifically induce melanoma cell death through pyroptosis when endogenous ferritin is disrupted.

**Fig. 1:**
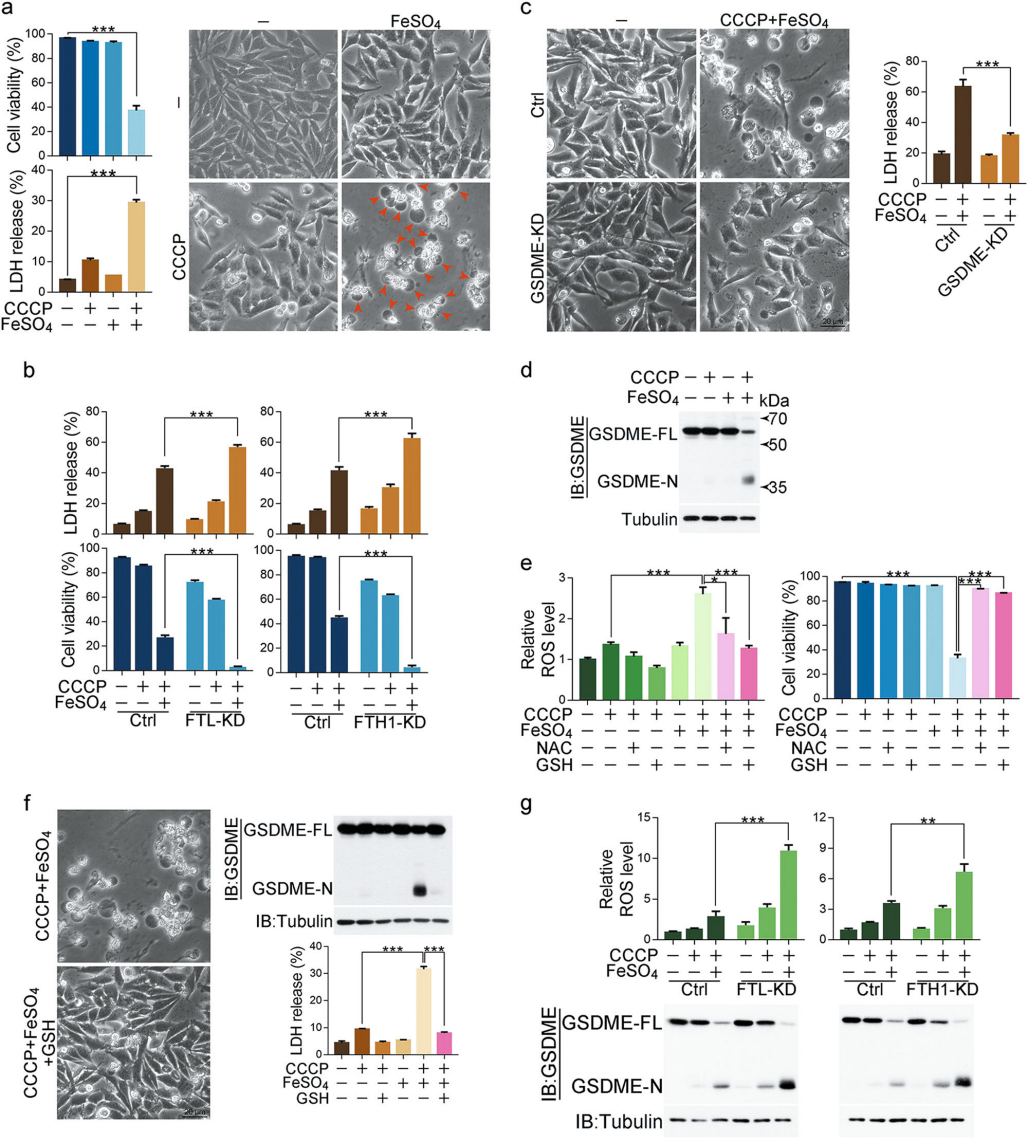
Iron activates ROS to promote the pyroptotic death of melanoma cells. Melanoma A375 cells were pretreated with or without different inhibitors, including NAC (5 mM) or GSH (1 mM), for 2 h, followed by CCCP (20 μM), FeSO_4_ (100 μM), or CCCP/FeSO_4_ treatment for 6 h to detect the ROS level or 24 h to assess the pyroptotic features (including morphology, GSDME cleavage, and LDH release) and cell viability, unless specifically defined. **a** The addition of FeSO_4_ to CCCP induced cell death, LDH release, and pyroptosis (pyroptotic cells are indicated by red arrows). **b** Knockdown of FTL or FTH1 enhanced LDH release and cell death in response to CCCP/FeSO_4_ stimulation. KD: knockdown. **c** Knockdown of GSDME diminished FeSO_4_-induced pyroptosis and LDH release in the presence of CCCP. **d** Cleavage of GSDME was observed in response to CCCP/FeSO_4_ stimulation. **e** The effects of NAC or GSH on CCCP/FeSO_4_-induced ROS elevation and cell death. **f** GSH blocked CCCP/FeSO_4_-induced pyroptosis, GSDME cleavage and LDH release. **g** Knockdown of FTL or FTH1 enhanced CCCP/FeSO_4_-induced LDH release and GSDME cleavage. Tubulin was used to determine the amount of loading proteins. All data are presented as the mean ± SEM of three independent experiments. **P *<* 0.01, ***P < 0.001

To confirm the requirement of endogenous iron for pyroptosis in the FTH1- or FTL-knockdown A375 cells, cells were pretreated with deferoxamine (DFO, an iron chelating agent) before CCCP stimulation. Under this circumstance, CCCP-induced morphology of pyroptosis and release of lactate dehydrogenase (LDH, an indication of pyroptotic cell cytotoxicity) were remarkably attenuated (Supplementary information, Fig. S1d), which illustrates the involvement of endogenous iron in CCCP-induced pyroptosis. We further determined whether this CCCP-induced pyroptosis is directly associated with iron overload. A375 cells were treated with CCCP plus exogenous iron (FeSO_4_ in the current study) without knocking down FTH1 or FTL. Co-treatment of CCCP/iron showed more pronounced inhibition in cell survival accompanied with significant pyroptosis and LDH release, as compared to CCCP treatment alone (Fig. 1a; Supplementary information, Movie S1). Notably, the effects of exogenous iron on LDH release and cell death were more evident in the FTH1- or FTL-knockdown A375 cells (Fig. 1b). Therefore, blocking ferritin would facilitate iron to exert its function in driving melanoma cells to pyroptotic death in the presence of CCCP. We thus chose CCCP/iron for the further study.

Because GSDMD and GSDME have been reported to function as pyroptotic executioners,^12–14,17,18^ we investigated whether they are involved in CCCP/iron-induced pyroptosis. Knockdown of GSDME (Supplementary information, Fig. S1e) obviously inhibited the CCCP/iron-induced pyroptosis and LDH release (Fig. 1c), whereas knockdown of GSDMD failed to exert this inhibitory effect (Supplementary information, Fig. S1f). Moreover, CCCP/iron, but not CCCP alone, efficiently induced the cleavage of GSDME (Fig. 1d), another characteristic pyroptotic marker. This GSDME cleavage was also observed upon CCCP stimulation in the FTH1- or FTL-knockdown A375 cells, which could be abolished by DFO pretreatment (Supplementary information, Fig. S1g). The cleavage of GSDME occurred at _267_DMPD_270_ sites, because transfection of mutant GSDME^D267A^ or GSDME^D270A^ effectively abolished GSDME cleavage even in the presence of CCCP/iron (Supplementary information, Fig. S1h, left), which is consistent with previous reports.^17,18^ Transfection of GSDME N-terminus (a.a. 1–270), but not C-terminus (a.a. 271–497), was sufficient to induce pyroptosis in 293 T cells (Supplementary information, Fig. S1h, right). These results suggest the presence of GSDME-dependent pyroptosis in the CCCP/iron case.

Inorganic iron is basically elemental iron that often presents itself as either Fe^2+^ or Fe^3+^. To test whether Fe^3+^ also potentiates CCCP-induced pyroptosis, A375 cells were treated with Fe_2_(SO_4_)_3_ for 24 h in the presence of CCCP. The results showed that Fe_2_(SO_4_)_3_ also induced pyroptosis, GSDME cleavage, and LDH release as effectively as FeSO_4_ (Supplementary information, Fig. S1i). Since iron ion can be interconverted easily between ferrous (Fe^2+^) and ferric (Fe^3+^) forms in cells,^29^ it is likely that iron ion in either Fe^2+^ or Fe^3+^ form is efficient in induction of pyroptosis. Physiologically, extracellular Fe^2+^ is directly transported into cells by DMT1 (divalent metal transporter), while Fe^3+^ is assembled in its chaperon transferrin and then imported into cells by TfR1 and TfR2 (transferrin receptors).^29^ To further determine whether Fe^2+^ and Fe^3+^ need to be transported into cells to mediate cell death, DMT1, TfR1 or TfR2 was separately knocked down in A375 cells (Supplementary information, Fig. S1j) before Fe^2+^ or Fe^3+^ stimulation. Under these circumstances, CCCP/FeSO_4_- or CCCP/Fe_2_(SO_4_)_3_-induced pyroptosis, GSDME cleavage, and LDH release were substantially attenuated (Supplementary information, Fig. S1k, l). These results further support the intrinsic mechanism of iron-induced pyroptotic cell death.

We also wondered whether this CCCP/iron-induced pyroptosis is a general phenomenon. Upon CCCP/iron stimulation, pyroptosis was observed in four other melanoma cell lines that expressed GSDME, regardless of the BRAF mutation status (Supplementary information, Fig. S1m). However, this CCCP/iron-induced pyroptosis seemed specific to melanoma cells since other types of cell lines, Huh7 hepatoma cells that did not express GSDME was resistant to CCCP/iron stimulation, whereas A549 lung cancer cells and MDA-MB-231 breast cancer cells that expressed a comparable level of GSDME with A375 melanoma cells, were not as sensitive as A375 cells to CCCP/iron-induced pyroptosis (Supplementary information, Fig. S1n).

Numerous reports have demonstrated that iron in cells is a ROS enhancer.^4,30,31^ We also found that addition of iron to CCCP treatment indeed induced A375 cell death by boosting ROS level. Although CCCP or iron treatment alone only slightly elevated cellular ROS, treatment of CCCP and iron synergistically enhanced ROS level, leading to obvious cell death (Fig. 1e). However, NAC (N-acetyl-L-cysteine) and GSH (glutathione), which are inhibitors of ROS, markedly attenuated the CCCP/iron-induced ROS elevation, thereby rescuing cell death (Fig. 1e). Expectedly, GSH reversed the phenotype of the CCCP/iron-treated A375 cells from pyroptosis to normal state and abolished GSDME cleavage and LDH release (Fig. 1f). Upon knockdown of FTL or FTH1, iron further promoted ROS increase and GSDME cleavage (Fig. 1g). Therefore, it is iron that dramatically induces pyroptosis in a ROS-dependent manner in melanoma cells, and ROS thus acts as a causative signal for iron-driven cell death.

To test whether this ROS- and iron-dependent pyroptosis induction is a general mechanism, A375 cells were treated with several reagents including actinomycin-D, doxorubicin, or etoposide that are reported to induce pyroptosis.^18^ Although these reagents could effectively elevate cellular ROS level (Supplementary information, Fig. S1o, top), pretreatment of GSH could not alleviate pyroptotic cell death (Supplementary information, Fig. S1o, bottom). Similarly, the pyroptosis induced by these reagents was not affected by DFO pretreatment (Supplementary information, Fig. S1o, bottom). Hence, the mechanisms of pyroptosis induced by these reagents are different from that of CCCP/iron-stimulated pyroptosis. We also excluded the possibility of iron-induced ferroptosis, another type of iron-dependent cell death, by using ferrostatin-1 and a series of MEK inhibitors (including U0126, AZD6244, AZD8330 and PD98059) that have been reported to block the induction of ferroptosis.^30^ The results revealed that separate preincubation with these inhibitors did not impair the CCCP/iron-induced LDH release (Supplementary information, Fig. S1p). In addition, CCCP/iron treatment for 24 h could barely induce the apoptosis of melanoma cells, as no obvious apoptotic features were detected, including DNA laddering (Supplementary information, Fig. S1q), annexin V (AnnV) single positive cells (Supplementary information, Fig. S1r), and nuclear fragmentation (Supplementary information, Fig. S1s). In contrast, these phenomena were clearly observed upon stimulation of staurosporine (STS, a well-known apoptosis inducer). Taken together, a series of data implicate that CCCP/iron treatment efficiently induces melanoma cell death via pyroptosis but not ferroptosis or apoptosis. However, we detected weaker cleavage of PARP upon CCCP/iron stimulation (Supplementary information, Fig. S1t), which may be due to the activation of caspase-3. This result not only confirmed that pyroptosis and apoptosis share many common upstream regulators, but also implied that inhibition of pyroptosis by reducing GSDME expression may shift the cell death from pyroptosis to apoptosis. Indeed, although GSDME-knockdown A375 cells or Mel-11 and ME4405 cells (non-expression of GSDME) were resistant to CCCP/iron-induced cell death at 24 h post treatment, apoptotic cells appeared after 36 h of treatment (Supplementary information, Fig. S1u).

### Induction of pyroptosis by iron is linked to the mitochondrial pathway

**Fig. 2:**
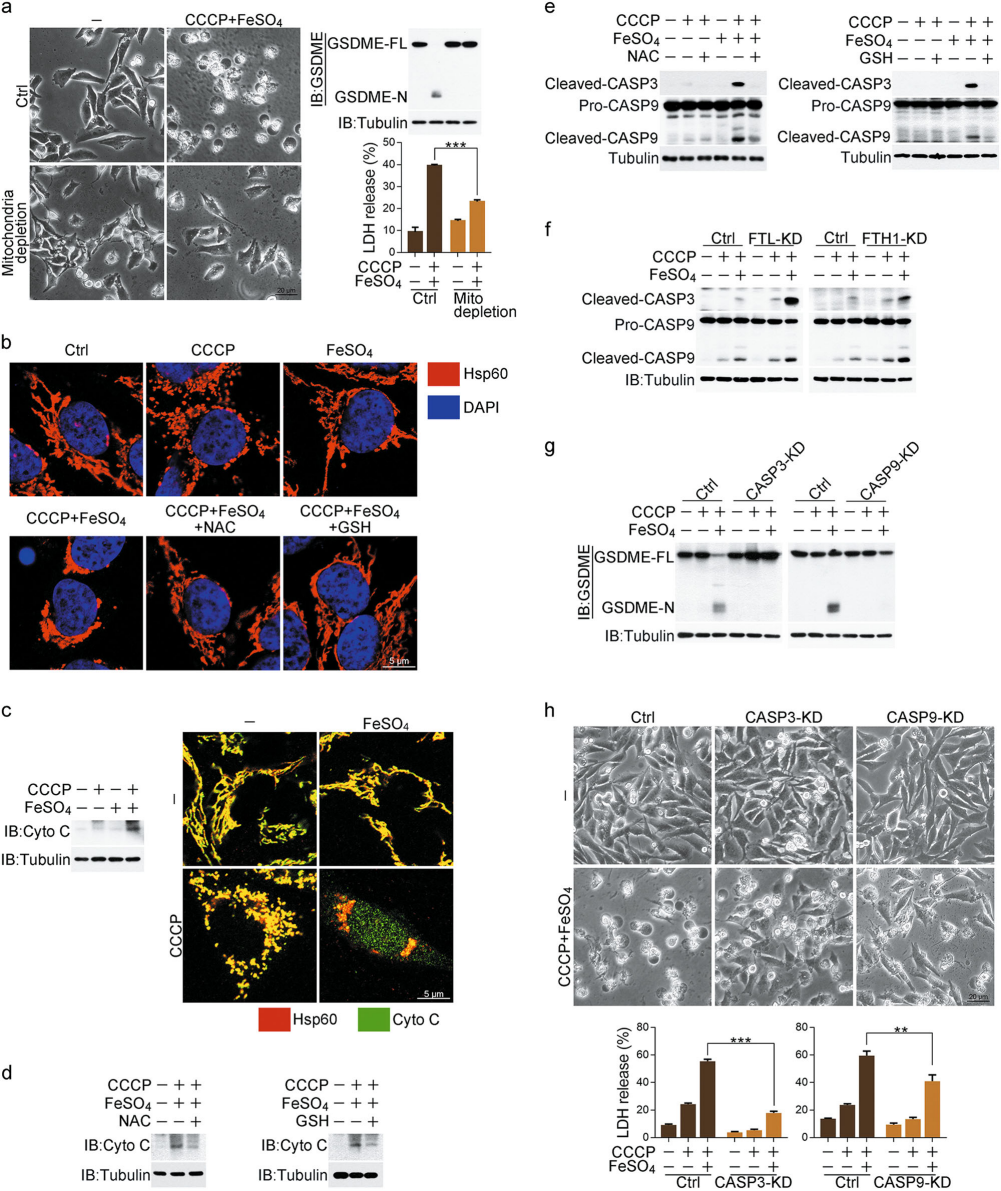
Mitochondrial pathway with activation of caspase-3 is involved in pyroptosis induced by iron. Melanoma A375 cells were pretreated with or without different inhibitors, including NAC (5 mM) or GSH (1 mM) for 2 h, followed by CCCP (20 μM), FeSO_4_ (100 μM), or CCCP/FeSO_4_ treatment for 6 h to detect cytochrome c release and caspase-3 or -9 cleavage or 24 h to assess the pyroptotic features (including morphology, GSDME cleavage, and LDH release), unless specifically defined. **a** Mitochondrial depletion blocked CCCP/FeSO_4_-induced pyroptosis, GSDME cleavage and LDH release. Mito: mitochondria. **b** CCCP/FeSO_4_ induced mitochondrial accumulation, but NAC and GSH attenuated this accumulation. **c** CCCP/FeSO_4_ induced cytochrome c release from mitochondria to cytosol as detected in the cytosol fraction (left) or by confocal microscopy (right). Cyto C: cytochrome c. **d**, **e** NAC or GSH abolished CCCP/FeSO_4_-induced cytochrome c release (**d**) and cleavage of caspase-3 and -9 (**e**). CASP: caspase. **f** Knockdown of FTL or FTH1 enhanced the CCCP/FeSO_4_-induced cleavage of caspase-3 and -9. **g**, **h** Knockdown of either caspase-3 or -9 abolished the CCCP/FeSO_4_-induced GSDME cleavage (**g**), pyroptosis and LDH release (**h**). Tubulin was used to determine the amount of loading proteins. All data are presented as the mean ± SEM of three independent experiments. **P *<* 0.01, ***P *<* 0.001

It has been reported that mitochondrial pathway is involved in GSDME-mediated pyroptosis.^17^ We then tested whether mitochondria are required for the CCCP/iron-induced pyroptosis. CCCP is a mitochondrial uncoupler that can induce mitochondrial depletion via the mitophagy pathway. However, in A375 cells, CCCP treatment failed to trigger mitophagy due to the absence of Parkin expression (Supplementary information, Fig. S2a), in line with the previously report.^32^ To eliminate mitochondria, A375 cells were transfected with Parkin (Supplementary information, Fig. S2b, left) and subjected to a short-term CCCP treatment for widespread mitophagy induction.^33^ In these cells, mitophagy, indicated by the co-localization of mitochondria with LC3, was clearly observed (Supplementary information, Fig. S2a, right), and mitochondrial proteins or mitochondrial DNA (mtDNA) could be hardly detected (Supplementary information, Fig. S2b, middle and right), which indicates the effective mitochondrial depletion. Under this circumstance, the CCCP/iron-induced pyroptotic features, such as cell swelling, LDH release and GSDME cleavage, could barely be detected (Fig. 2a). Thus, CCCP/iron-induced pyroptosis is likely associated with the mitochondrial pathway.

CCCP treatment alone could cause the normal, tubular mitochondria network to fragment into short rods or spheres in A375 cells (Fig. 2b).^34^ Although iron treatment alone did not influence the morphology of mitochondria, co-treatment with CCCP induced the aggregation of mitochondrial fragments (Fig. 2b). This mitochondrial aggregation could be abolished by NAC or GSH, suggesting that ROS are required for this process. Mitochondrial aggregation has been reported to contribute to cytochrome c release from mitochondria to cytosol.^35^ Consistently, cytochrome c release to cytosol in response to CCCP/iron stimulation was detected in an ROS-dependent manner (Fig. 2c, d). Consequently, CCCP/iron effectively induced the cleavage of caspase-9 and -3, which was also blocked by NAC or GSH incubation (Fig. 2e). After ferritin was disrupted by FTL or FTH1 knockdown in A375 cells, the cleavage of caspase-9 and -3 was further enhanced by the CCCP/iron treatment (Fig. 2f). Although caspase-3, but not caspase-9, cleaved GSDME in vitro (Supplementary information, Fig. S2c), knockdown of either caspase-3 or -9 (Supplementary information, Fig. S2d) resulted in the blockade of GSDME cleavage, LDH release, and the pyroptotic phenotype (Fig. 2g, h). This result is consistent with the hypothesis that caspase-9 cleaves and activates caspase-3, which then cleaves GSDME.^17,18^ Combined with the observation that inhibition of the caspases by Z-VAD had no effect on the CCCP/iron-induced ROS elevation and cytochrome c release (Supplementary information, Fig. S2e) but had obvious effect on cell swelling, LDH release, GSDME cleavage and cell death (Supplementary information, Fig. S2f), we conclude that iron-amplified ROS signaling may function as an upstream factor promoting mitochondrial aggregation and subsequent cytochrome c release, thereby executing pyroptosis via the cleavage of GSDME by caspase-3.

### Tom20 senses and transmits ROS signaling to mitochondria

**Fig. 3:**
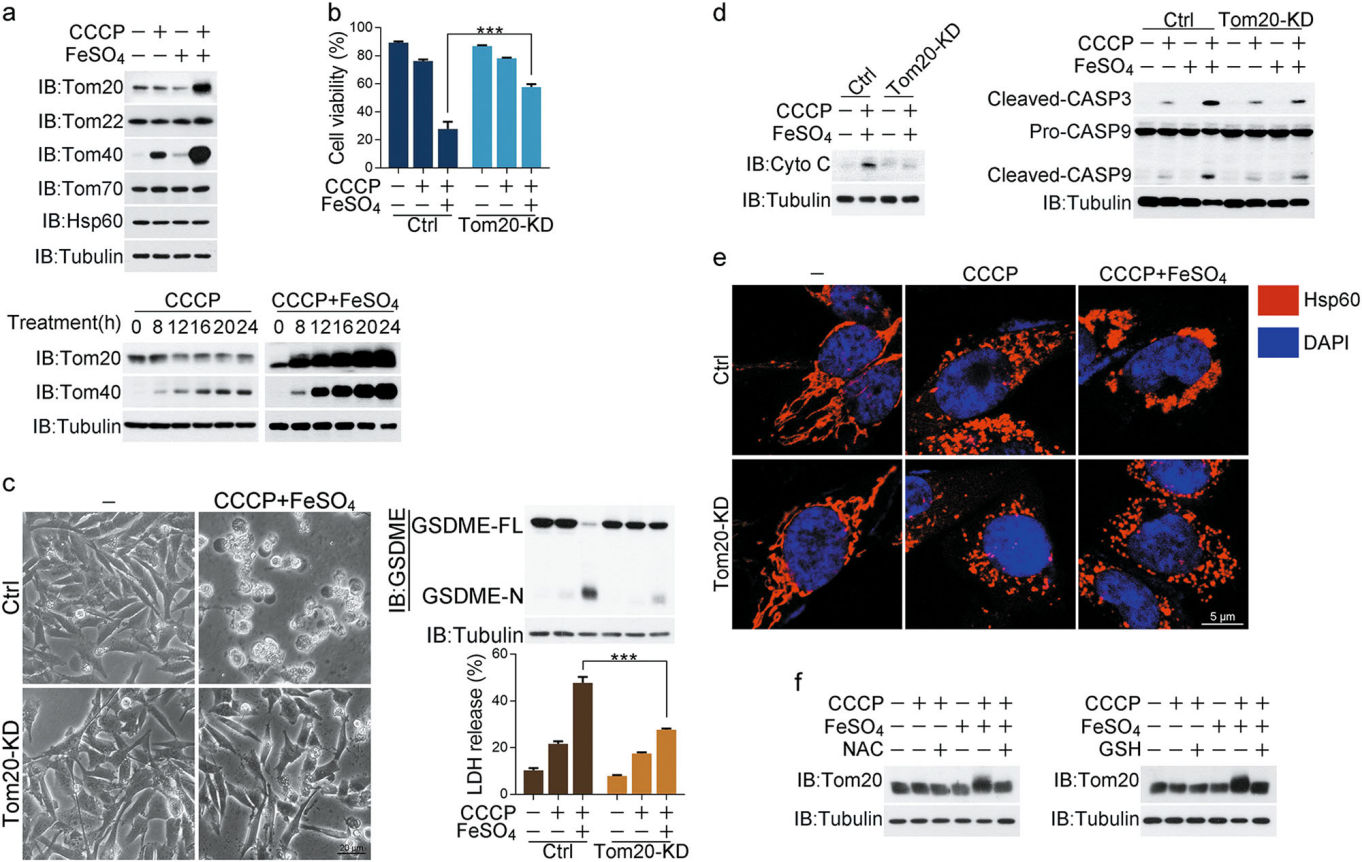
Iron-induced Tom20 accumulation promotes pyroptosis. Melanoma A375 cells were pretreated with or without different inhibitors, including NAC (5 mM) or GSH (1 mM) for 2 h, followed by CCCP (20 μM), FeSO_4_ (100 μM), or CCCP/FeSO_4_ treatment for 6 h to detect cytochrome c release and caspase-3 or -9 cleavage or 24 h to assess the pyroptotic features (including morphology, GSDME cleavage, and LDH release) and cell death, unless specifically defined. **a** Top, addition of FeSO_4_ to CCCP induced Tom20 and Tom40 accumulation. Bottom, the effect of CCCP/FeSO_4_ on the expression levels of Tom20 and Tom40. Cells were treated with CCCP or CCCP/FeSO_4_ for the indicated times. **b** Knockdown of Tom20 rescued the cell viability in response to CCCP/FeSO_4_ stimulation. **c**–**e** Knockdown of Tom20 reversed the CCCP/FeSO_4_-induced cell morphology from pyroptosis to normal state, reduced LDH release and blocked GSDME cleavage (**c**), abolished cytochrome c release (detected in the cytosol fraction) and cleavage of caspase-3 and -9 (**d**), and blocked mitochondria aggregation (**e**). Hsp60 was used as a mitochondrial indicator, and DAPI was used to display the nuclei. **f** NAC and GSH attenuated the CCCP/FeSO_4_-induced Tom20 accumulation. Tubulin was used to determine the amount of loading proteins. Hsp60 was used to determine the amount of mitochondrial proteins. All data are presented as the mean ± SEM of three independent experiments. ***P *<* 0.001

If iron drives cytochrome c release from mitochondria to cytosol, the proteins located in the outer mitochondrial membrane are likely regulated by iron; among these proteins, the Tom complex attracted our attention. The protein levels of Tom20 and Tom40, but not Tom70 and Tom22, were dramatically increased by CCCP/iron in a time-dependent manner (Fig. 3a). The CCCP treatment alone was sufficient to induce Tom40 accumulation, which is consistent with a previous report.^36^ However, Tom20 accumulation could be detected only following co-treatment of CCCP/iron (Fig. 3a). We further investigated whether this accumulation of Tom20 or Tom40 is involved in CCCP/iron-induced pyroptosis. Knockdown of Tom20 (Supplementary information, Fig. S3a) obviously increased the cell viability in A375 cells upon CCCP/iron stimulation (Fig. 3b), which was accompanied by inhibition of GSDME cleavage, LDH release and cell swelling (Fig. 3c). In contrast, knockdown of Tom40 (Supplementary information, Fig. S3a) had no effect on both cell death and the pyroptotic features (Supplementary information, Fig. S3b, c). Therefore, Tom20 is involved in iron-induced pyroptosis.

The relationship of the up- and downstream regulators in iron-induced pyroptosis was further analyzed in Tom20- or Tom40-knockdown cells. CCCP/iron-induced cytochrome c release and caspase-3/9 cleavage were clearly impaired by Tom20, but not Tom40, knockdown (Fig. 3d; Supplementary information, Fig. S3d), whereas inhibition of the caspases by Z-VAD could not influence the CCCP/iron-induced Tom20 accumulation (Supplementary information, Fig. S3e). Although the CCCP/iron treatment did not influence the mitochondrial location of Tom20 (Supplementary information, Fig. S3f), knockdown of Tom20 evidently weakened the CCCP/iron-induced mitochondrial aggregation (Fig. 3e). Altogether, Tom20 is likely an upstream regulator of mitochondrial aggregation and cytochrome c release in response to iron stimulation. Furthermore, knockdown of either Tom20 or Tom40 could not influence the CCCP/iron-induced ROS elevation (Supplementary information, Fig. S3g). In contrast, the CCCP/iron-induced Tom20 accumulation was abolished by the ROS scavengers NAC and GSH (Fig. 3f). Collectively, Tom20, which is a novel downstream factor of ROS, likely senses iron-boosted ROS signaling and transmits this signal to mitochondria to induce cytochrome c release, ultimately inducing pyroptosis via cleavage of GSDME by caspase-3.

### ROS-induced oxidation of Tom20 promotes pyroptosis

**Fig. 4:**
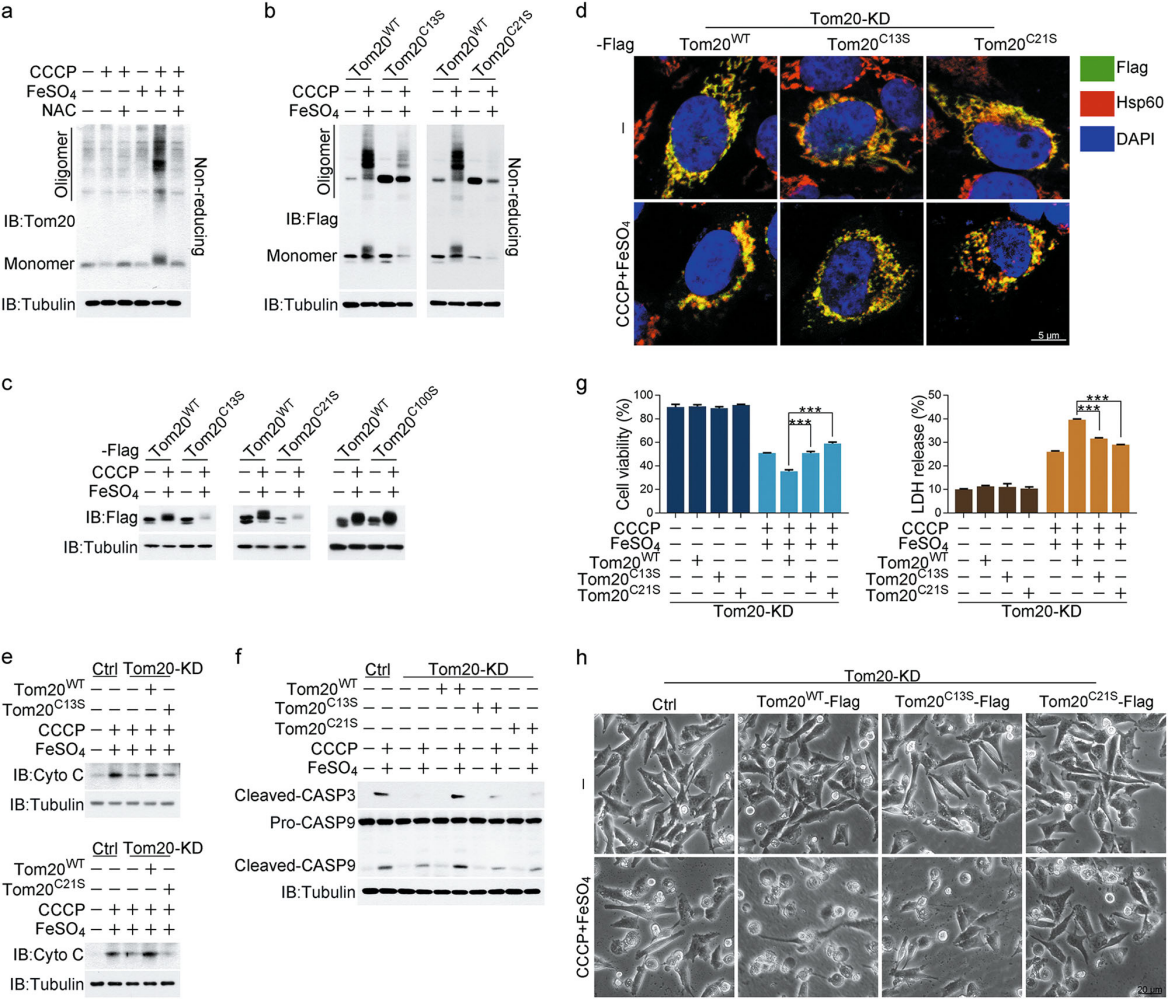
ROS induces Tom20 oxidation in response to iron stimulation. Melanoma A375 cells were pretreated with or without different inhibitors, including NAC (5 mM) for 2 h, followed by CCCP (20 μM), FeSO_4_ (100 μM), or CCCP/FeSO_4_ treatment for 6 h to detect cytochrome c release and caspase-3 or -9 cleavage or 24 h to assess the pyroptotic features (including morphology, GSDME cleavage, and LDH release) and cell death, unless specifically defined. To detect the effects of the Tom20 point mutants Tom20^C13S^, Tom20^C21S^ and Tom20^C100S^, Tom20 was knocked down in A375 cells, and Tom20^WT^ or its point mutants Tom20^C13S^, Tom20^C21S^ and Tom20^C100S^ were separately transfected into cells. **a** FeSO_4_ induced Tom20 oxidation, which was attenuated by NAC even in the presence of CCCP. Western blotting was performed under reducing and non-reducing conditions to determine the Tom20 oxidation status. **b** Mutation of either Cys13 or Cys21 in Tom20 abolished the CCCP/FeSO_4_-induced Tom20 oxidation. **c** CCCP/FeSO_4_ could not induce Tom20^C13S^ and Tom20^C21S^ accumulation. **d** Mutation of either Cys13 or Cys21 in Tom20 blocked mitochondrial aggregation as shown by confocal microscopy. Hsp60 represents the mitochondria, and DAPI was used to display the nuclei. **e-h** Mutation of either Cys13 or Cys21 in Tom20 abrogated the CCCP/FeSO_4_-induced cytochrome c release as detected in the cytosol fraction (**e**), cleavage of caspase-3 and -9 (**f**), LDH release and cell death (**g**), and reversed the cell morphology from pyroptosis to normal state (**h**). Tubulin was used to determine the amount of loading proteins. Hsp60 was used to determine the amount of mitochondrial proteins. All data are presented as the mean ± SEM of three independent experiments. ***P *<* 0.001

ROS often regulate protein functions via oxidation of cysteines, which results in the formation of intra- or intermolecular disulfide bonds.^37^ Thus, we analyzed the migration of Tom20 using SDS-PAGE under nonreducing conditions. In response to CCCP/iron stimulation, Tom20, but not Tom40, was clearly detected in a high-molecular-weight complex, which was abolished by NAC treatment (Fig. 4a; Supplementary information, Fig. S4a). This demonstrated that iron-induced ROS causes Tom20 oligomerization likely via oxidation of Tom20 to form intermolecular disulfide bonds. Three Cys residues (Cys13, Cys21, and Cys100) in the Tom20 molecule are conserved in several species (Supplementary information, Fig. S4b). In A375 cells, transfection of Tom20^C13S^ or Tom20^C21S^, but not Tom20^C100S^, significantly abolished CCCP/iron-induced Tom20 oligomerization (Fig. 4b; Supplementary information, Fig. S4c), suggesting that both Cys13 and Cys21 are required for the intermolecular disulfide bond formation in Tom20 molecules. Consequently, the CCCP/iron-induced Tom20 accumulation could not be detected if Cys13 or Cys21 was mutated (Fig. 4c). Thus, the iron-enhanced ROS caused Tom20 oxidation at Cys13 and Cys21, which is associated with the oligomerization and accumulation of Tom20.

To further verify the role of oxidation in Tom20-associated functions, siRNA-resistant forms of Tom20^WT^, Tom20^C13S^ and Tom20^C21S^ were separately introduced into the Tom20-knockdown A375 cells (Supplementary information, Fig. S4d). CCCP/iron-induced mitochondrial aggregation, cytochrome c release and cleavage of caspase-3/9 could only be detected in Tom20^WT^-, but not Tom20^C13S^- or Tom20^C21S^-expressing cells (Fig. 4d–f). Consequently, the pyroptotic features, such as LDH release, cell swelling and cell death, were diminished by the C13S or C21S mutation of Tom20 (Fig. 4g, h). Clearly, Tom20 oxidation is a prerequisite for iron-induced pyroptosis.

### Tom20 oxidation facilitates Bax recruitment to mitochondria

**Fig. 5:**
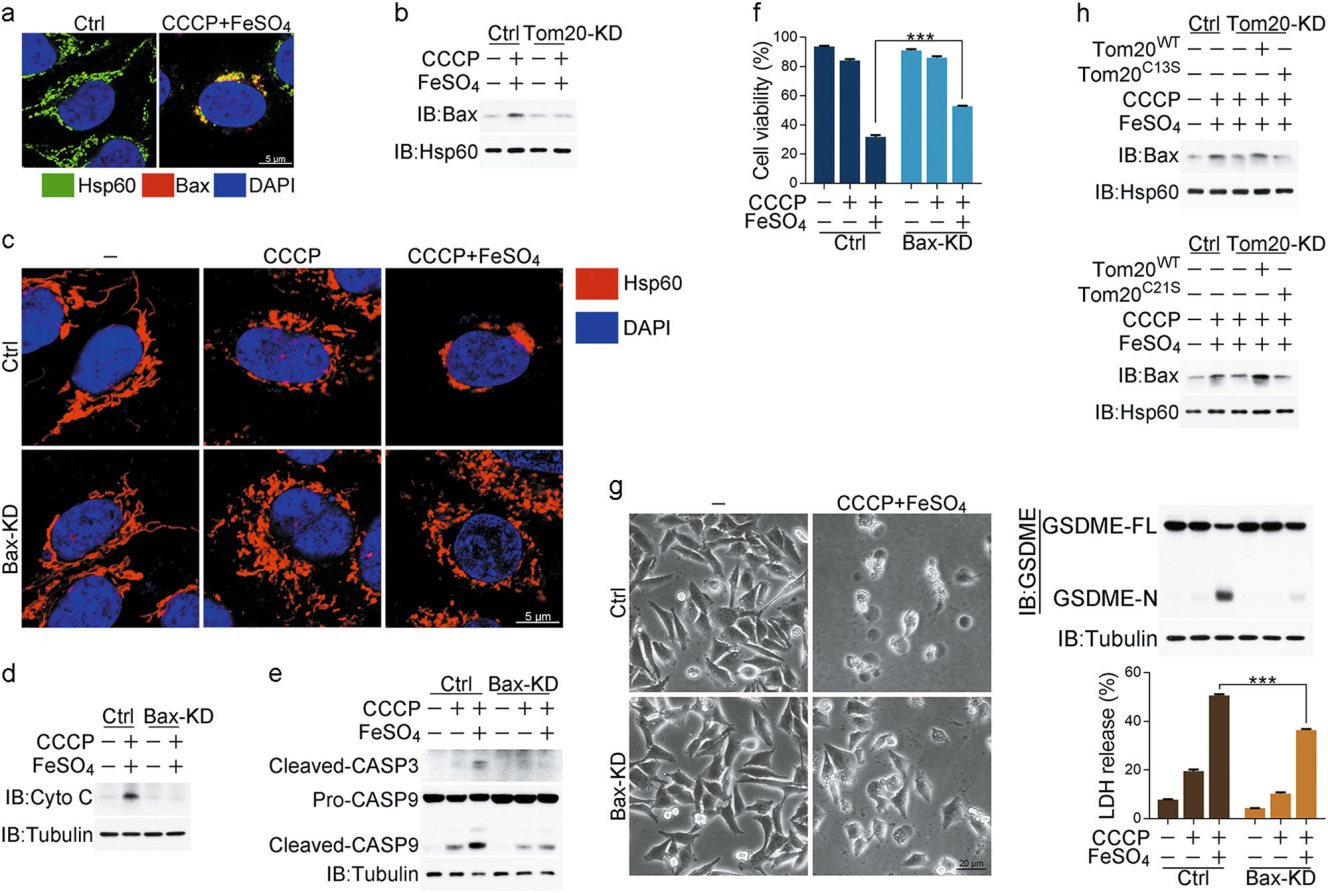
Tom20-induced translocation of Bax to mitochondria contributes to pyroptosis. Melanoma A375 cells were treated with CCCP (20 μM), FeSO_4_ (100 μM), or CCCP/FeSO_4_ for 6 h to detect the translocation of Bax to mitochondria, cytochrome c release and caspase-3 or -9 cleavage or 24 h to assess the pyroptotic features (including morphology, GSDME cleavage, and LDH release) and cell death, unless specifically defined. To detect the effects of the Tom20 point mutants Tom20^C13S^ and Tom20^C21S^, Tom20 was knocked down in A375 cells, and Tom20^WT^ or its point mutants Tom20^C13S^ and Tom20^C21S^ were separately transfected into cells. **a** Bax translocated to mitochondria upon CCCP/FeSO_4_ stimulation as shown by confocal microscopy. **b** Knockdown of Tom20 blocked the translocation of Bax to mitochondria upon CCCP/FeSO_4_ treatment. The mitochondrial fraction in cells was prepared. **c** Knockdown of Bax blocked the CCCP/FeSO_4_-induced mitochondrial aggregation. **d-g** After knocking down Bax, the CCCP/FeSO_4_-induced cytochrome c release detected in the cytosol fraction was diminished (**d**), the cleavage of caspase-3 and -9 was attenuated (**e**), the cell viability was rescued (**f**), the cell morphologies were reversed from pyroptosis to normal state, and LDH release and GSDME cleavage were also abolished (**g**). **h** Effects of the mutants Tom20^C13S^ and Tom20^C21S^ on the translocation of Bax to mitochondria in response to CCCP/FeSO_4_ stimulation. Hsp60 was used to detect the mitochondria, and DAPI was used to display the nuclei by confocal microscopy. Tubulin was used to determine the amount of loading proteins. Hsp60 was used to determine the amount of mitochondrial proteins. All data are presented as the mean ± SEM of three independent experiments. ***P *<* 0.001

The Tom complex has been reported to be important for Bax mitochondrial localization to induce cytochrome c release.^38^ We thus investigated whether Bax is involved in iron-induced pyroptosis via Tom20 modulation. The CCCP/iron treatment clearly induced Bax translocation from cytoplasm to mitochondria (Fig. 5a, b), which was abolished by Tom20 knockdown (Fig. 5b), indicating the Tom20-dependent mitochondrial localization of Bax in response to CCCP/iron. Notably, knockdown of Bax (Supplementary information, Fig. S5a) not only abolished the CCCP/iron-induced mitochondrial aggregation (Fig. 5c), cytochrome c release to cytosol (Fig. 5d) and caspase-3/9 cleavage (Fig. 5e), but also suppressed melanoma cell death (Fig. 5f) and pyroptosis (Fig. 5g). Obviously, Bax is required for iron-induced pyroptosis likely via Tom20.

Following the treatment of CCCP/iron, the interaction between Tom20 and Bax was substantially enhanced (Supplementary information, Fig. S5b). This effect relied on Tom20 oxidation since if the oxidation sites were mutated, the interaction between Bax and Tom20^C13S^ or Tom20^C21S^ was no longer regulated by CCCP/iron (Supplementary information, Fig. S5c). Combined with the observation that CCCP/iron did not influence the Tom20 mitochondrial location (Supplementary information, Fig. S3f), we propose that oxidized Tom20 interacts with and recruits Bax to mitochondria. Indeed, Tom20^C13S^ and Tom20^C21S^ lost their abilities to recruit Bax to mitochondria (Fig. 5h). Altogether, the iron-induced oxidation of Tom20 recruits Bax to mitochondria, which promotes Bax-dependent cytochrome c release and results in the pyroptotic death of melanoma cells.

Finally, to test whether Tom20 oxidation is also involved in other mitochondria-mediated cell death pathway, such as the tBid/Bax-induced pyroptosis, A375 cells were treated with TNFα/CHX for 24 h, based on the report that TNFα plus cycloheximide (CHX) treatment induces a tBid/Bax-dependent cytochrome c release and caspase-9 activation,^39^ consequently leading to pyroptosis in GSDME-expressing cells.^18^ Expectedly, TNFα/CHX significantly induced Tom20 oxidation (Supplementary information, Fig. S5d), accompanied with GSDME cleavage and pyroptosis induction (Supplementary information, Fig. S5e). Therefore, Tom20 oxidation may also apply to other mitochondria-mediated pyroptotic cell death.

### Iron elevates ROS induced by different drugs to initiate pyroptosis

**Fig. 6:**
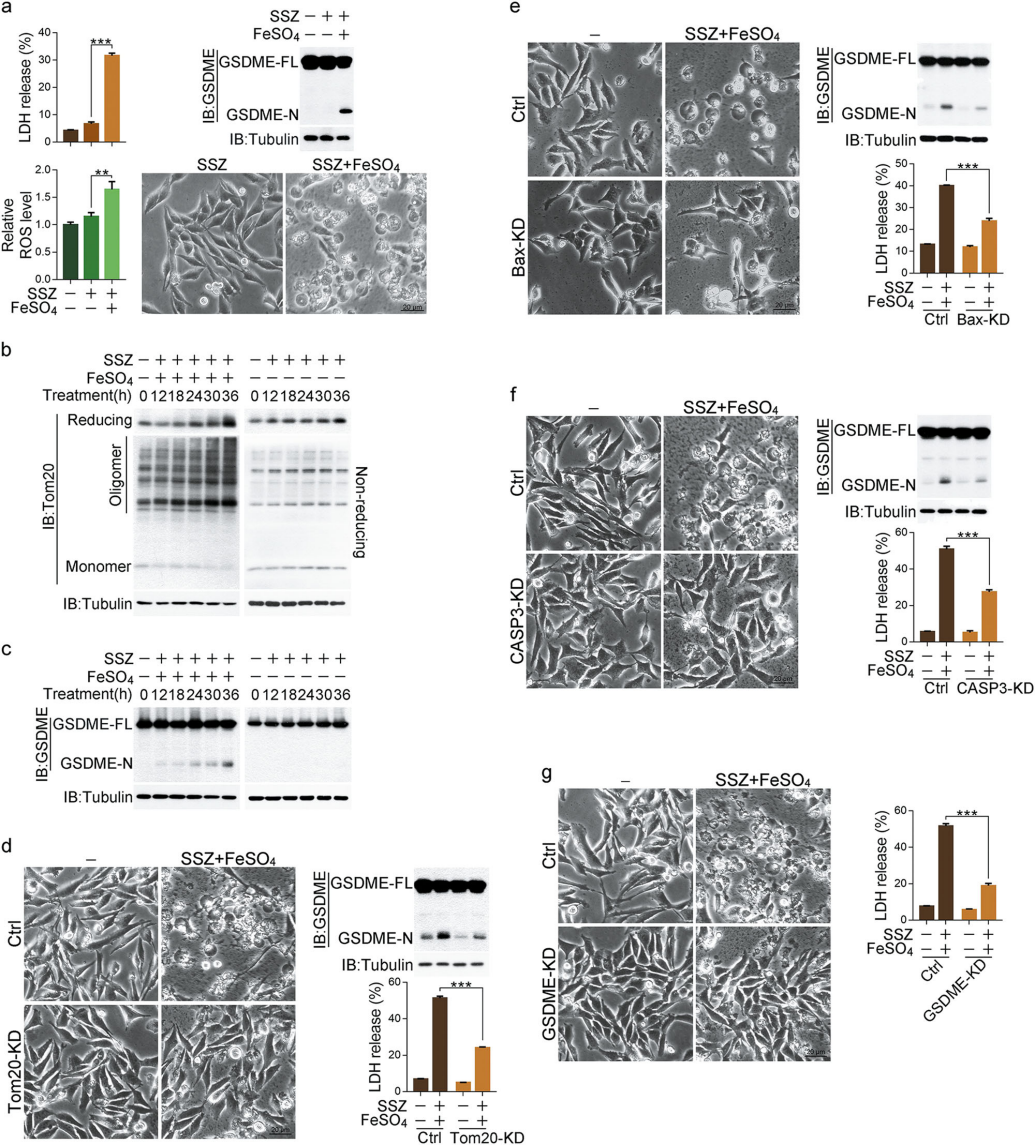
Iron acts as a sensitizer for different drugs and induces pyroptosis in melanoma cells. Melanoma A375 cells were treated with SSZ (sulfasalazine, 125 μM) with or without FeSO_4_ (100 μM) for 6 h to detect the ROS level or 24 h to assess the pyroptotic features (including morphology, GSDME cleavage, and LDH release), unless specifically defined. **a** FeSO_4_ acts as a sensitizer for ROS generation, GSDME cleavage, LDH release, and pyroptosis in the presence of SSZ. **b**, **c** Extensive treatment with SSZ/FeSO_4_ at the indicated times induced Tom20 oxidation and accumulation (**b**) and GSDME cleavage (**c**). **d**–**g** Separate knockdown of Tom20, Bax, caspase-3 or GSDME blocked SSZ/FeSO_4_-induced pyroptosis, GSDME cleavage, and LDH release as indicated. Tubulin was used to determine the amount of loading proteins. All data are presented as the mean ± SEM of three independent experiments. **P *<* 0.01, ***P *<* 0.001

Having established that iron amplifies ROS signaling to induce pyroptosis, we further investigated whether iron can sensitize melanoma cells to drugs that activate ROS. Thus, various clinical drugs reported to activate ROS were used to separately treat melanoma cells with or without iron co-treatment. Among them, sulfasalazine (SSZ),^40^ buthionine sulfoximine (BSO),^41^ BAY 87-2243 (BAY),^42^ phenylethyl isothiocyanate (PEITC),^41^ etacrynic acid (EA),^43^ and ezatiostat (TLK199)^43^ could mildly elevate the ROS level in A375 cells, and this ROS elevation was substantially amplified by co-treatment of iron (Fig. 6a; Supplementary information, Fig. S6a). Although none of these drugs alone induced pyroptosis, co-treatment of iron significantly promoted pyroptosis, as revealed by LDH release, GSDME cleavage and cell swelling (Fig. 6a; Supplementary information, Fig. S6a, b). Thus, these data highlight a novel concept in which iron acts as a sensitizer for these ROS-inducing drugs in pyroptosis induction.

Then, we chose SSZ and BSO for a more detailed analysis in A375 cells. SSZ is an inhibitor of cystine-glutamate antiporter, BSO is an inhibitor of γ-glutamylcysteine synthetase, and both inhibit intracellular GSH generation to elevate ROS levels.^40,44^ SSZ/FeSO_4_ or BSO/FeSO_4_ could inhibit the clonogenic survival of A375 cells as efficiently as CCCP/FeSO_4_ or CCCP/Fe_2_(SO_4_)_3_ in the long-term colony formation assays (Supplementary information, Fig. S6c), which indicates that melanoma cells did not develop any obvious adaptation or resistance to this iron-dependent pyroptosis. Co-treatment of SSZ or BSO with iron, but not SSZ or BSO treatment alone, effectively induced the oligomerization and accumulation of Tom20 in a time-dependent manner (Fig. 6b; Supplementary information, Fig. S6d), which resulted in the cleavage of GSDME (Fig. 6c; Supplementary information, Fig. S6e). Moreover, separate knockdown of Tom20, Bax, caspase-3 or GSDME substantially suppressed the SSZ/iron- or BSO/iron-induced pyroptotic features, including GSDME cleavage, LDH release and cell swelling (Fig. 6d–g; Supplementary information, Fig. S6f-i). Again, we provide clear evidence that the Tom20-Bax-caspase-GSDME signaling pathway is indeed involved in iron-induced pyroptosis by activating ROS signaling. However, when either ferroptosis inhibitors (including Ferrostatin-1, U0126, AZD6244, AZD8330 and PD98059) or caspase inhibitor Z-VAD was separately used to pre-treat A375 cells, ferroptosis inhibitors showed slight or even no effect on SSZ/iron- and BSO/iron-induced LDH release (Supplementary information, Fig. S6j). In contrast, Z-VAD significantly blocked SSZ/iron- and BSO/iron-induced LDH release (Supplementary information, Fig. S6j). Thus, these results further rule out the involvement of ferroptosis in SSZ/iron- or BSO/iron-triggered cell death.

### Physiological role of iron in suppressing melanoma development in mouse models

**Fig. 7:**
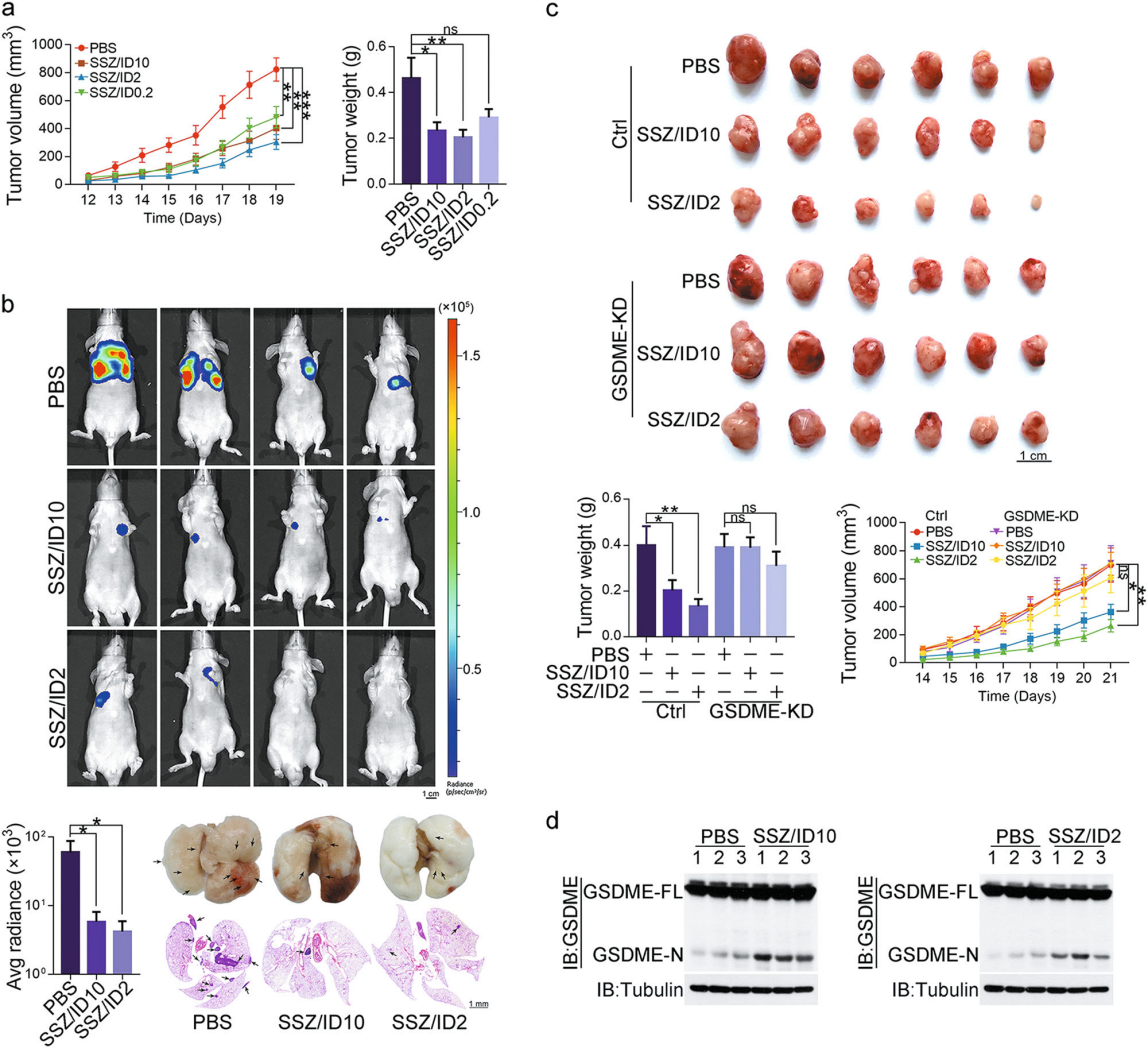
Physiological role of iron in suppressing melanoma development. **a** Melanoma A375 cells were injected subcutaneously into the posterior flanks of nude mice (*n* = 6). After four days, SSZ (50 mg/kg) with or without iron dextran (10 mg/kg, 2 mg/kg and 0.2 mg/kg, respectively) as indicated was intraperitoneally administered to the mice every other day for 2 weeks. The tumor volume and weight were recorded at the indicated times. ID: iron dextran. PBS: phosphate-buffered saline. **b** Administration of SSZ/ID inhibited tumor metastasis in mice. Luciferase-expressing A375 cells (2 × 10^6^ cells/mouse) were injected into the tail veins of nude mice (*n* = 7). Mice were administered with SSZ (50 mg/kg)/ID2 (2 mg/kg) or SSZ (50 mg/kg)/ID10 (10 mg/kg) every other day for 50 days, and tumor metastasis was quantified using bioluminescence imaging. Representative images of mice and luciferase signal intensities were shown. The tumors formed in the lungs were indicated by H&E staining. **c** Effects of GSDME knockdown on tumor growth, tumor volume and weight. GSDME was knocked down in A375 cells and injected subcutaneously into the posterior flanks of nude mice (*n* = 6). The mice were treated with the drugs as described above. Images of xenograft tumors in nude mice, tumor weights and volumes were shown. **d** The cleavage of GSDME was detected in xenograft tumors from the tumor samples (ctrl group) of **c**

To further test the sensitizing effect of iron on SSZ in vivo, nude mice with A375 cell-driven xenograft tumors were intraperitoneally administered with SSZ or iron dextran solution (ID, a commonly used drug in human to treat iron deficiency) alone or combined with different doses of ID. SSZ administration only mildly impaired tumor growth, and ID administration alone showed no effect on tumor growth even at the concentration of 10 mg/kg (Supplementary information, Fig. S7a). However, combined treatment of SSZ with different doses of ID significantly reduced the tumor volume and tumor weight (Fig. 7a). Importantly, a low dose of ID (2 mg/kg) was sufficient to maximize the anti-tumor effect of SSZ, and this dosage of iron is usually used to treat the iron-deficient patients and is much lower than the dosage that causes iron overload.^45^ In addition, this effect of iron could also be observed in the melanoma metastatic mouse model, in which treatment of SSZ/ID significantly reduced the lung metastasis of A375 cells that were injected into the tail vein of nude mice (Fig. 7b). Clearly, iron exerts the synergistic effect with clinical drug to inhibit tumor growth.

To test whether this anti-tumor effect of iron/SSZ is associated with pyropotosis, A375 cells and GSDME-knockdown A375 cells were separately used to generate xenograft tumors in nude mice. SSZ/ID co-treatment significantly reduced A375 cell-driven tumor growth (Fig. 7c), accompanied with the increased GSDME cleavage as compared to the control PBS group (Fig. 7d). However, after GSDME was knocked down, the injection of SSZ with ID could no longer inhibit xenograft tumor growth (Fig. 7c). Therefore, iron sensitized melanoma cells to ROS-inducing drug therapy via GSDME- and pyroptosis-mediated pathways.

It was reported that pyroptosis is an important cause for side effects induced by many chemotherapeutic drugs in normal tissues, including various tissue damages and weight loss.^18^ Indeed, when mice were treated with 5-fluorouracil (5-FU), severe colitis with loss of crypts in the small intestine, extensive colon shortening, spleen atrophy, and weight loss were clearly observed (Supplementary information, Fig. S7b–e). However, these side effects were barely detected in mice that were combinedly treated with SSZ and ID, suggesting better tolerance of mice to this combined treatment (Supplementary information, Fig. S7b–e). In conclusion, the current study not only demonstrates that iron acts as a sensitizer for drugs that activate ROS in vivo, but also suggests that iron supplement could be a promising strategy for the combination therapies of melanoma patients.

## Discussion

**Fig. 8:**
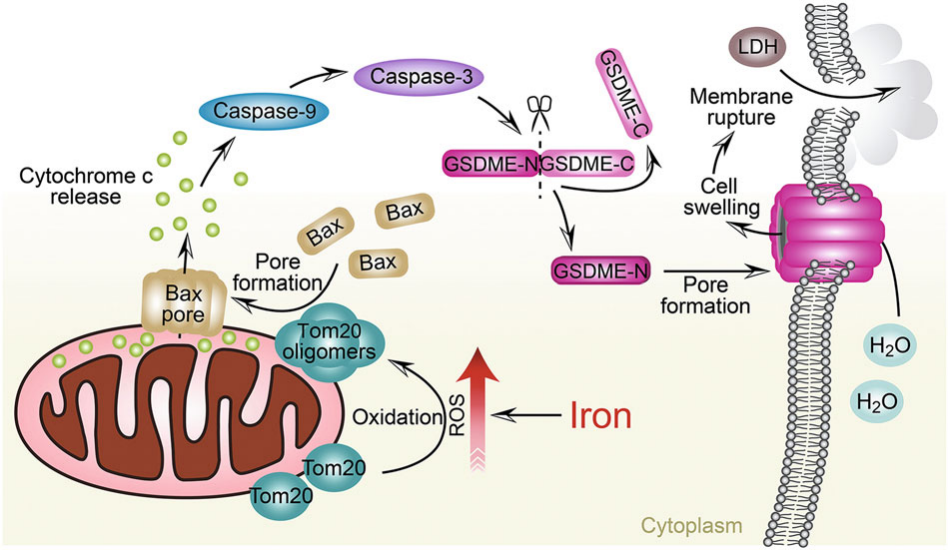
A novel pathway of Tom20-Bax-caspase-GSDME upon iron stimulation. In melanoma cells, iron-elevated ROS causes the oxidation and oligomerization of Tom20. Oxidized Tom20 induces Bax translocation to mitochondria, which facilitates cytochrome c release to cytosol. Once released, cytochrome c activates caspase-9, which then activates caspase-3. This caspase-3 activation further cleaves GSDME, and eventually triggers cell swelling and LDH release

ROS have been recognized to be associated with cancers.^46,47^ Iron, an essential nutrient for life, participates in redox cycling and ROS activation to provoke oxidative cell damage.^4,30^ However, the crosslink between iron-activated ROS and induction of cell death is not completely understood. In this study, we demonstrated that iron amplifies ROS signaling to induce the pyroptotic death of melanoma cells via a novel Tom20-Bax-caspase-3-GSDME pathway. In melanoma cells, the CCCP-initiated ROS were substantially enhanced by iron, leading to the oxidation and oligomerization of Tom20. Tom20 further recruited Bax to mitochondria and caused cytochrome c release to cytosol, consequently activating caspase-3, and ultimately inducing the cleavage of GSDME and pyroptotic death of melanoma cells (Fig. 8). Furthermore, the in vivo assays not only demonstrate that iron is a sensitizer that amplifies ROS signaling to drive the pyroptosis of melanoma cells but also reveals a potential iron-based therapeutic strategy for melanoma.

Iron is thought to induce cell death by elevating ROS.^30^ However, the underlying molecular mechanism of the iron-induced cell death remains unclear. Mitochondria perform multiple critical cellular functions, such as iron metabolism.^48^ We found that CCCP and several chemotherapeutic drugs, such as SSZ, induced ROS, which were further enhanced by iron. Consequently, the mitochondrial signaling pathway was launched, in which Tom20 sensed the ROS signal via oxidative modification at Cys13 and Cys21, leading to accumulation of Tom20. The Tom complex usually recognizes the N-terminal targeting signals of mitochondrial protein precursors to facilitate their mitochondrial import.^49^ Tom20 modification by ROS endows it with novel functions of interacting with and recruiting Bax to mitochondria. After targeting to the outer mitochondrial membrane, Bax promotes mitochondrial aggregation, thereby facilitating cytochrome c release to cytosol, followed by activation of caspase-3, which is a prerequisite for pyroptosis. Therefore, mitochondria may act as a platform connecting the iron-elevated ROS with pyroptosis via Tom20 molecules that sense the intracellular ROS levels. The findings that mitochondrial clearance by mitophagy abolishes iron-induced pyroptosis and that non-Parkin expression in melanoma cells could be beneficial for pyroptosis induction further support this hypothesis.

Although ferroptosis is a recently recognized form of iron- and ROS-dependent necrotic cell death,^50^ ferroptosis appears to be different from iron-related pyroptosis, as demonstrated by the following evidence: (1) rupturing and blebbing of plasma membrane upon CCCP/iron stimulation, which is a characteristic morphology of pyroptosis but not ferroptosis,^18,30^ were clearly observed; (2) ferroptosis could not be blocked by caspase inhibitors,^30^ but the pan-caspase inhibitor Z-VAD efficiently inhibited iron-induced pyroptosis, and caspase activation was shown to be required for pyroptosis induction; (3) activation of RAF/MEK/ERK signaling is important for induction of ferroptosis,^30^ but ERK inhibitors could not influence the iron-induced pyroptotic death of melanoma cells; (4) ferrostatin-1, a potent and selective inhibitor of ferroptosis,^30^ showed no effect on CCCP/iron-induced cell death; and (5) GSDME, an executor of pyroptosis, was significantly cleaved and activated by caspase-3 in response to iron stimulation, which was associated with pyroptosis of melanoma cells. Thus, iron can trigger different types of cell death depending on the activation of different signaling pathways in cells. In fact, in addition to pyroptosis and ferroptosis, iron-induced apoptosis and necroptosis have also been reported.^4,11^

Pyroptosis has long been regarded as caspase-1-mediated monocyte death in response to certain bacterial insults.^12–14^ Recent studies further demonstrated that pyroptosis is not limited to immune cells, and caspase-3 also mediates pyroptosis.^17,18^ Given the executive functions of the gasdermin family members in pyroptosis, the latter is redefined as gasdermin-mediated programmed necrotic cell death.^51^ The interplay between pyroptosis and apoptosis has been recently studied, although controversial conclusions have been reported. On one hand, pyroptosis suppresses the apoptotic pathway in macrophages^14^; on the other hand, pyroptosis occurs as secondary necrosis after apoptosis in certain cells.^17^ In fact, the involvement of caspase-3 in GSDME-dependent pyroptosis suggests that pyroptosis and apoptosis share many signal transduction pathways. The expression level of GSDME may determine whether cells undergo pyroptosis or apoptosis. High GSDME expression could guide cells to pyroptosis before the onset of apoptosis.^18^ Although GSDME is not expressed in many types of cancer cells,^18,52–54^ GSDME is specifically and highly expressed in melanoma tissues^55^ and melanoma cell lines. This high GSDME expression may serve as an Achilles’ heel for melanoma, providing an opportunity for melanoma therapy via pyroptosis induction. Notably, the finding that the common V600 mutation in BRAF in melanoma is irrelevant to pyroptosis reveals the potential application of pyroptosis for melanoma treatment.

GSDME has been reported to be highly expressed in normal tissues, and GSDME-dependent pyroptosis contributes to the toxicity of chemotherapy.^18^ In our case, we observed no obvious side effects in the mice following the combined treatment of ROS-inducing drugs and iron, suggesting that iron has a specific effect on melanoma but not normal tissues. This specificity might be explained by the following: (1) in cancer cells, usually there is upregulation of proteins that are involved in iron uptake, such as transferrin receptor 1 (TfR1), but downregulation of iron efflux proteins, such as ferroportin.^8^ This reprogramming of iron metabolism results in enhanced iron influx in cancer cells. Under this circumstance, stimulating and utilizing iron to induce pyroptosis may be fatal to cancer cells; (2) in cancer cells, in general, the intracellular ROS level increases due to fundamental defects in oxidative metabolism, whereas in normal tissue cells, oxidative stress could be neutralized quickly by the enhanced antioxidant defense system.^46,47^ Therefore, cancer cells may be more sensitive than normal cells to reagents that cause the further accumulation of ROS. Based on the iron- and ROS-dependent features of pyroptosis in our study, unsurprisingly, such iron-related pyroptosis was specifically observed in tumor tissues, at least in melanoma that expresses GSDME.

Diverse drugs currently used in clinical cancer chemotherapy are linked to the induction of oxidative stress.^2^ Because iron amplifies ROS for pyroptosis induction, iron may be a potential candidate for melanoma therapy by serving as a sensitizer that turns on the pro-pyroptotic function of chemotherapeutic drugs. Our in vivo study further supports this hypothesis. Importantly, simply iron supplementation at a dosage used in iron-deficient patients (around 2 mg iron/kg body weight/day)^56^ is sufficient to maximize the anti-tumor effect of these clinical drugs to inhibit xenograft tumor growth and metastasis of melanoma cells without obvious side effects. Because melanoma cells are often resistant to apoptosis and pyroptosis is a type of immunogenic cell death,^57^ this iron-related pyroptosis induction may represent an alternative therapeutic strategy against melanoma. Of course, further studies are warranted to determine the effect of iron-related therapy in other cancers.

## Materials and methods

### Reagents and antibodies

The goat anti-rabbit (Cat# 31210) and anti-mouse (Cat# 31160) secondary antibodies were purchased from ThermoFisher Scientific. The anti-tubulin (Cat# T-4026), anti-p62/SQSTM1 (Cat# WH0008878M1), anti-LC3 (Cat# L-7543), anti-GSDMD (Cat# G7422) and anti-Flag (Cat# F-1804) antibodies were purchased from Sigma. The anti-PARP (Cat# 9532), anti-Tom20 (Cat# 42406 S), anti-Hsp60 (Cat# 12165 S), anti-caspase-9 (Cat# 9502 S), anti-caspase-3 (Cat# 9665), anti-VDAC1 (Cat# 4866) and anti-cytochrome c (Cat# 11940 S) antibodies were purchased from Cell Signaling Technology. The anti-Tom22 (Cat# 11278-1-AP), anti-Tom70 (Cat# 14528-1-AP), and anti-Bax (Cat# 50599-2-Ig) antibodies were purchased from Proteintech. The anti-GSDME (Cat# ab215191) antibody was purchased from Abcam. The anti-Tom40 (Cat# SC-365467) antibody was purchased from Santa Cruz Biotechnology. The anti-active caspase-3 (Cat# 9502 S) antibody was purchased from BD Biosciences. The anti-Bax (N-terminus) (Cat# ABC11) antibody was purchased from Millipore.

The chemical reagents PI (Cat# P4170), DAPI (Cat# D9542), CCCP (Cat# C2759), N-acetyl-L-cysteine (Cat# A7250), etoposide (Cat# E1383), DL-buthionine-sulfoximine (Cat# 19176), 2-phenylethyl isothiocyanate (Cat# 253731), iron dextran (Cat# D8517) and ethacrynic acid (EA, Cat# SML1083) were purchased from Sigma-Aldrich. Z-VAD (Cat# HY-16658), deferoxamine mesylate (Cat# HY-B0988), staurosporine (Cat# HY-15141), doxorubicin (Cat# HY-15142A), actinomycin D (Cat# HY-17559), U0126 (Cat# HY-12031), AZD8330 (Cat# HY-12058), AZD6244 (Cat# HY-50706), sulfasalazine (Cat# HY-14655), BAY-87-2243 (Cat# HY-15836), and TLK199 (Cat# HY-1 3634 A) were purchased from MedChem Express. CM-H2DCFDA (Cat# C6827) and the Annexin V Apoptosis Detection Kit FITC were purchased from ThermoFisher Scientific. Glutathione reduced (Cat# A600229) was purchased from Sangan Biotech. MG132 (Cat# S2619) was purchased from Selleckchem. FeSO_4_ (Cat# 10012118) and Fe_2_(SO4)_3_ (Cat# 10012218) was purchased from Sinopharm Chemical Reagent Co., Ltd. Cycloheximide (Cat# 2112 s) was purchased from Cell Signaling Technology. Hoechst 33342 (Cat# C1022) and DNA Ladder extraction kit (Cat# C0007) were purchased from Beyotime. TNF-α (Cat# 10602-HNAE) was purchased from Sinobiological. The FastQuant RT Kit (Cat# KR106) was purchased from Tiangen Biotech. The CytoTox 96 Non-Radioactive Cytotoxicity Assay (Cat# G1780), GoTaq qPCR Master Mix (Cat# A6101) and Beetle Luciferin (Cat# E1605) were purchased from Promega. The MiniBEST Universal Genomic DNA Extraction Kit Ver.5.0 (Cat# 9765) was purchased from TaKaRa.

Recombinant active Caspase-1 (Cat# ALX-201-056-U025), Caspase-7 (Cat# ALX-201-061-U025), Caspase-8 (Cat# ALX-201-062-U025) and Caspase-9 (Cat# ALX-201-047-U025) were from Enzo Life Sciences. Caspase-3 (Cat# 707-C3-010/CF) was from R&D Systems.

### Cell culture

The human embryonic kidney (HEK293T) cells and human melanoma cell lines A375, SK-MEL-1, M14, ME4405, Mel-11, IgR3, ME4405, A549, Huh-7, MDA-MB-231 and HeLa were cultured in Dulbecco’s Modified Eagle Medium (DMEM)-high glucose (Sigma) supplemented with 10% fetal bovine serum (FBS; GEMINI BIO-PRODUCTS), 100 IU penicillin and 100 mg/mL streptomycin (Bio Basic Inc.). SH-SY5Y cells were maintained in DME/F-12 1:1 (HyClone) supplemented with 10% FBS. During various drug treatments, the concentration of FBS was reduced from 10% to 0.5%. The transfections were performed using Entranster-D-4000 transfection reagent (Engreen Biosystem Co.), except for 293 T cells, which were transfected using the calcium phosphate method.

### Plasmid construction

Using human Tom20 and GSDME cDNA as templates, Tom20 and GSDME were separately cloned into pCDH-CMV-MCS-EF1-Puro using PCR/restriction digest-based cloning. The siRNA-resistant mutants were generated by introducing a silent mutation at the siRNA target site. The Tom20 (C13S, C21S, C100S) and GSDME (D267A, D270A) mutants were generated using a QuikChange mutagenesis kit (Stratagene) and verified by sequencing.

### Generation of the lentiviral system

The lentiviral system was generated as previously described.^58^ The oligonucleotides (Invitrogen) were annealed and subcloned into the lentiviral vector pLL3.7 or pLKO.1. The lentiviruses were generated by transfecting subconfluent HEK293T cells together with the lentiviral vector and packaging plasmids. The viral supernatants were collected after 48 h and then infected into cells. The knockdown efficiency of the target genes was determined by western blotting or RT-PCR.

The oligonucleotide sequences for the pLL3.7 shRNAs were as follows:

shRNA-Tom20, 5′-GCTAGCTGTGTCGAGTTAA-3′;

shRNA-Tom40, 5′-GGAAATACACATTGAACAA-3′;

shRNA-GSDME, 5′-GCAGCAAGCAGCTGTTTAT-3′;

shRNA-Caspase-3, 5′-GCAGCAAACCTCAGGGAAA-3′;

shRNA-Caspase-9, 5′-GCAAAGTTGTCGAAGCCAA-3′;

shRNA-GSDMD, 5′-GCAGGAGCTTCCACTTCTA-3′;

shRNA-BAX, 5′-GCTGGACATTGGACTTCCT-3′;

shRNA-DMT1, 5′- GGATTGCAGGAGGAATCTT-3′;

shRNA-TfR1, 5′- GCTGGTCAGTTCGTGATTA-3′;

shRNA-TfR2, 5′- GCTCAGAGCCAGATCACTA-3′; and

shRNA-Control, 5′-GCGCGCTTTGTAGGATTCG-3′.

The oligonucleotide sequences for the pLKO.1 shRNAs were as follows:

shRNA-FTH1, 5′-GCCGAATCTTCCTTCAGGATA-3′;

shRNA-FTL, 5′-CTGGAGACTCACTTCCTAGAT-3′; and

shRNA-Control, 5′-CAACAAGATGAAGAGCACCAA-3′.

### RT-PCR and primers

Total RNA was extracted using a total RNA isolation kit (Tiangen, BJ, China) and reverse transcribed using a reverse transcriptase kit (Tiangen, BJ, China). cDNA was used as a template for the amplification, and the level of actin was used as a normalization control. The primer sequences used were as follows (5′-3′):

FTH1: forward, AGCTCTACGCCTCCTACGTT;

reverse, AAGGAAGATTCGGCCACCTC.

FTL: forward, GCTCACTCTCAAGCACGACT;

reverse, AAGCTGCCTATTGGCTGGAG.

DMT1: forward, ACCTAGGGCATGTGGCATTA;

reverse, GAGATGCTTACCGTATGCCC.

TfR1: forward, GGCTGTATTCTGCTCGTGGA;

reverse, CCCCAGAAGACATGTCGGAAA.

TfR2: forward, GCCCCCTTCTGACAAGTCTC;

reverse, AAGGCCGTGAAGGAATAGGC.

Parkin: forward, AGTGTTTGTCAGGTTCAACTCCAGC;

reverse, AACCCCCTGTCGCTTAGCAAC.

Actin: forward, CAGCCTTCCTTCCTGGGCATG;

reverse, ATTGTGCTGGGTGCCAGGGCAG.

GAPDH: forward, CATGTTCGTCATGGGTGTGAACCA;

reverse, AGTGATGGCATGGACTGTGGTCAT.

Total genomic DNA was extracted using a TaKaRa MiniBEST Universal Genomic DNA Extraction Kit Ver.5.0. The mitochondrial DNA content was determined by RT-PCR and normalized using the single-copy nuclear gene α-globin. The following primers were used (5′–3′):

mtDNA: forward, CACCAGCCTAACCAGATTTC;

reverse, GGGTTGTATTGATGAGATTAGT.

α-globin: forward, GCTTCTGACACAACTGTGTTCACTAGC;

reverse, CACCAACTTCATCCACGTTCACC.

### Immunoprecipitation and western blot analysis

Immunoprecipitation was performed as described previously.^59^ The cells were lysed in buffer containing 50 mM Tris (pH 7.5), 150 mM NaCl, 1 mM EDTA, 1 mM EGTA, 2.5 mM sodium pyrophosphate, 1% Triton X-100 and 1 mM PMSF. The proteins were immunoprecipitated from the cell lysates by incubating the lysates with the appropriate antibodies overnight at 4 °C and subsequently with protein A/G agarose beads for another 1 h at 4 °C. Then, the immunoprecipitates were collected and washed three times with lysis buffer, and a western blot analysis was performed. For the oxidation analysis, 100 mM N-ethylmaleimide (NEM) were added to the lysis buffer to block the free thiols. The reduced or nonreduced samples were prepared with or without dithiothreitol (DTT). The samples were subjected to SDS-PAGE and transferred to polyvinyl difluoride (PVDF) membranes, which were probed with the appropriate antibodies and detected by enhanced chemiluminescence (Pierce).

### GSDME cleavage assay

HEK293T cells were transiently transfected with GSDME-Flag. After 24 h, the GSDME proteins were immunoprecipitated using an anti-Flag antibody and protein A/G agarose beads. GSDME-flag was then eluted with 3× Flag peptide. The eluents were mixed with 2× caspase reaction buffer (100 mM HEPES, 2 mM EDTA, 200 mM NaCl, 20 mM DTT, 20% glycerol, pH 7.5) at a ratio of 1:1. Caspases-1, -3, -7, -8, and -9 were reconstituted according to the manufacturer’s protocols. The recombinant active caspase (2 μL) was added to eluents and incubated at 37 °C for 1 h. The final concentrations of caspases were 7 ng/mL for caspase-3 and 1 unit for caspases-1, -7, -8, and 9. Cleavage of GSDME was detected by western blot.

### Microscopy

To examine the morphology of the pyroptotic cells, the cells were seeded in 6-well plates at ~40%-60% confluency and then treated with different drugs. Phase contrast cell images were captured under a Nikon TE2000 microscope.

For confocal microscopy, the cells were washed with DMEM and fixed in 4% paraformaldehyde, followed by blockade with blocking buffer (3% BSA and 0.2% Triton X-100). The cells were incubated with the appropriate primary antibody overnight at 4 °C, washed with washing buffer (0.2% BSA and 0.05% Triton X-100) and incubated with FITC- or Texas Red-conjugated secondary antibodies (Life Technologies) for another 1 h at 37 °C in the dark. The cells were stained with 4′,6-diamidino-2-phenylindole (DAPI, 50 μg/mL) for 5 min to indicate the nuclei. Images were captured under a Zeiss LSM 780 confocal microscope.

For nuclear staining, the cell nuclei were stained with Hoechst33342 in the dark for 10 min at room temperature and the cells were imaged using a Zeiss LSM 780 confocal microscope.

### Cell fractionation

Cell fractionation was performed as previously described.^60^ Briefly, the cells were scratched from the dish, collected in ice-cold PBS, and washed twice with ice-cold PBS. Then, the cells were resuspended in 200 μL of digitonin lysis buffer (75 mM NaCl, 1 mM NaH_2_PO_4_, 8 mM Na_2_HPO_4_, 250 mM sucrose, and 190 mg/mL digitonin). After 5 min on ice, the cells were spun for 5 min at 12,000× *g* at 4 °C. The supernatants were collected, and the cytosol cytochrome c level was analyzed by western blotting. The pellets were collected, and mitochondrial Bax was analyzed by western blotting.

### Cell survival rate

The cell survival rate was measured by staining the cells with PI. Briefly, the cells were harvested and resuspended in 1 mL PBS containing 5 μg PI. The PI incorporation was quantified using an FC500 flow cytometer (Beckman). PI-negative cells of normal size were considered viable cells.

For Annexin V/PI co-staining, the cells were collected, washed twice with PBS and stained using an Annexin V-FITC/PI apoptosis detection kit according to the manufacturer’s instructions. The stained cells were analyzed using a BD Fortessa flow cytometer.

### Intracellular ROS

The ROS levels were measured using the CM-H2DCFDA reagent. The cells were incubated with CM-H2DCFDA at a final concentration of 10 μM in FBS-free DMEM for 15 min at 37 °C in the dark, washed three times with FBS-free DMEM and resuspended in ice-cold PBS for the analysis of ROS using an FC500 (Beckman) flow cytometer.

### LDH release assay

Pyroptosis was analyzed by detecting the activity of LDH released into cell culture supernatants using the CytoTox 96 Non-Radioactive Cytotoxicity Assay Kit (Promega) according to the manufacturer’s protocol.

### Depletion of mitochondria

In mammalian cells, mitochondria can be depleted by enforced mitophagy. First, Flag-Parkin was stably expressed in cells through viral infection. Then, Flag-Parkin-expressing cells were seeded in 6-well plates. In the following day, the cells were treated with 15 μM CCCP at 90%-100% confluency in complete medium every 12 h; after 36 h, the medium was refreshed. The constructed cells were used in further experiments. The efficiency of mitochondrial depletion was determined by detecting mitochondrial proteins and quantifying the mtDNA abundance.

### Mouse models

Male nude mice (BALB/c, 18–22 g, 7–8 weeks old) were obtained from the SLAC Laboratory Animal Center, China, and maintained at the Laboratory Animal Center at Xiamen University (China) in a facility on a 12-h light-dark cycle with free access to food and water in accordance with institutional guidelines. All animal experiments were approved by the Animal Ethics Committee of Xiamen University (approval no: XMULAC20120030).

To generate the xenograft tumor model, 2 × 10^6^ A375 cells were suspended in 100 μL of DMEM and injected subcutaneously into either of the anterior flanks of the mice. Four days after the inoculation, the mice were divided into different groups for the treatments of vehicle (PBS, 100 μL per mouse), SSZ (50 mg/kg), SSZ (50 mg/kg) plus iron dextran (0.2–10 mg/kg), which were administered via intraperitoneal injections every other day for 2 weeks. The mice were sacrificed, and the body weights and tumor weights were recorded. The SSZ solution was freshly prepared in 0.1 M NaOH and subsequently adjusted with 1 M HCl to pH of ~7.4, and the solution was diluted in PBS. Iron dextran was diluted in PBS.

For the lung metastasis experiments, 1.5 × 10^6^ luciferase-expressing A375 cells were harvested in PBS and subsequently injected into the lateral tail veins of the mice in a volume of 200 μL. The treatments described above were performed by intraperitoneal injections every other day for 50 days. The resulting metastases were detected using an IVIS@ Lumina II system (Caliper Life Sciences, Hopkinton, MA, USA) 10 min after intraperitoneally injecting 3 mg D-luciferin (15 mg/mL in PBS).

To analyze side effects of SSZ and ID on mice, C57BL/6 J mice were divided into 3 groups and intraperitoneally injected with vehicle (PBS, negative control) or SSZ (50 mg/kg) together with ID (2 mg/kg) every other days for two weeks. 5-FU (250 mg/kg, positive control) was intraperitoneally injected into mice continually for five days. The mice were weighed and killed, and various tissue damages and weight loss were observed and recorded.

### Hematoxylin and eosin staining

The lungs were removed, fixed in a solution of 4% paraformaldehyde, embedded in paraffin, sectioned at a thickness of 5 μm and placed on slides. The paraffin sections were dried at 65 °C for 2 h. The tissues were deparaffinized using xylene and ethanol for further staining with hematoxylin and eosin (H&E).

### DNA laddering assay

Cells were lysed, and DNA was then extracted and purified with DNA extraction kit (Beyotime, C0007) according to the manufacturer’s instructions. Equal amounts of purified DNA were applied to electrophoresis on a 1.0% agarose gel at 100 V (0.5 h, room temperature). DNA bands were visualized by UV light and photographed.

### Long-term colony formation assay

Cells were seeded into 60 mm dish (3 × 10^4^ cells per dish) and cultured for 10 days. Then the cells were fixed, stained with crystal violet and photographed.

### Statistical analysis

The data are expressed as the mean ± SEM, and the statistical analysis of differences between two groups was performed using two-tailed Student’s *t*-test. The differences between multiple groups were analyzed using one-way or two-way ANOVA, followed by Tukey’s post hoc test. The statistical analysis was performed using GraphPad Prism 6. *P < 0.05 was considered statistically significant, **P < 0.01 was considered highly significant, and ***P < 0.001 was considered extremely significant.

## Electronic supplementary material

Supplementary information, Figure S1

Supplementary information, Figure S2

Supplementary information, Figure S3

Supplementary information, Figure S4

Supplementary information, Figure S5

Supplementary information, Figure S6

Supplementary information, Figure S7

Supplementary movie S1 legend

Supplementary information, Movie S1
