# Supplementary material for: Tom20 senses iron-activated ROS signaling to promote melanoma cell pyroptosis
Source: Cell Res. 2018 Oct 4;28(12):1171–85. doi: 10.1038/s41422-018-0090-y (PMC6274649; doi:10.1038/s41422-018-0090-y)
Supplement: Supplementary file 1 — Supplementary information, Figure S1 [file 41422_2018_90_MOESM1_ESM.pdf]

Supplementary Figure 1

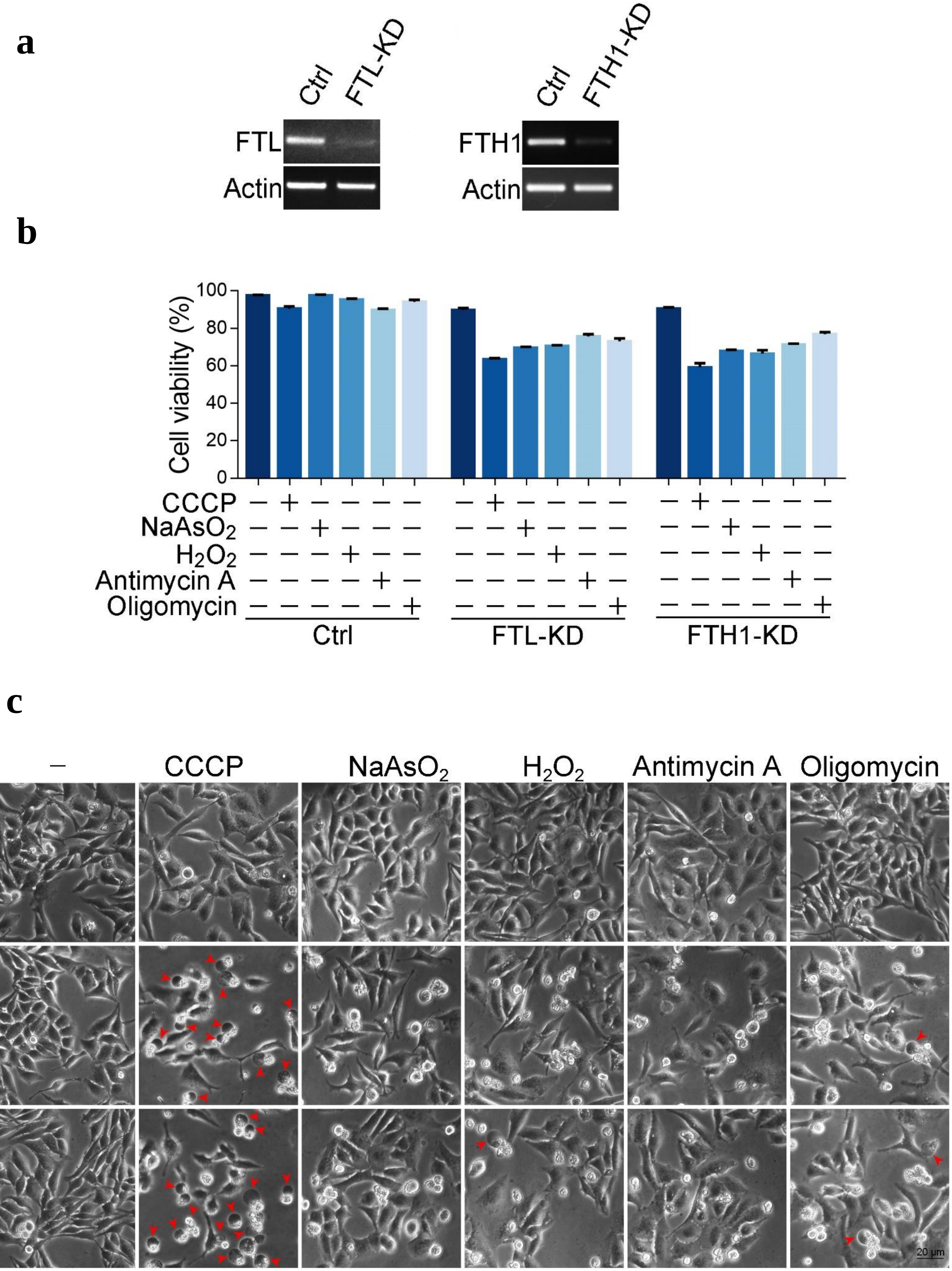

d

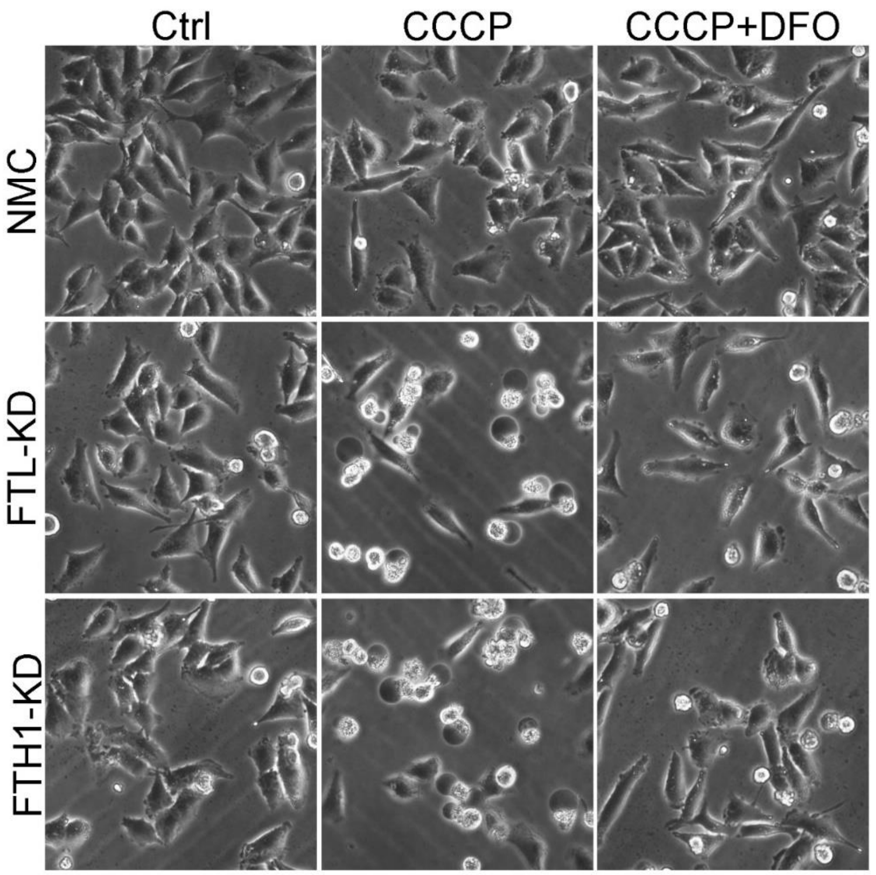
Ctrl
CCCP
CCCP+DFO
NMC
FTL-KD
FTH1-KD

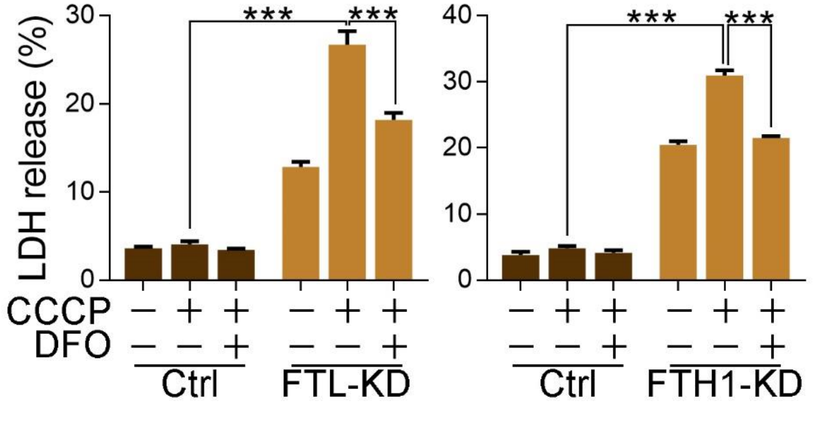
LDH release (%)
30
20
10
0
40
***
CCCP
DFO
Ctrl
FTL-KD
FTH1-KD

e

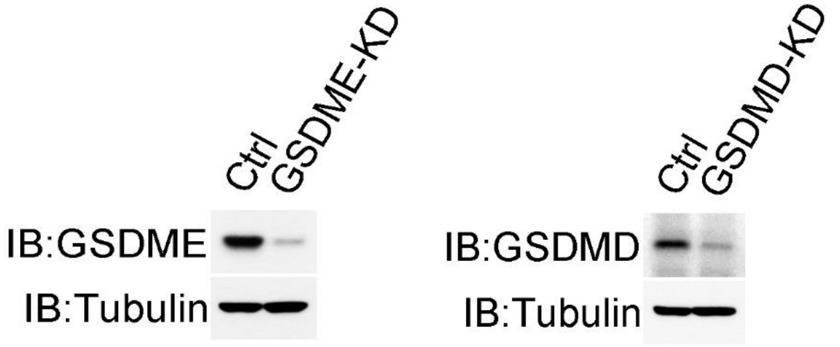
Ctrl
GSDME-KD
IB:GSDME
IB:Tubulin
GSDMD-KD
IB:GSDMD

f

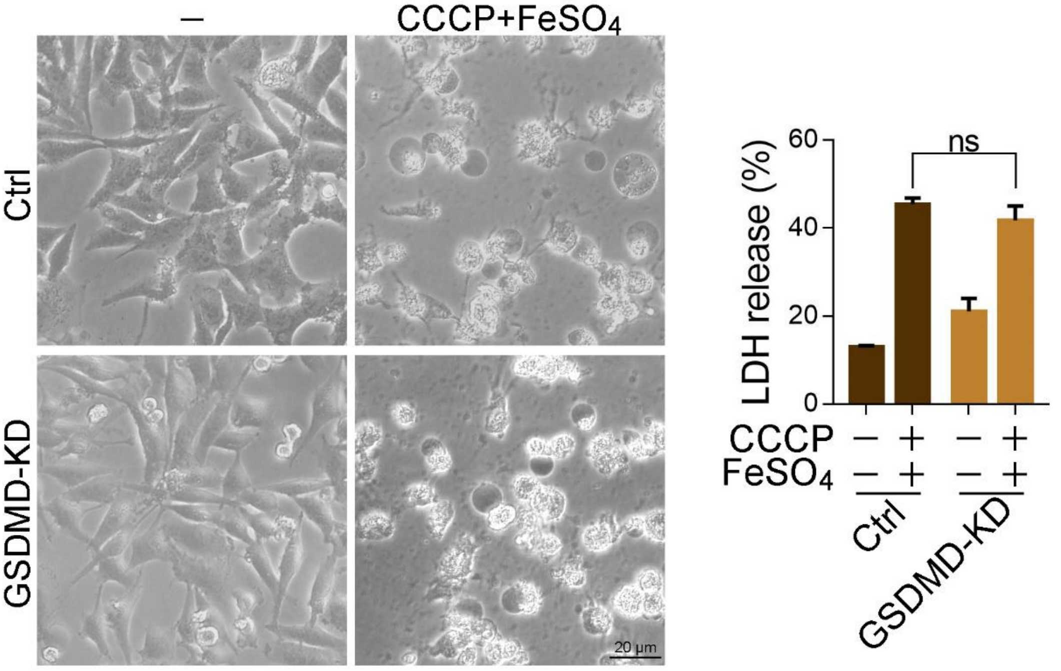
−
CCCP+FeSO4
Ctrl
GSDMD-KD
20 μm
LDH release (%)
60
40
20
0
ns
CCCP − + − +
FeSO4 − + − +
Ctrl
GSDMD-KD

g

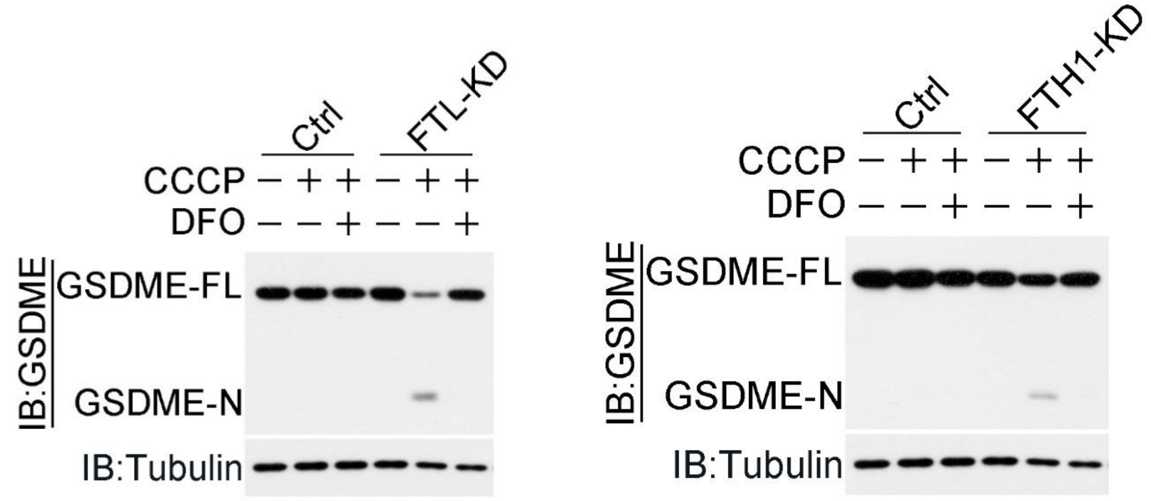
Ctrl
FTL-KD
CCCP − + + − + +
DFO − − + − − +
IB:GSDME
GSDME-FL
GSDME-N
IB:Tubulin
Ctrl
FTH1-KD
CCCP − + + − + +
DFO − − + − − +
IB:GSDME
GSDME-FL
GSDME-N
IB:Tubulin

h

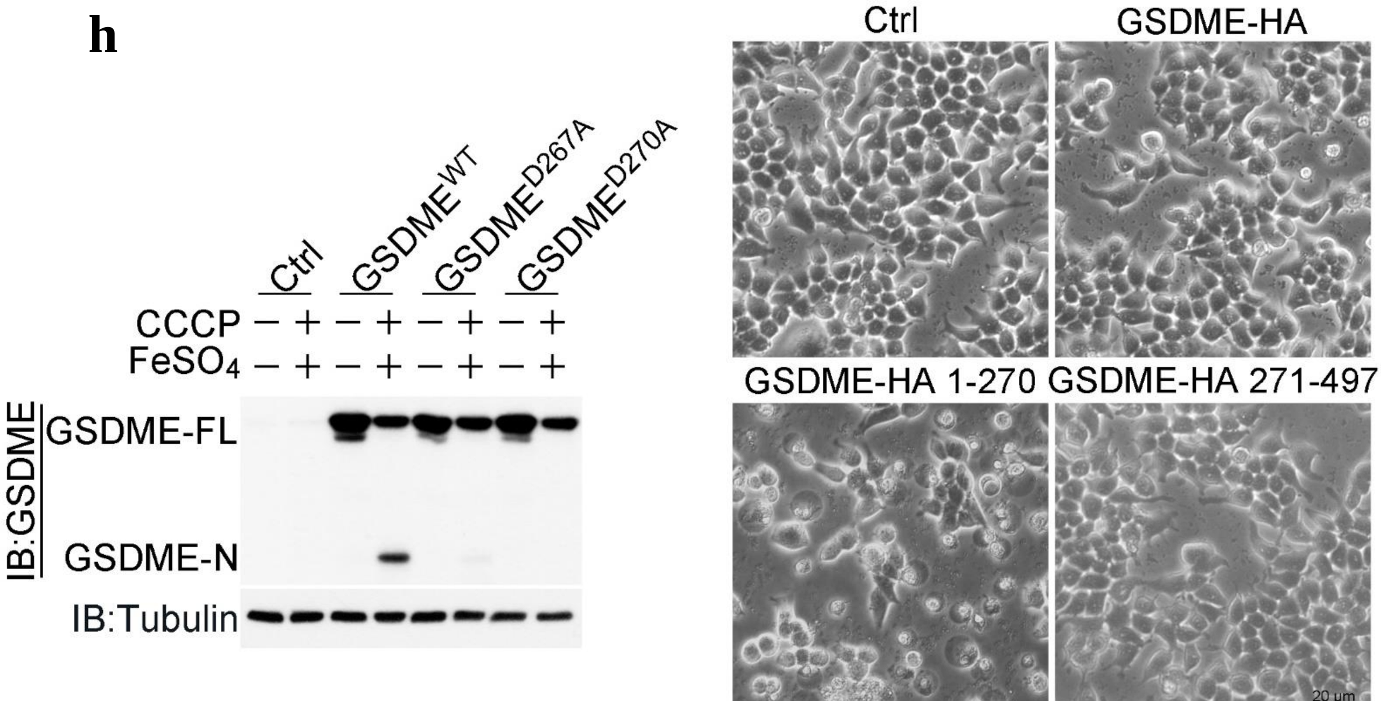
Ctrl
GSDME^WT
GSDME^D267A
GSDME^D270A
CCCP − + − + − + − +
FeSO4 − + − + − + − +
IB:GSDME
GSDME-FL
GSDME-N
IB:Tubulin
Ctrl
GSDME-HA
GSDME-HA 1-270
GSDME-HA 271-497
20 μm

i

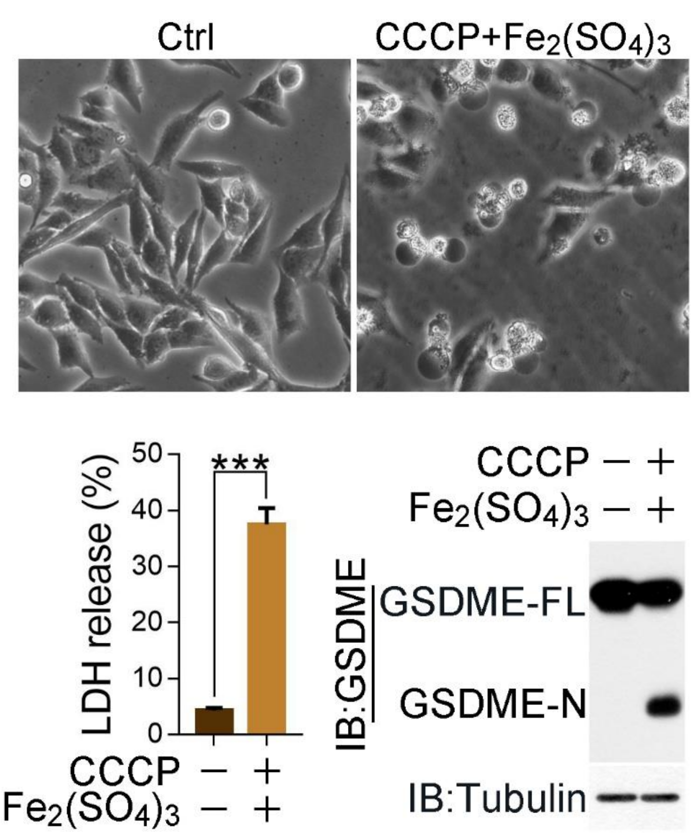

Ctrl
CCCP+Fe2(SO4)3
LDH release (%)
50
40
30
20
10
0
***
CCCP − +
Fe2(SO4)3 − +
CCCP − +
Fe2(SO4)3 − +
IB:GSDME
GSDME-FL
GSDME-N
IB:Tubulin

j

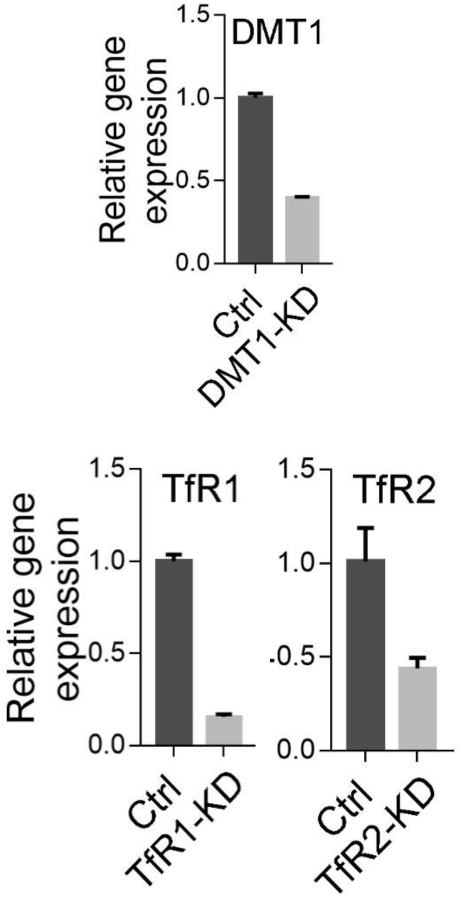

DMT1
Relative gene expression
1.5
1.0
0.5
0.0
Ctrl
DMT1-KD
TfR1
TfR2
Relative gene expression
1.5
1.0
0.5
0.0
Ctrl
TfR1-KD
Ctrl
TfR2-KD

k

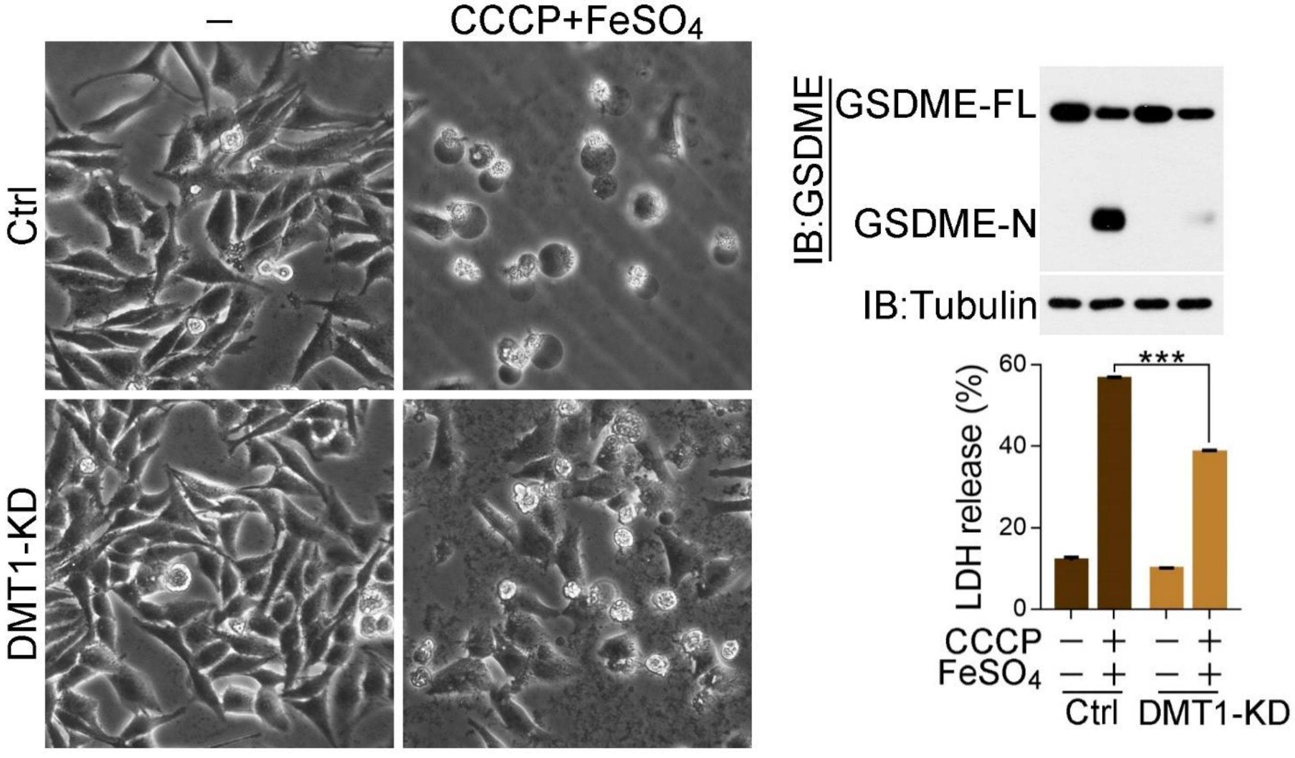

−
CCCP+FeSO4
Ctrl
DMT1-KD
IB:GSDME
GSDME-FL
GSDME-N
IB:Tubulin
LDH release (%)
60
40
20
0
***
CCCP − + − +
FeSO4 − + − +
Ctrl
DMT1-KD

l

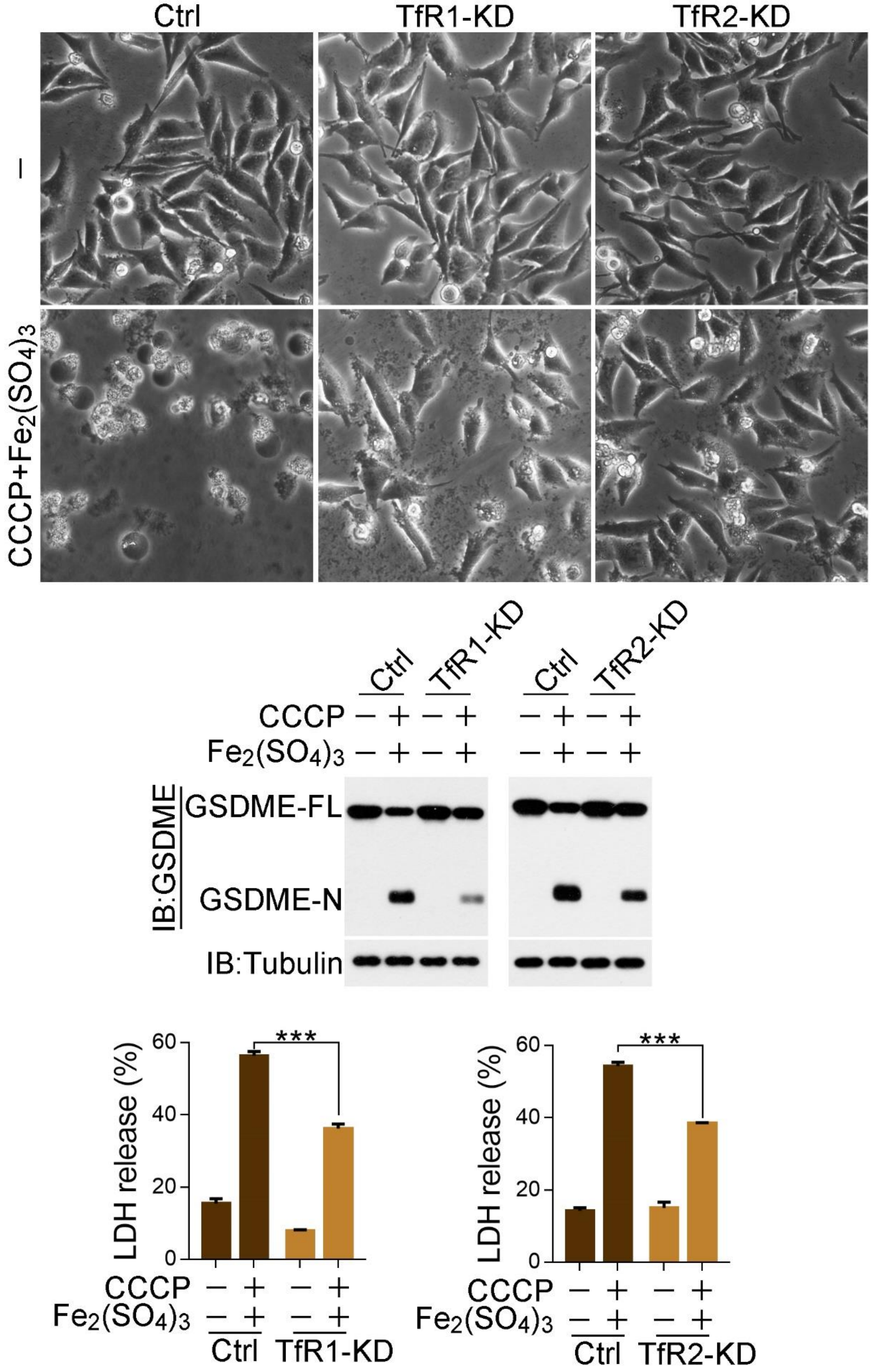
Ctrl
TfR1-KD
TfR2-KD
–
CCCP+Fe2(SO4)3
Ctrl
TfR1-KD
Ctrl
TfR2-KD
CCCP
Fe2(SO4)3
IB:GSDME
GSDME-FL
GSDME-N
IB:Tubulin
LDH release (%)
60
40
20
0
***
CCCP
Fe2(SO4)3
Ctrl
TfR1-KD
LDH release (%)
***
CCCP
Fe2(SO4)3
Ctrl
TfR2-KD

m

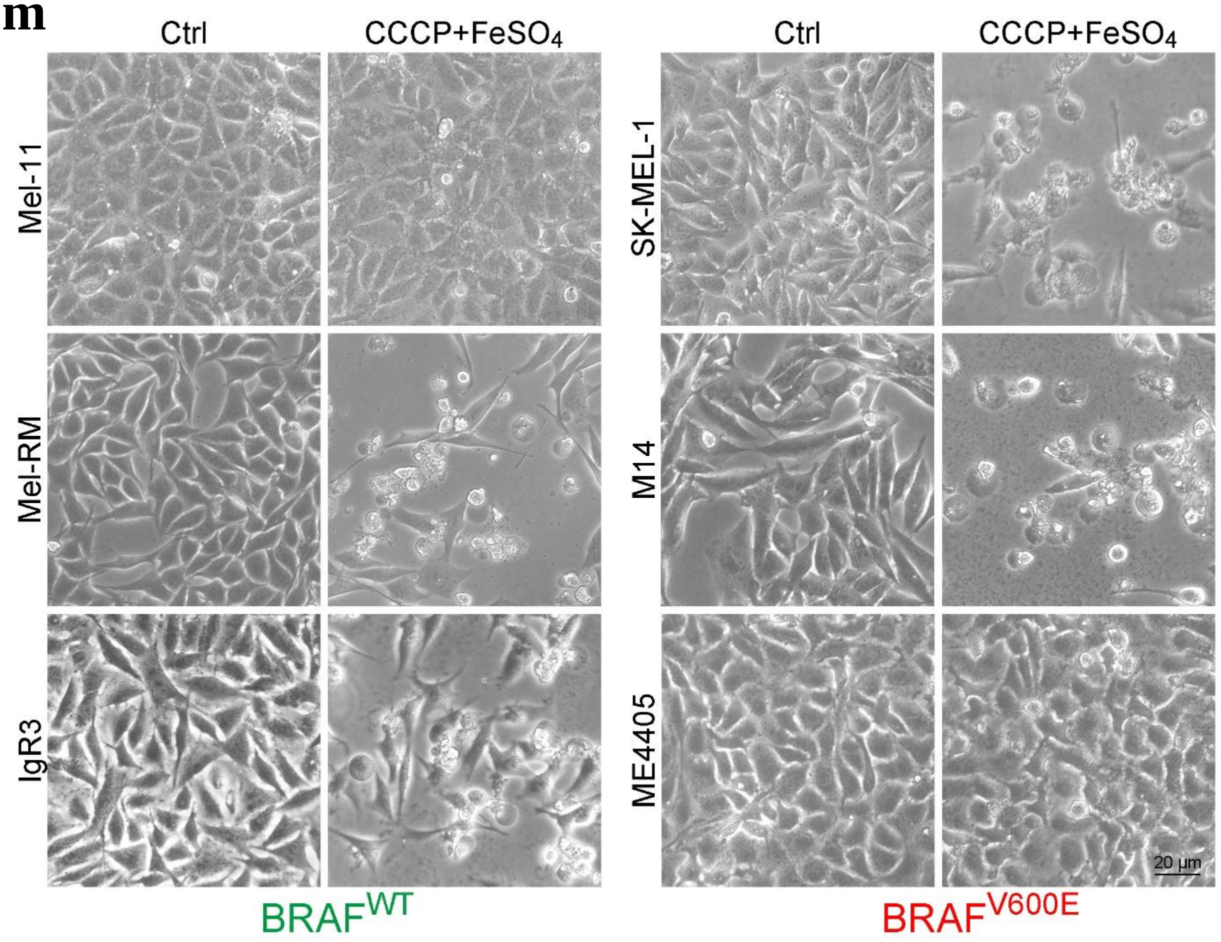

Ctrl
CCCP+FeSO4
Ctrl
CCCP+FeSO4
Mel-11
Mel-RM
IgR3
SK-MEL-1
M14
ME4405
20 μm
BRAF^WT
BRAF^V600E

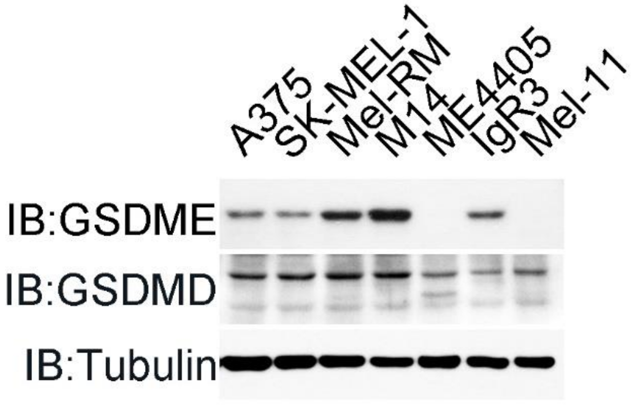

A375
SK-MEL-1
Mel-RM
M14
ME4405
IgR3
Mel-11
IB:GSDME
IB:GSDMD
IB:Tubulin

n

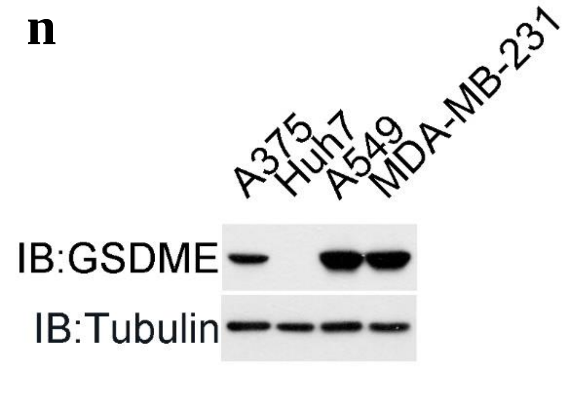

A375
Huh7
A549
MDA-MB-231
IB:GSDME
IB:Tubulin

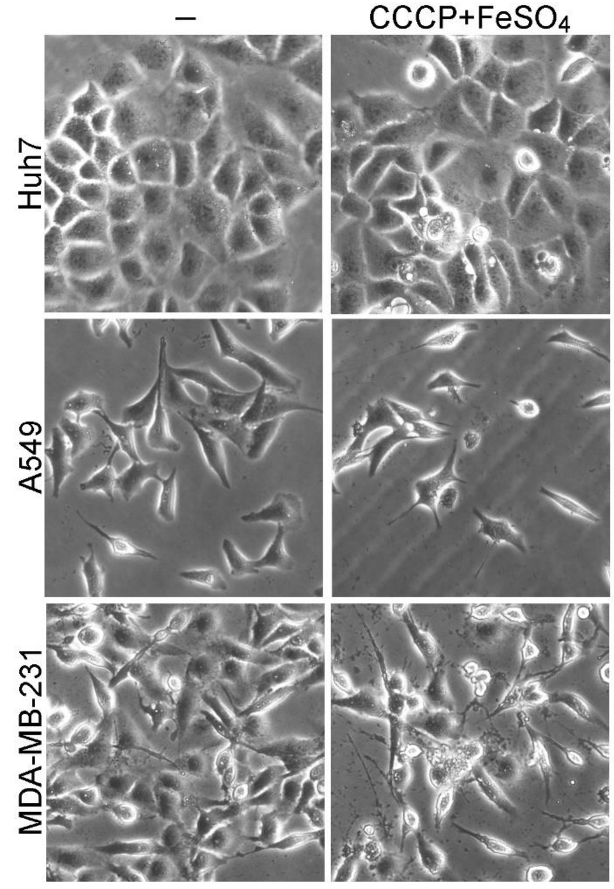

–
CCCP+FeSO4
Huh7
A549
MDA-MB-231

o

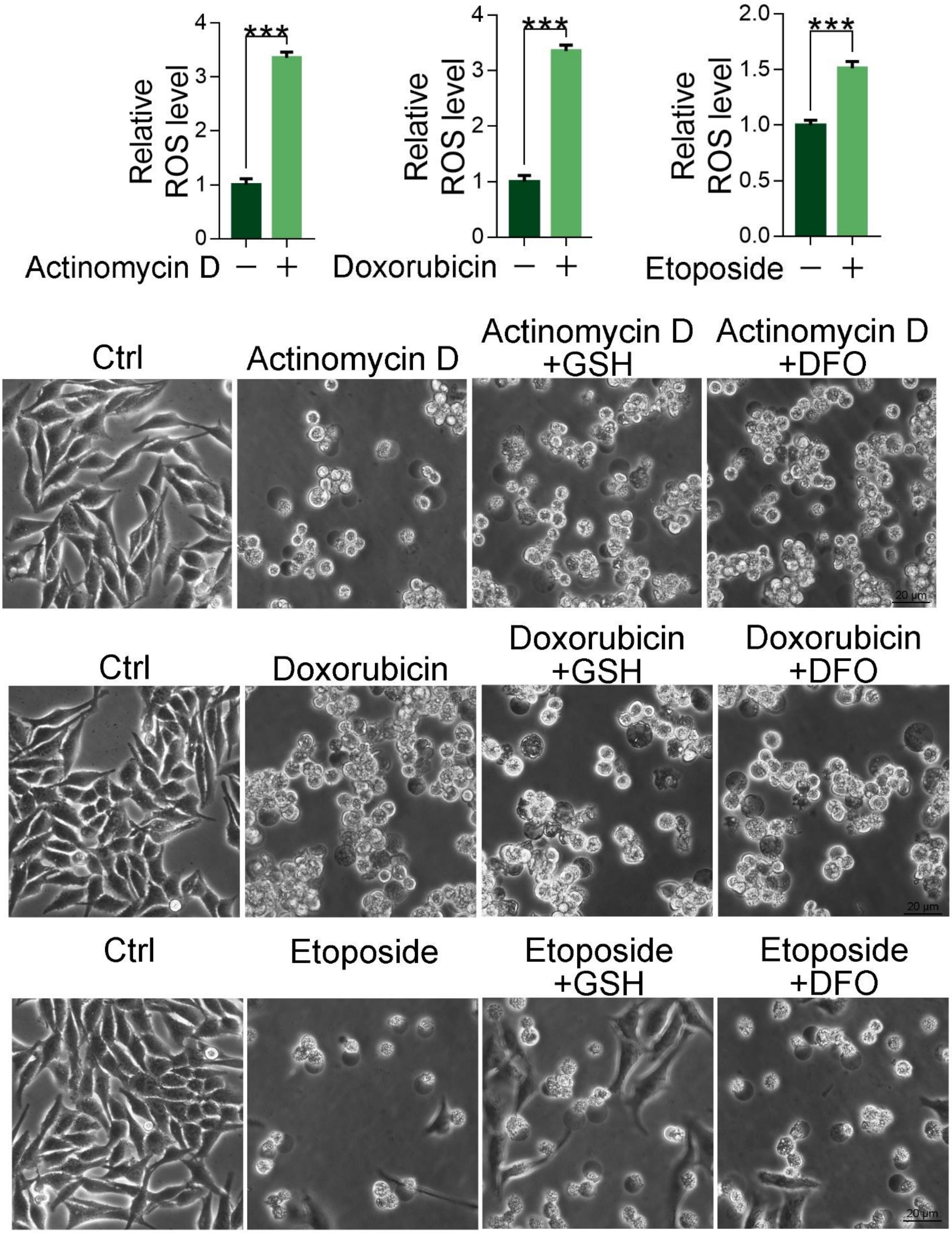

Relative ROS level
4
3
2
1
0
***
Actinomycin D − +
Relative ROS level
4
3
2
1
0
***
Doxorubicin − +
Relative ROS level
2.0
1.5
1.0
0.5
0.0
***
Etoposide − +
Ctrl
Actinomycin D
Actinomycin D +GSH
Actinomycin D +DFO
20 μm
Ctrl
Doxorubicin
Doxorubicin +GSH
Doxorubicin +DFO
20 μm
Ctrl
Etoposide
Etoposide +GSH
Etoposide +DFO
20 μm

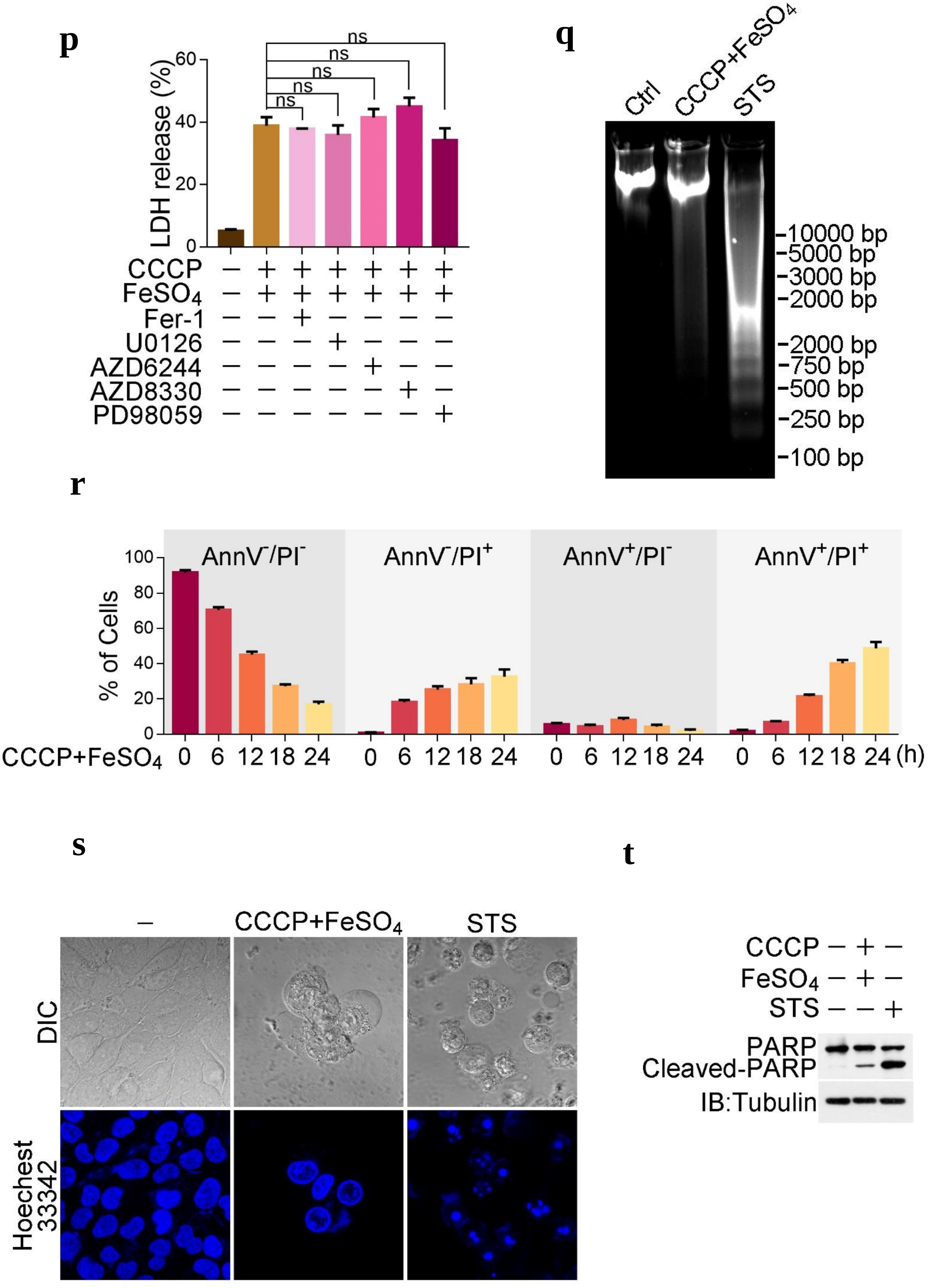

p
LDH release (%)
60
40
20
0
ns
ns
ns
ns
ns
CCCP
FeSO4
Fer-1
U0126
AZD6244
AZD8330
PD98059
q
Ctrl
CCCP+FeSO4
STS
10000 bp
5000 bp
3000 bp
2000 bp
2000 bp
750 bp
500 bp
250 bp
100 bp
r
AnnV-/PI-
AnnV-/PI+
AnnV+/PI-
AnnV+/PI+
% of Cells
100
80
60
40
20
0
CCCP+FeSO4
0 6 12 18 24
(h)
s
–
CCCP+FeSO4
STS
DIC
Hoechest 33342
t
CCCP
FeSO4
STS
PARP
Cleaved-PARP
IB:Tubulin

u

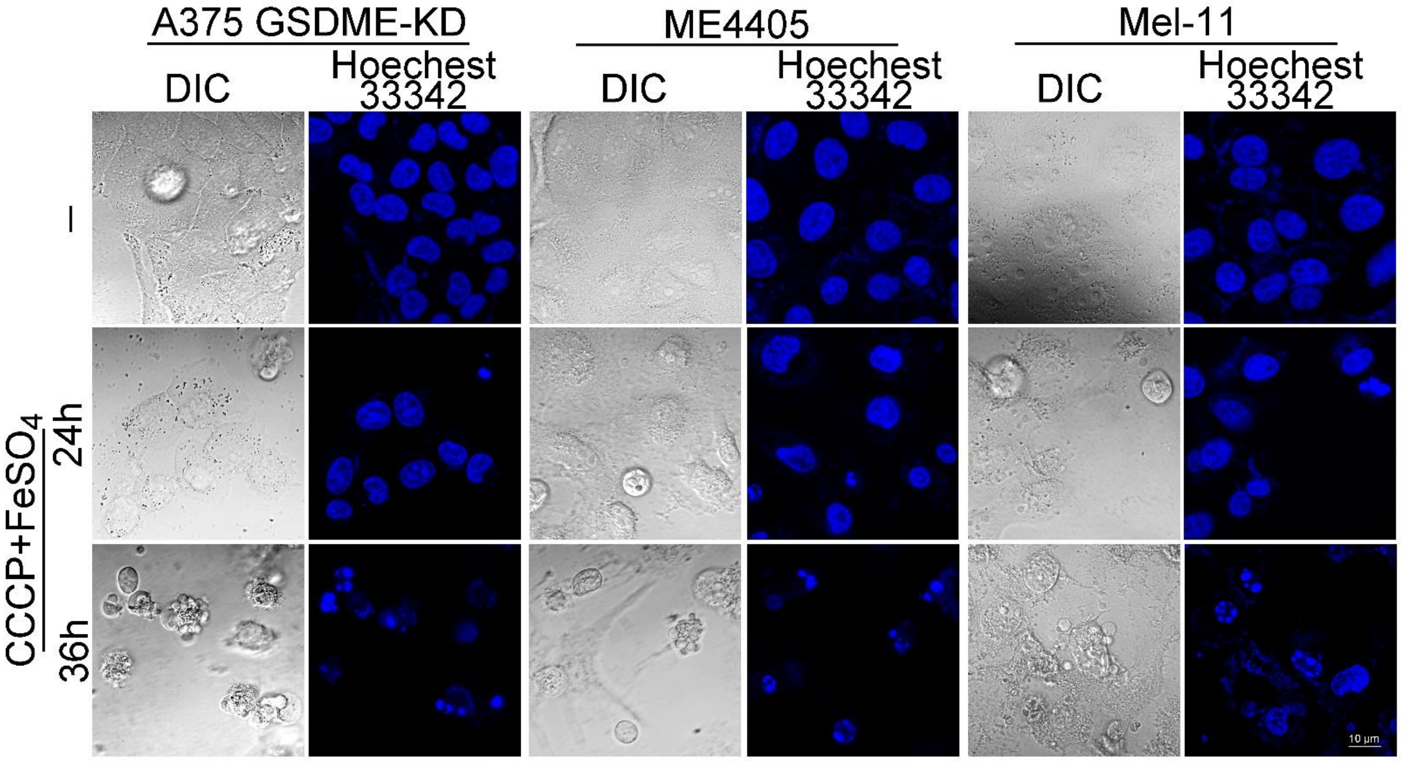
A375 GSDME-KD
ME4405
Mel-11
DIC
Hoechest 33342
DIC
Hoechest 33342
DIC
Hoechest 33342
–
CCCP+FeSO4
24h
36h
10 μm

Figure S1. Melanoma A375 cells were treated with CCCP (20 μM), $FeSO_4$ (100 μM), or CCCP/$FeSO_4$ for 24 h to assess the pyroptotic features (including pyroptotic morphology, GSDME cleavage, and LDH release) and the GSDME expression levels, unless specially defined. **a** Efficiencies of the FTL or FTH1 knockdown in the cells as detected by RT-PCR. **b, c** Effects of different reagents as indicated on cell viability **(b)** and pyroptotic morphology **(c)** in FTL- or FTH1-knockdown cells. CCCP (20 μM), $NaAsO_2$ (2.5 μM), H_2O_2 (50 μM), antimycin A (5.0 μM), or oligomycin (1.0 μM) was used to treat the FTL- or FTH1-knockdown cells for 24 h. **d** The FTL- or FTH1-knockdown cells were pretreated with or without DFO (50 μM) for 2 h before CCCP stimulation. The pyroptotic morphology and LDH release were indicated. **e** Efficiencies of the GSDME and GSDMD knockdown in the cells. **f** Knockdown of GSDMD had no effect on CCCP/$FeSO_4$-induced pyroptosis and LDH release. **g** The FTL- or FTH1-knockdown cells were pretreated with or without DFO (50 μM) for 2 h before CCCP stimulation. The cleavages of GSDME were indicated. **h** Left, point mutation of GSDME at Asp267 ($GSDME^{D267A}$) or Asp270 ($GSDME^{D270A}$) to alanine abolished CCCP/$FeSO_4$-induced GSDME cleavage. Right, GSDME deletion with a.a. 1 to 270 (GSDME HA 1-270), but not a.a. 271 to 497 (GSDME HA-271-497), induced pyroptotic morphology in the 293T cells. **i** $Fe_2(SO_4)_3$-induced pyroptotic morphology, LDH release and GSDME cleavage in the presence of CCCP. The cells were treated with CCCP plus $Fe_2(SO_4)_3$ (50 μM) for 24 h. **j** Efficiencies of the DMT1, TfR1 and TfR2 knockdown in the cells. **k, l** Knockdown of DMT1 **(k)**, TfR1 and TfR2 **(l)**, respectively, attenuated CCCP/$FeSO_4$- or CCCP/

$Fe_2(SO_4)_3$-induced pyroptotic morphology, LDH release and GSDME cleavage in the cells. **m** Effects of CCCP/$FeSO_4$ on different melanoma cell lines, including $BRAF^{WT}$ and $BRAF^{V600E}$ mutation. Pyroptotic morphology in cells (top) and expression levels of GSDME and GSDMD (bottom) were shown post 24 h of CCCP/$FeSO_4$ treatment. **n** Effects of CCCP/$FeSO_4$ on different types of cancer cell lines, including Huh7 (hepatoma cells), A549 (lung cancer cells) and MDA-MB-231 (breast cancer cells). The expression levels of GSDME (left) and pyroptotic morphology (right) were shown post 24 h of CCCP/$FeSO_4$ treatment. **o** Effects of different reagents, including actinmyxin D (10 μg/mL), doxorubicin (20 μM) and Etoposide (40 μM), on the ROS level and pyroptotic morphology, with or without GSH or DFO pretreatment. The cells were pretreated with GSH (1 mM) or DFO (50 μM) for 2 h, followed by treatment of different reagents for 24 h, respectively, as indicated. **p** Inhibitors of ferroptosis (ferrostatin-1 (0.5 μM), U0126 (20 μM), AZD6244 (5 μM), AZD8330 (2.5 μM) and PD98059 (5 μM)) could not impair the CCCP/$FeSO_4$-induced LDH release. Cells were pretreated with or without different inhibitors as indicated for 2 h, followed by treatment of CCCP/$FeSO_4$ for 24 h to detect the levels of LDH. Fer-1: ferrostatin-1. **q** CCCP/$FeSO_4$ showed no effect on DNA laddering. The cells were treated with CCCP/$FeSO_4$ for 24 h, and DNA was prepared. STS (2 μM, 4 h) was used as a positive control to induce DNA laddering. **r** Percentage of apoptosis (stained with $Annv^+/PI^-$) or necrotosis (stained with $Annv^-/PI^+$ and $Annv^+/PI^+$) was indicated. Cells were treated with CCCP/$FeSO_4$ for the indicated time. Annv: Annexin V; PI: propidium iodide. **s** CCCP/$FeSO_4$ showed no

effect on nuclear fragmentation of cells. The cells were treated with CCCP/$FeSO_4$ for 24 h, the nucleus was stained with Hoechest33342. STS (2 μM, 24 h) was used as a positive control to indicate the nuclear fragmentation. DIC: differential interference contrast image. **t** CCCP/$FeSO_4$ showed a weaker effect on PARP cleavage as compared to STS induction. The cells were treated with CCCP/$FeSO_4$ for 24 h. **u** Prolonged treatment with CCCP/$FeSO_4$ could induce apoptosis in cells. The GSDME-knockdown A375 cells, or ME4405 and Mel-11 cells (non-expression of GSDME) were treated with CCCP/$FeSO_4$ for 24 or 36 h, the nuclear fragmentation stained with Hoechest33342 was indicated. Actin was used to determine the amount of loading RNA. Tubulin was used to determine the amount of loading proteins. All data are presented as the mean ± SEM of three independent experiments. ns, not significant.
