# Supplementary material for: Tom20 senses iron-activated ROS signaling to promote melanoma cell pyroptosis
Source: Cell Res. 2018 Oct 4;28(12):1171–85. doi: 10.1038/s41422-018-0090-y (PMC6274649; doi:10.1038/s41422-018-0090-y)
Supplement: Supplementary file 2 — Supplementary information, Figure S2 [file 41422_2018_90_MOESM2_ESM.pdf]

# Supplementary Figure 2

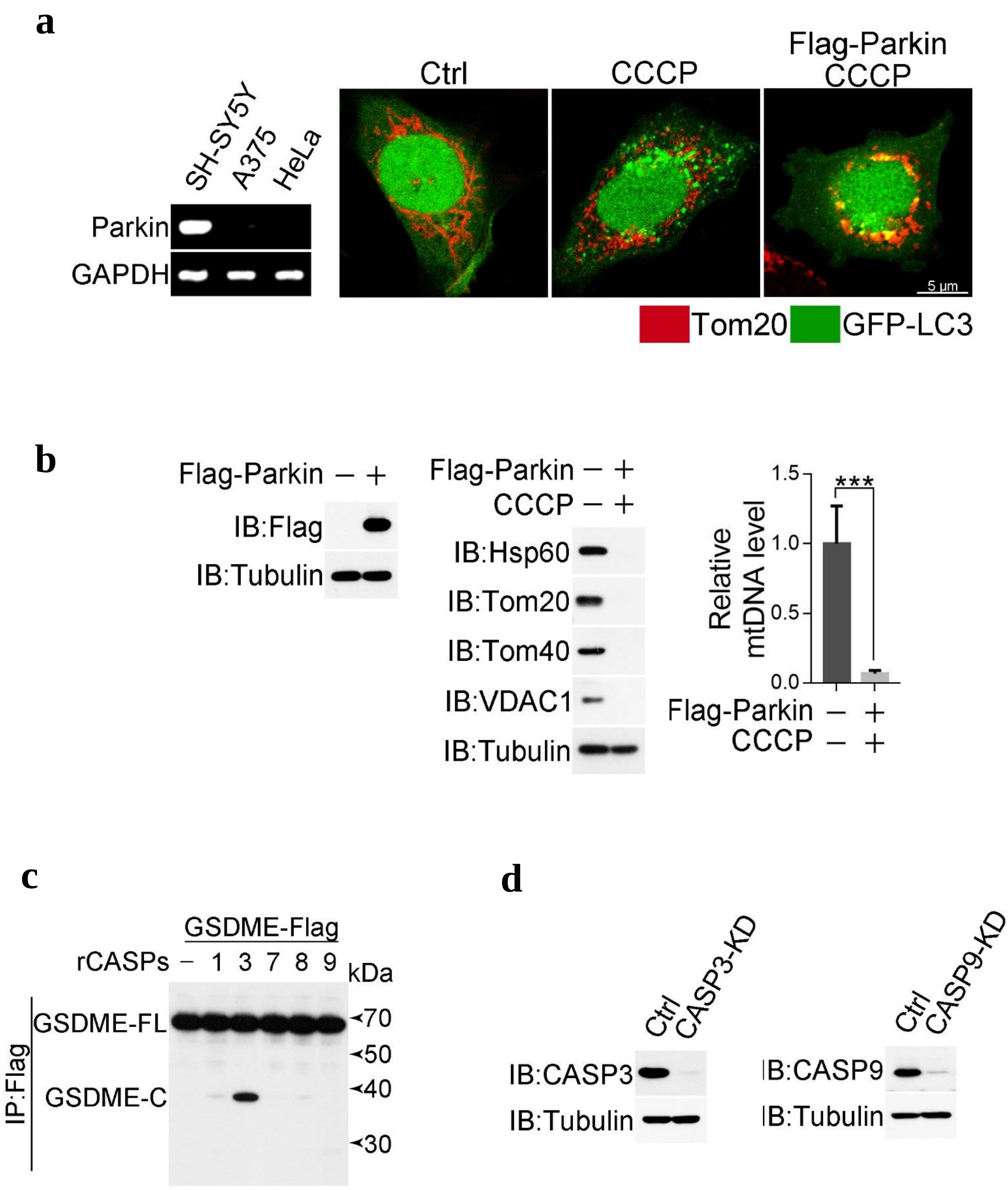

**e**

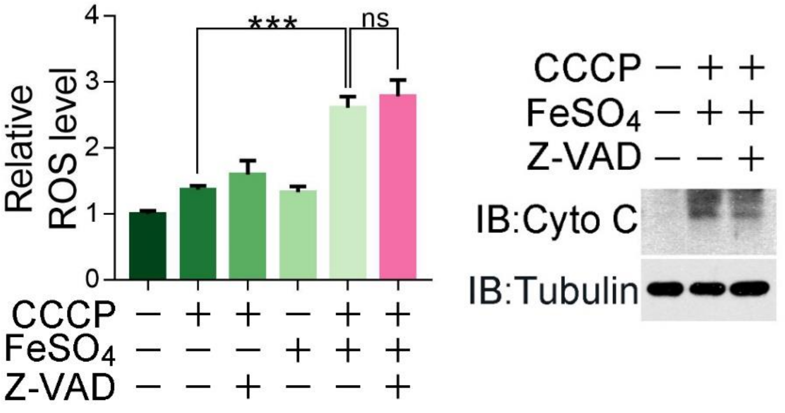

Relative ROS level
***
ns
CCCP − + + − + +
FeSO4 − − − + + +
Z-VAD − − + − − +
CCCP − + +
FeSO4 − + +
Z-VAD − − +
IB:Cyto C
IB:Tubulin


**f**

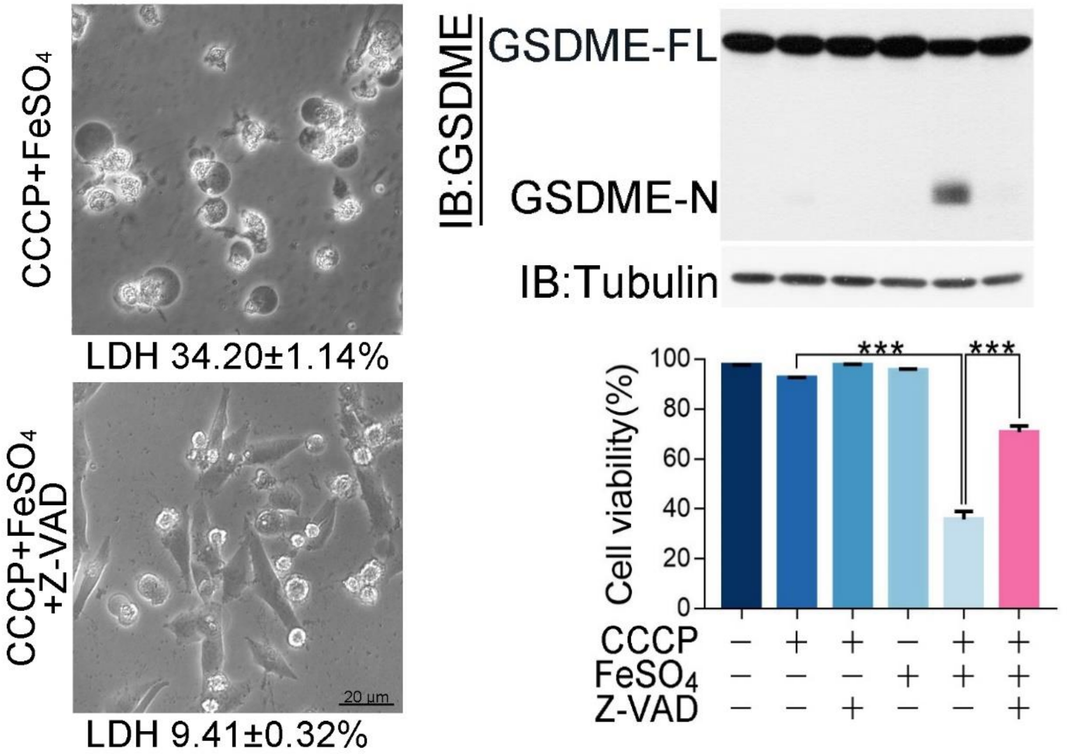

CCCP+FeSO4
LDH 34.20±1.14%
CCCP+FeSO4 +Z-VAD
20 µm
LDH 9.41±0.32%
IB:GSDME
GSDME-FL
GSDME-N
IB:Tubulin
Cell viability(%)
***
***
CCCP − + + − + +
FeSO4 − − − + + +
Z-VAD − − + − − +

**Figure S2.** Melanoma A375 cells were pretreated with or without Z-VAD (20 μM) as indicated for 2 h, followed by CCCP (20 μM), $FeSO_4$ (100 μM), or CCCP/$FeSO_4$ treatment for 6 h to detect ROS generation, cytochrome c, and caspase-3 and -9 or 24 h to assess the pyroptotic features (including morphology, GSDME cleavage, and LDH release), unless specially defined. **a** Left, expression levels of the Parkin gene were detected in A375, SH-SY5Y (positive control), and HeLa (negative control) cells. Right, CCCP induced mitophagy in Parkin-expressing cells as revealed by the co-localization of mitochondria with LC3. **b** Overexpression of Parkin (left) facilitates the mitochondrial clearance, as revealed by the expression levels of various mitochondrial proteins (middle) and the level of mitochondrial DNA (mtDNA, right). Cells transfected with Parkin were treated with CCCP (15 μM) for 36 h. **c** The *in vitro* cleavage of immunoprecipitated GSDME by different recombinant caspases (rCASPs). **d** Efficiencies of the caspase-3 or caspase-9 knockdown in the cells as detected by western blotting. **e** Z-VAD had no effect on CCCP/$FeSO_4$-activated ROS (left) and cytochrome c release as detected in the cytosol fraction (right). **f** Z-VAD could attenuate CCCP/$FeSO_4$-induced pyroptosis, including cell morphology, GSDME cleavage, and cell death. Tubulin was used to determine the amount of loading proteins. GAPDH was used to determine the amount of loading mRNA. All data are presented as the mean ± SEM of three independent experiments. ***$P < 0.001$, ns, not significant.
