# Supplementary material for: Tom20 senses iron-activated ROS signaling to promote melanoma cell pyroptosis
Source: Cell Res. 2018 Oct 4;28(12):1171–85. doi: 10.1038/s41422-018-0090-y (PMC6274649; doi:10.1038/s41422-018-0090-y)
Supplement: Supplementary file 3 — Supplementary information, Figure S3 [file 41422_2018_90_MOESM3_ESM.pdf]

# Supplementary Figure 3

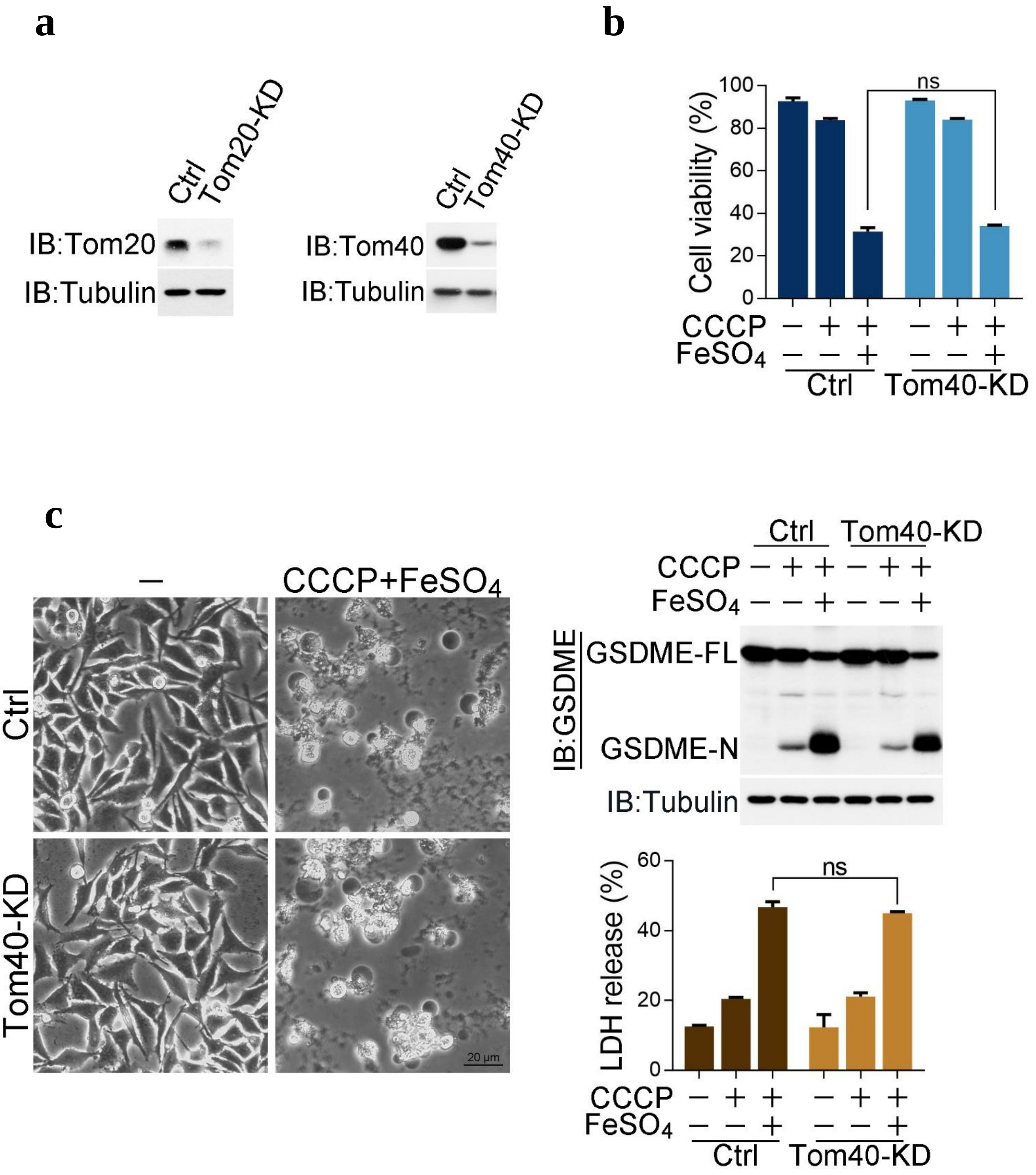

**d**

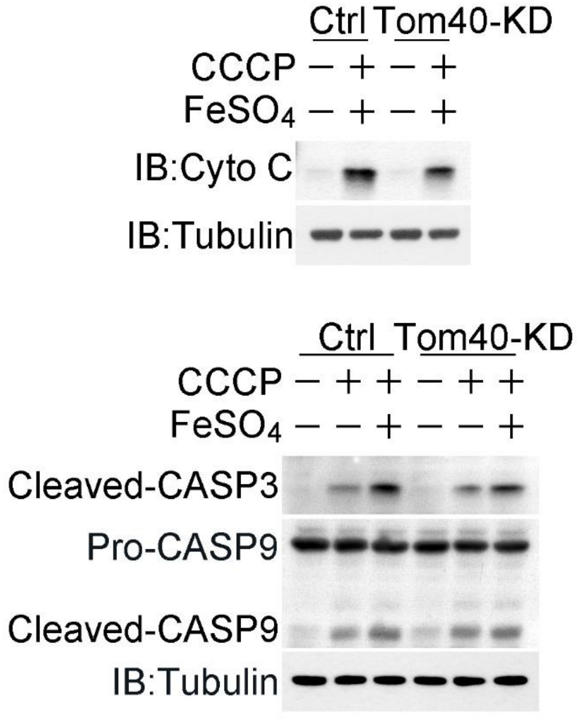
Ctrl
Tom40-KD
CCCP − + − +
FeSO4 − + − +
IB:Cyto C
IB:Tubulin
Ctrl
Tom40-KD
CCCP − + + − + +
FeSO4 − − + − − +
Cleaved-CASP3
Pro-CASP9
Cleaved-CASP9
IB:Tubulin

**g**

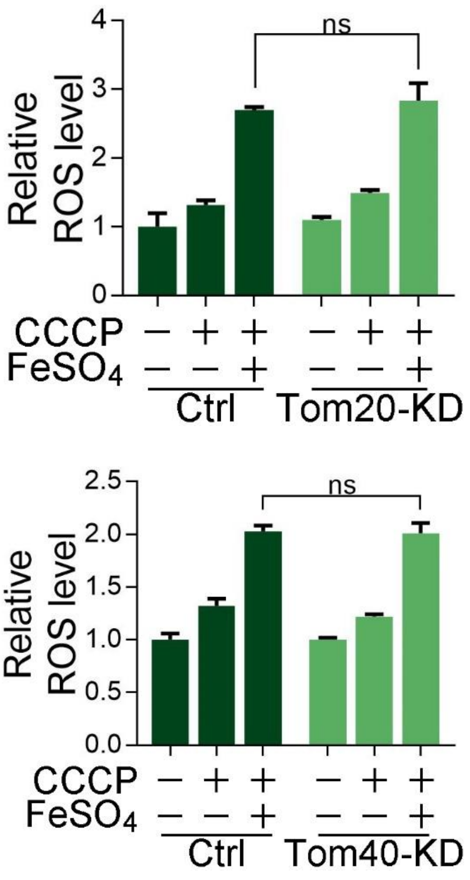
Relative ROS level
ns
0
1
2
3
4
CCCP − + + − + +
FeSO4 − − + − − +
Ctrl
Tom20-KD
Relative ROS level
ns
0.0
0.5
1.0
1.5
2.0
2.5
CCCP − + + − + +
FeSO4 − − + − − +
Ctrl
Tom40-KD

**e**

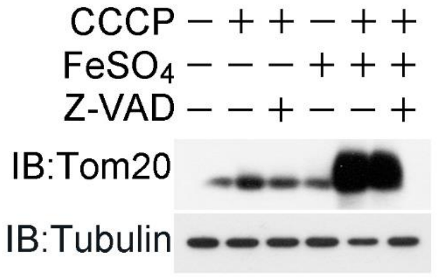
CCCP − + + − + +
FeSO4 − − − + + +
Z-VAD − − + − − +
IB:Tom20
IB:Tubulin

**f**

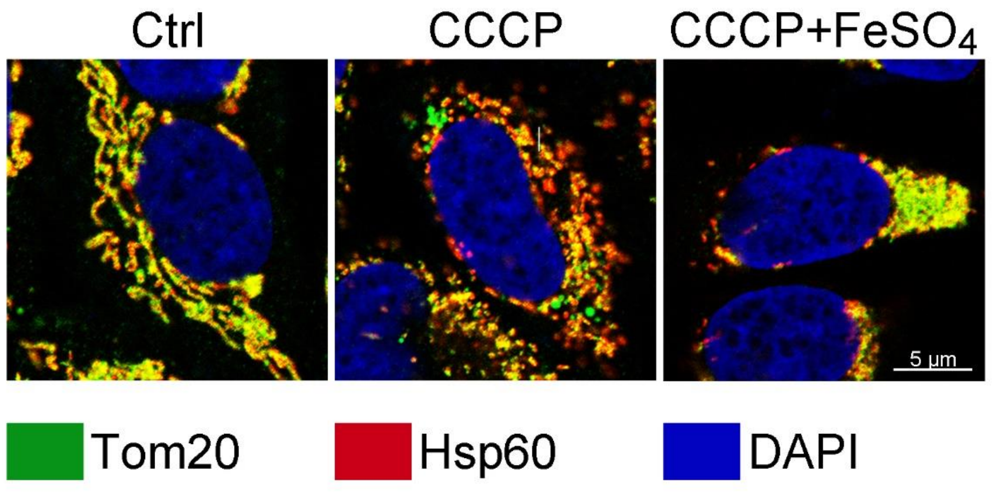
Ctrl
CCCP
CCCP+FeSO4
5 μm
Tom20
Hsp60
DAPI

**Figure S3.** Melanoma A375 cells were treated with CCCP (20 μM), $FeSO_4$ (100 μM), or CCCP/$FeSO_4$ for 6 h to detect ROS generation, 12 h to detect mitochondrial aggregation, or 24 h to assess the pyroptotic features (including morphology, GSDME cleavage, and LDH release) and cell viability, unless specially defined. **a** Efficiencies of Tom20 or Tom40 knockdown in the cells as detected by western blotting. **b** Tom40 knockdown had no effect on CCCP/$FeSO_4$-induced cell death. **c** Tom40 knockdown had no effect on CCCP/$FeSO_4$-induced pyroptosis, including cell morphology, GSDME cleavage, and LDH release. **d** Tom40 knockdown had no effects on cytochrome c release and cleavage of caspase-3 and -9. The cytosolic fraction was prepared for the detection of cytochrome c. **e** Co-treatment of Z-VAD could not impair CCCP/$FeSO_4$-induced Tom20 accumulation. **f** CCCP/$FeSO_4$ treatment did not influence the mitochondrial location of Tom20. Hsp60 indicated the mitochondria, and DAPI displayed the nuclei. **g** Neither the Tom40 nor Tom20 knockdown had an effect on CCCP/$FeSO_4$-induced ROS generation. Tubulin was used to determine the amount of loading proteins. All data are presented as the mean ± SEM of three independent experiments. ns, not significant.
