# Supplementary material for: Tom20 senses iron-activated ROS signaling to promote melanoma cell pyroptosis
Source: Cell Res. 2018 Oct 4;28(12):1171–85. doi: 10.1038/s41422-018-0090-y (PMC6274649; doi:10.1038/s41422-018-0090-y)
Supplement: Supplementary file 4 — Supplementary information, Figure S4 [file 41422_2018_90_MOESM4_ESM.pdf]

# Supplementary Figure 4

**a**

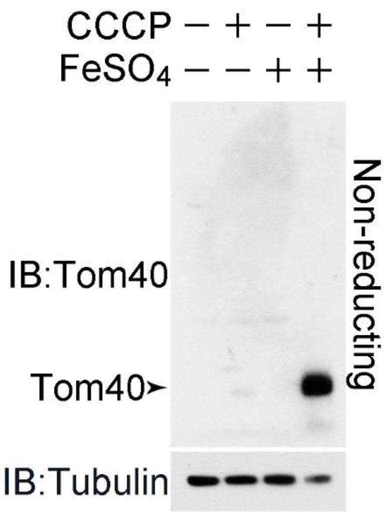


**c**

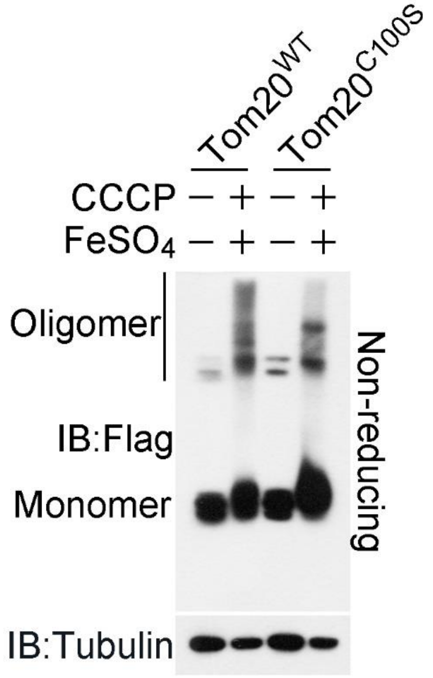


**b**

Tom20 C13/C21

| | |
|---|---|
| Human(8-25) | IAAGVCGALFIGYCIYFD |
| Chimpanzee | IAAGVCGALFIGYCIYFD |
| Mouse | IAAGVCGALFIGYCIYFD |
| Rat | IAAGVCGALFIGYCIYFD |
| Horse | IAAGVCGALFIGYCIYFD |
| Dog | IAAGVCGALFIGYCIYFD |
| Cow | IAAGVCGALFIGYCIYFD |
| Frog | IAAGVCGALLLGYCIYFD |
| Zebrafish | IAAGLGAALFVGYCIYFD |

Tom20 C100

| | |
|---|---|
| Human(96-104) | AIAVCGQPQ |
| Chimpanzee | AIAVCGQPQ |
| Mouse | AIAVCGQPQ |
| Rat | AIAVCGQPQ |
| Horse | AIAVCGQPQ |
| Dog | AIAVCGQPQ |
| Cow | AIAVCGQPQ |
| Frog | AIA ICGQPQ |
| Zebrafish | AIAVCGQPQ |

**d**

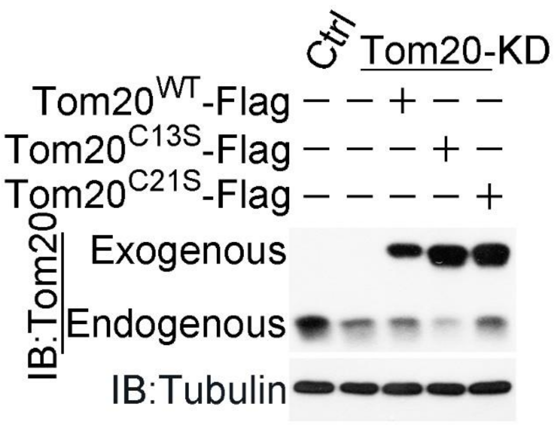

**Figure S4.** Melanoma A375 cells were treated with CCCP (20 μM), $FeSO_4$ (100 μM), or CCCP/$FeSO_4$ for 24 h in each experiment, unless specially defined. To detect the effects of the Tom20 point mutants $Tom20^{C13S}$, $Tom20^{C21S}$, and $Tom20^{C100S}$, Tom20 was first knocked down in the cells, and then, $Tom20^{WT}$ or its point mutants $Tom20^{C13S}$, $Tom20^{C21S}$, and $Tom20^{C100S}$ were separately transfected into the cells. **a** CCCP/$FeSO_4$ did not induce the high-molecular weight complex of Tom40 in the relative position. **b** In the Tom20 molecule, three Cys residues conserved in several species are indicated. **c** Mutation of Cys100 in the Tom20 molecule had no effect on CCCP/$FeSO_4$-induced Tom20 oxidation. **d** Indications of the $Tom20^{WT}$, $Tom20^{C13S}$ and $Tom20^{C21S}$ expression levels in Tom20-knockdown cells. Tubulin was used to determine the amount of loading proteins.
