# Supplementary material for: Tom20 senses iron-activated ROS signaling to promote melanoma cell pyroptosis
Source: Cell Res. 2018 Oct 4;28(12):1171–85. doi: 10.1038/s41422-018-0090-y (PMC6274649; doi:10.1038/s41422-018-0090-y)
Supplement: Supplementary file 5 — Supplementary information, Figure S5 [file 41422_2018_90_MOESM5_ESM.pdf]

Supplementary Figure 5

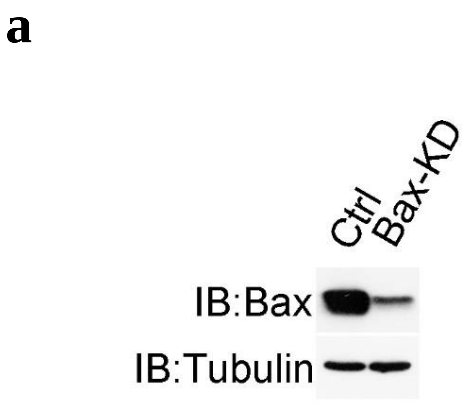

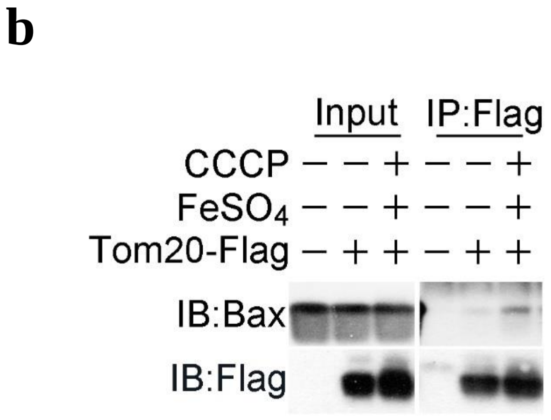

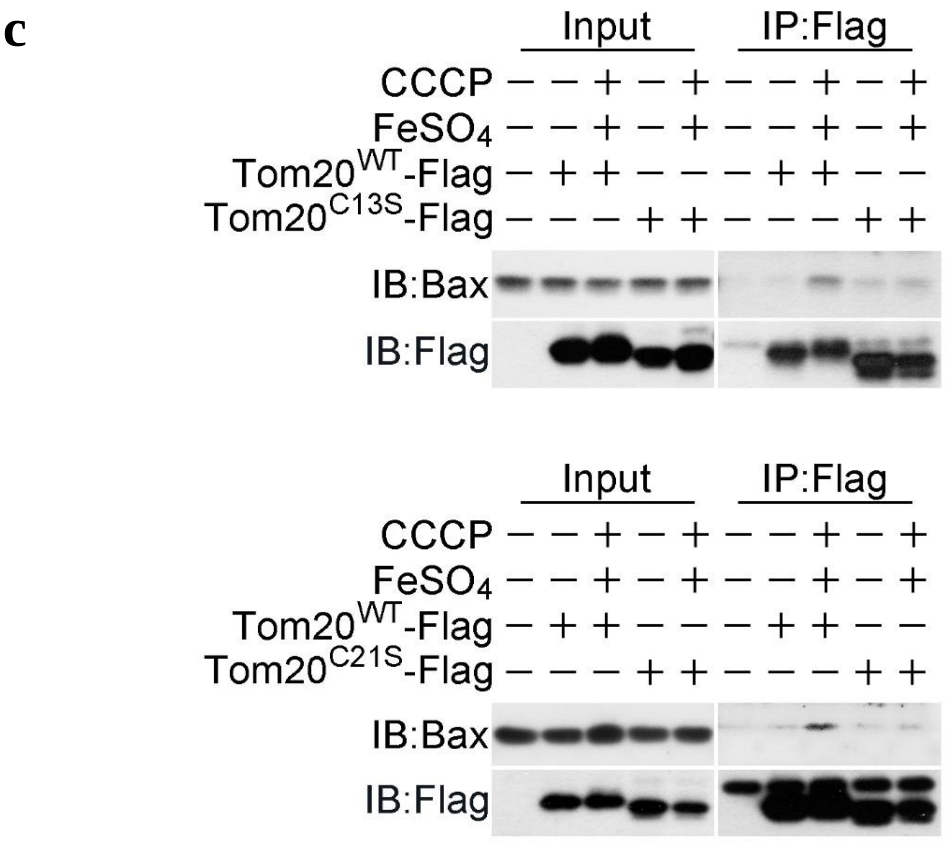

d

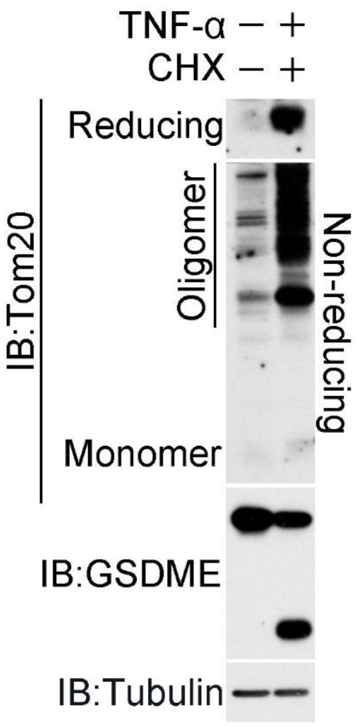
TNF-α − +
CHX − +
Reducing
Oligomer
Non-reducing
IB:Tom20
Monomer
IB:GSDME
IB:Tubulin

e

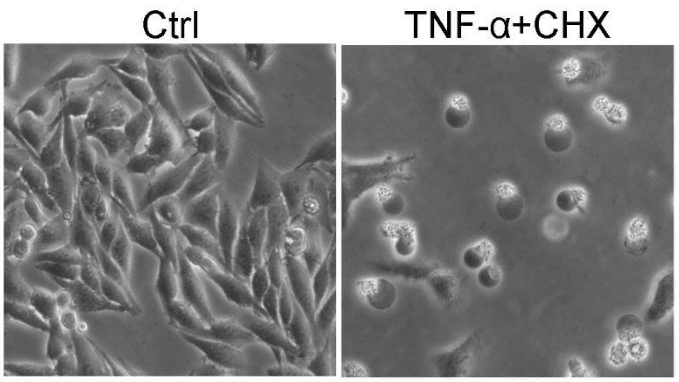
Ctrl
TNF-α+CHX

Figure S5. Melanoma A375 cells were treated with CCCP (20 μM), $FeSO_4$ (100 μM), or CCCP/$FeSO_4$ for 6 h in each experiment, unless specially defined. To detect the effects of the Tom20 point mutants $Tom20^{C13S}$, $Tom20^{C21S}$, and $Tom20^{C100S}$, Tom20 was first knocked down in the cells, and then, $Tom20^{WT}$ or its point mutants $Tom20^{C13S}$ and $Tom20^{C21S}$ were separately transfected into cells. **a** Efficiencies of Bax knockdown in the cells as detected by western blotting. **b** CCCP/$FeSO_4$ enhanced Tom20 interaction with Bax. Tom20 was transfected into the cells, and a co-IP assay was performed. **c** Mutation of either Cys13 or Cys21 in the Tom20 molecule attenuated the CCCP/$FeSO_4$-enhanced Tom20 interaction with Bax. Cells were transfected with Bax and $Tom20^{C13S}$ or $Tom20^{C21S}$ as indicated, and the Co-IP assays were performed. To compare the binding affinity of Bax with Tom20 or its mutants in co-IP assays, Tom20 and its mutants' expressions should be the same. To this end, the amounts of Tom20, $Tom20^{C13S}$ and $Tom20^{C21S}$ were adjusted to ensure the comparable expression levels in the cell lysates. **d** TNF-α/CHX induced Tom20 oxidation. The cells were treated with TNF-α (20 ng/mL) plus CHX (cycloheximide, 10 μg/mL) for 24 h, western blotting was performed under reducing and non-reducing conditions to determine the Tom20 oxidation status. The cleavage of GSDME was also detected. **e** TNF-α/CHX induced pyroptosis. The cells were treated with TNF-α (20 ng/mL) plus CHX (10 μg/mL) for 24 h, the pyroptotic cells were indicated. Tubulin was used to determine the amount of loading proteins.
