# Supplementary material for: Tom20 senses iron-activated ROS signaling to promote melanoma cell pyroptosis
Source: Cell Res. 2018 Oct 4;28(12):1171–85. doi: 10.1038/s41422-018-0090-y (PMC6274649; doi:10.1038/s41422-018-0090-y)
Supplement: Supplementary file 6 — Supplementary information, Figure S6 [file 41422_2018_90_MOESM6_ESM.pdf]

Supplementary Figure 6

a

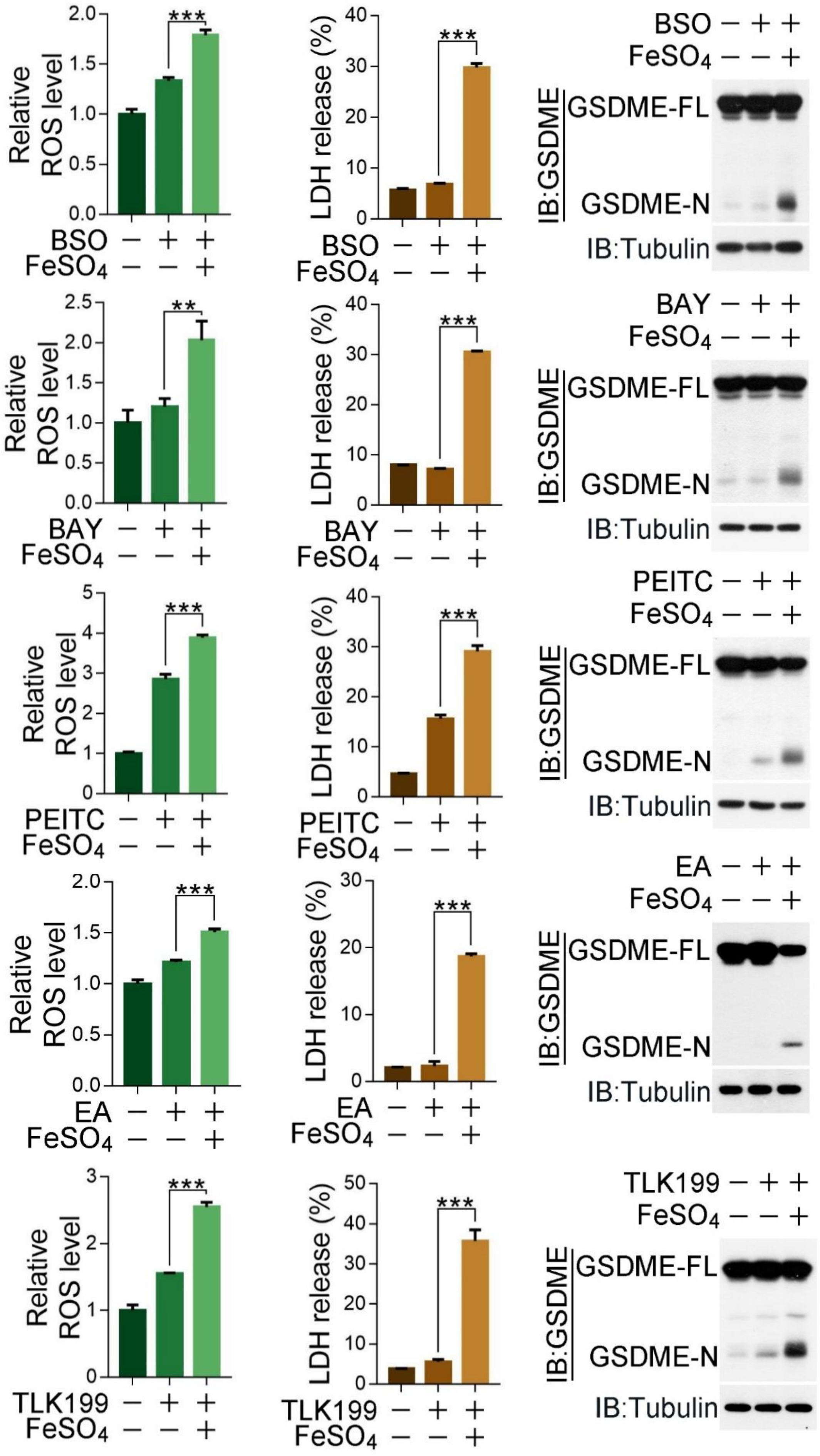

b

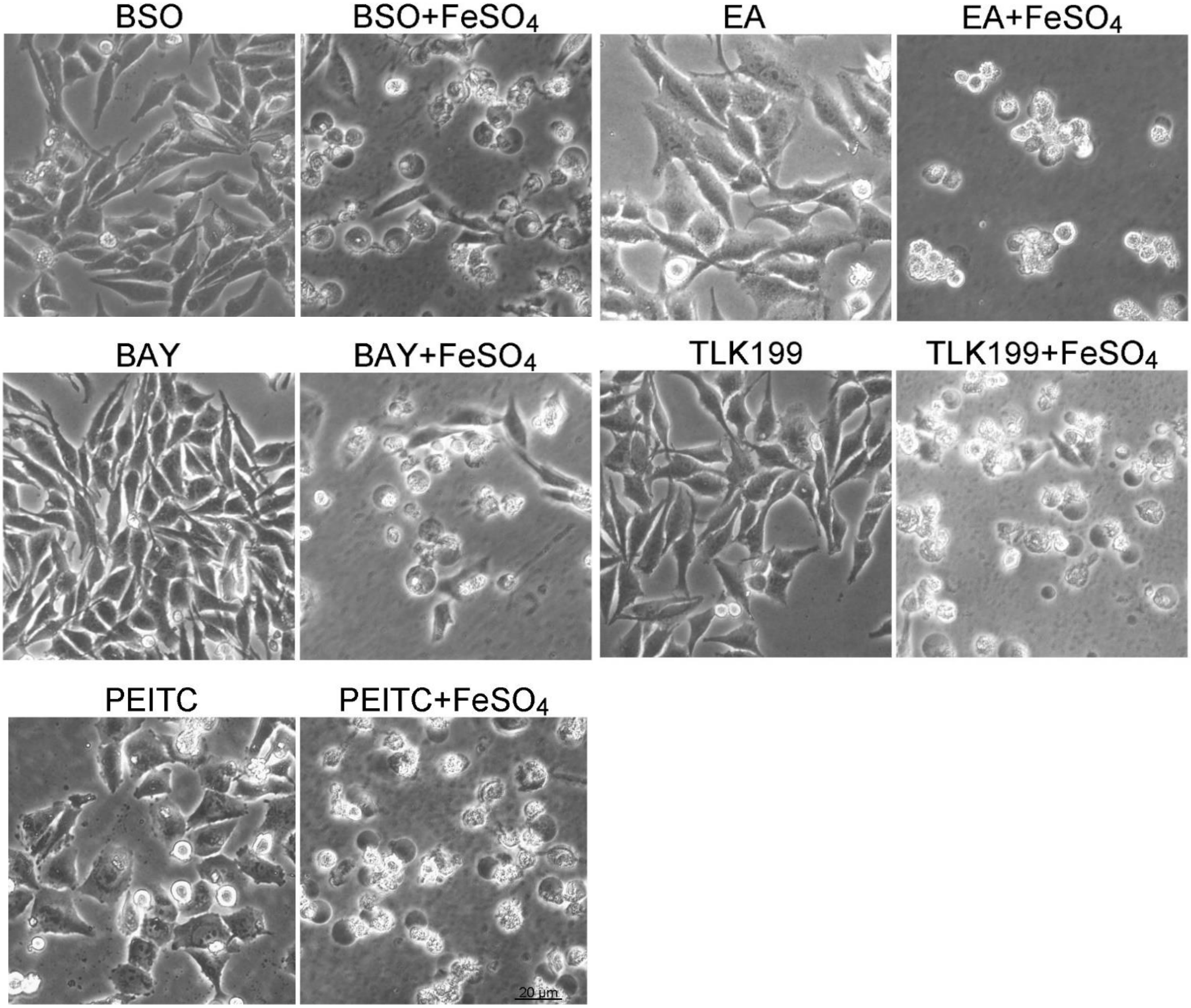
BSO
BSO+$FeSO_4$
EA
EA+$FeSO_4$
BAY
BAY+$FeSO_4$
TLK199
TLK199+$FeSO_4$
PEITC
PEITC+$FeSO_4$
20 μm

c

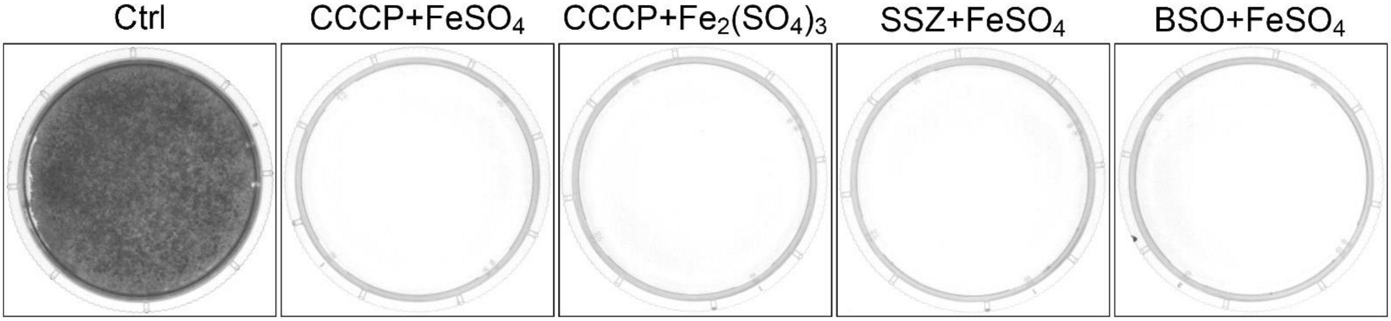
Ctrl
CCCP+$FeSO_4$
CCCP+$Fe_2(SO_4)_3$
SSZ+$FeSO_4$
BSO+$FeSO_4$

d

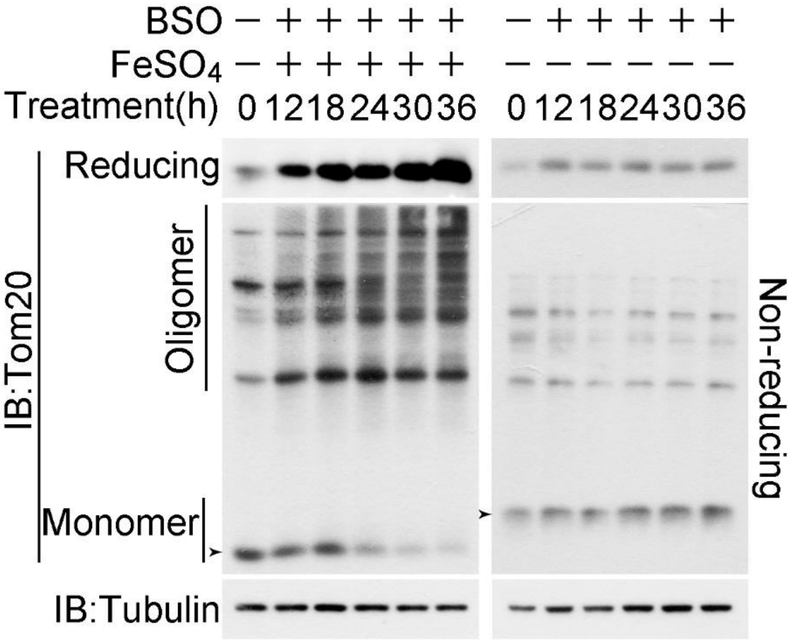
BSO − + + + + + − + + + + +
FeSO4 − + + + + + − − − − − −
Treatment(h) 0 12 18 24 30 36 0 12 18 24 30 36
Reducing
Oligomer
IB:Tom20
Monomer
Non-reducing
IB:Tubulin

e

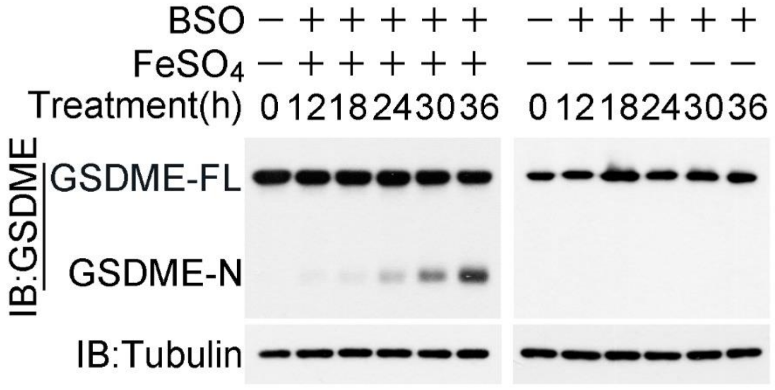
BSO − + + + + + − + + + + +
FeSO4 − + + + + + − − − − − −
Treatment(h) 0 12 18 24 30 36 0 12 18 24 30 36
IB:GSDME
GSDME-FL
GSDME-N
IB:Tubulin

f

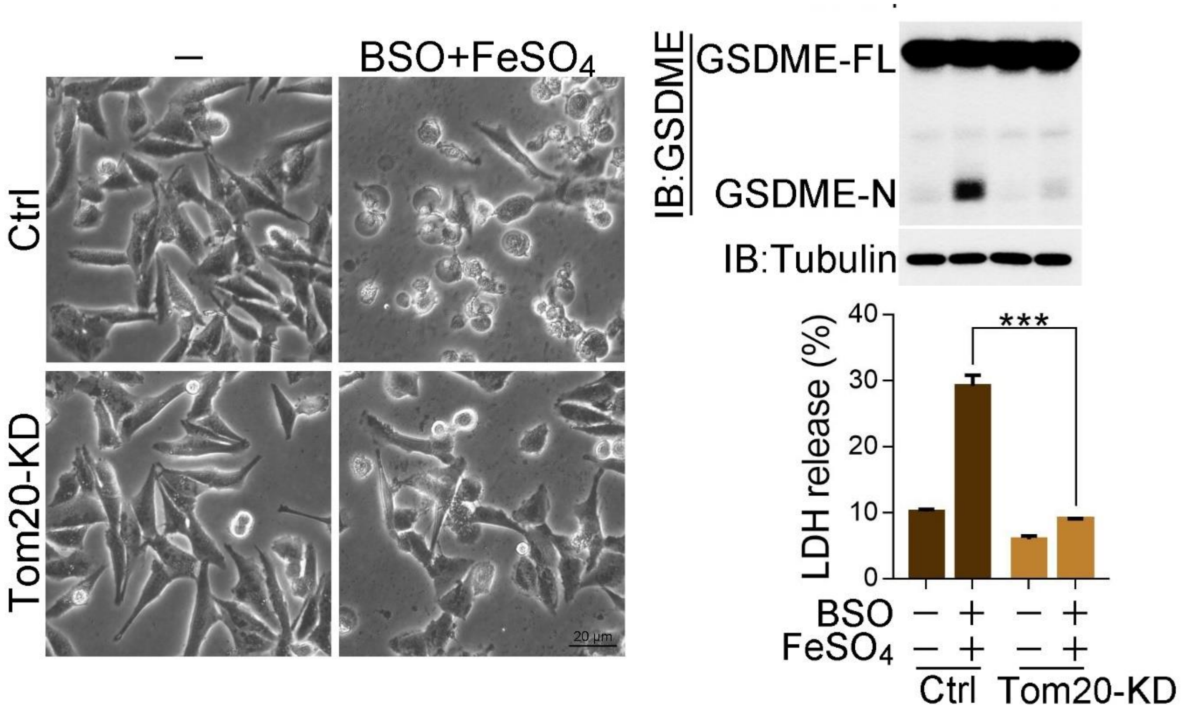
−
BSO+FeSO4
Ctrl
Tom20-KD
20 μm
IB:GSDME
GSDME-FL
GSDME-N
IB:Tubulin
LDH release (%)
40
30
20
10
0
***
BSO − + − +
FeSO4 − + − +
Ctrl
Tom20-KD

g

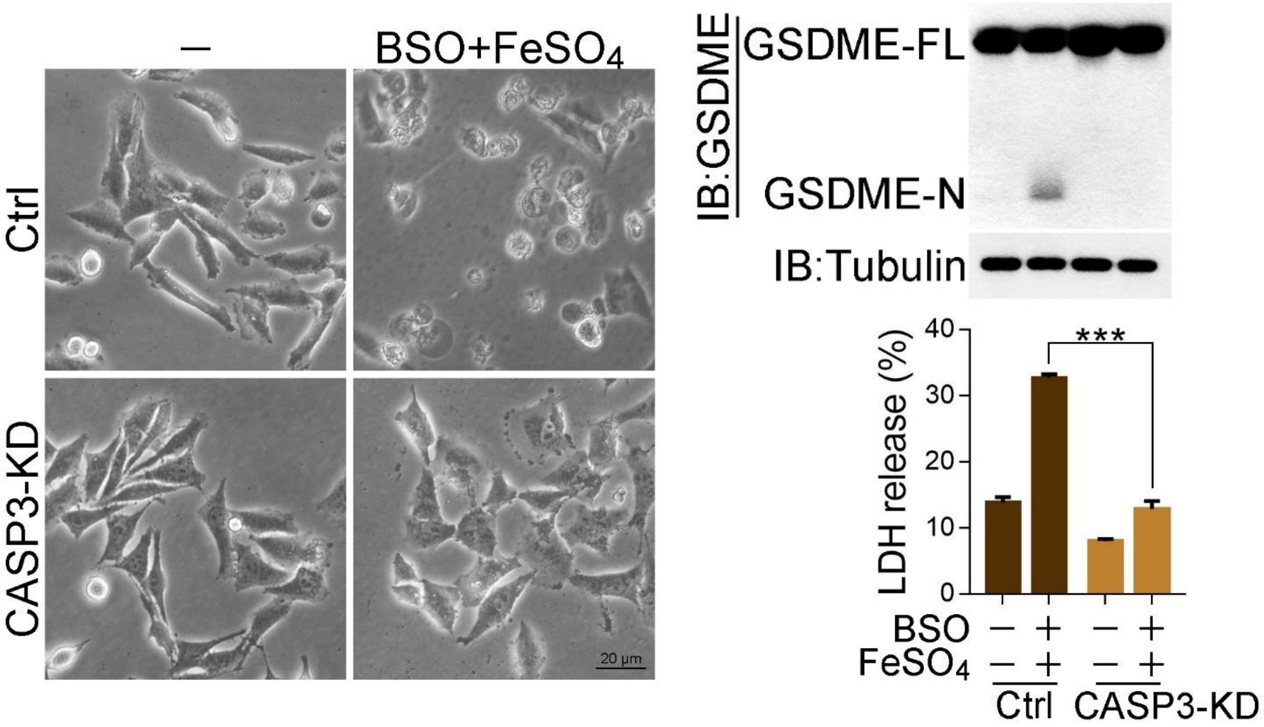

—
BSO+FeSO4
Ctrl
CASP3-KD
20 μm
IB:GSDME
GSDME-FL
GSDME-N
IB:Tubulin
LDH release (%)
40
30
20
10
0
***
BSO − + − +
FeSO4 − + − +
Ctrl
CASP3-KD

h

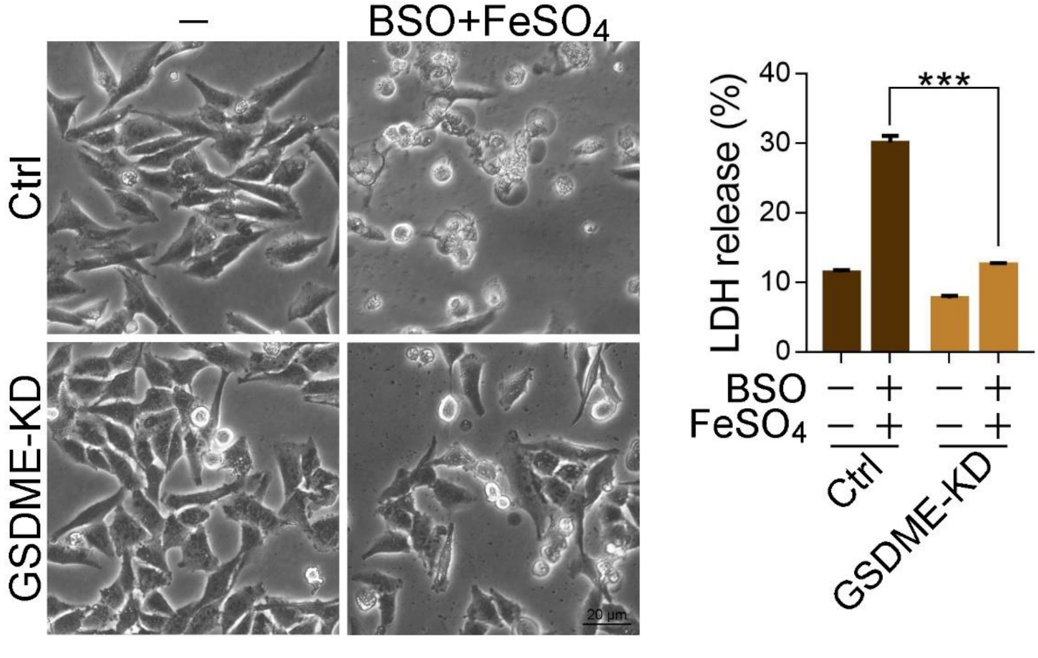

—
BSO+FeSO4
Ctrl
GSDME-KD
20 μm
LDH release (%)
40
30
20
10
0
***
BSO − + − +
FeSO4 − + − +
Ctrl
GSDME-KD

i

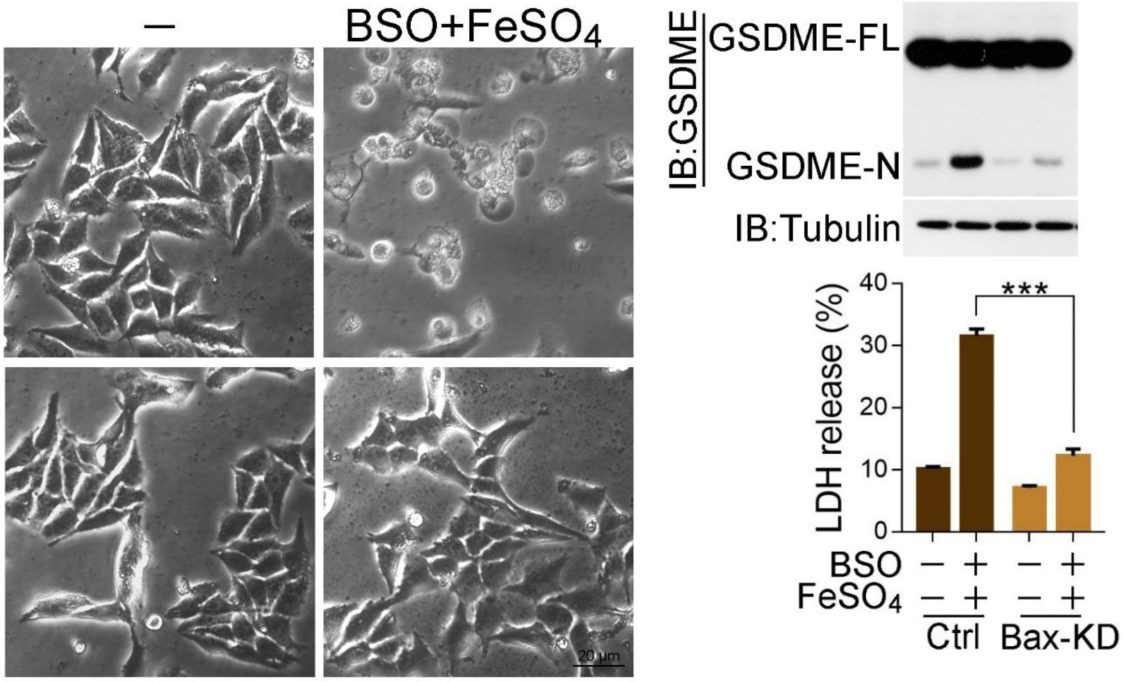

—
BSO+FeSO4
Ctrl
Bax-KD
20 μm
IB:GSDME
GSDME-FL
GSDME-N
IB:Tubulin
LDH release (%)
0
10
20
30
40
***
BSO − + − +
FeSO4 − + − +
Ctrl
Bax-KD

j

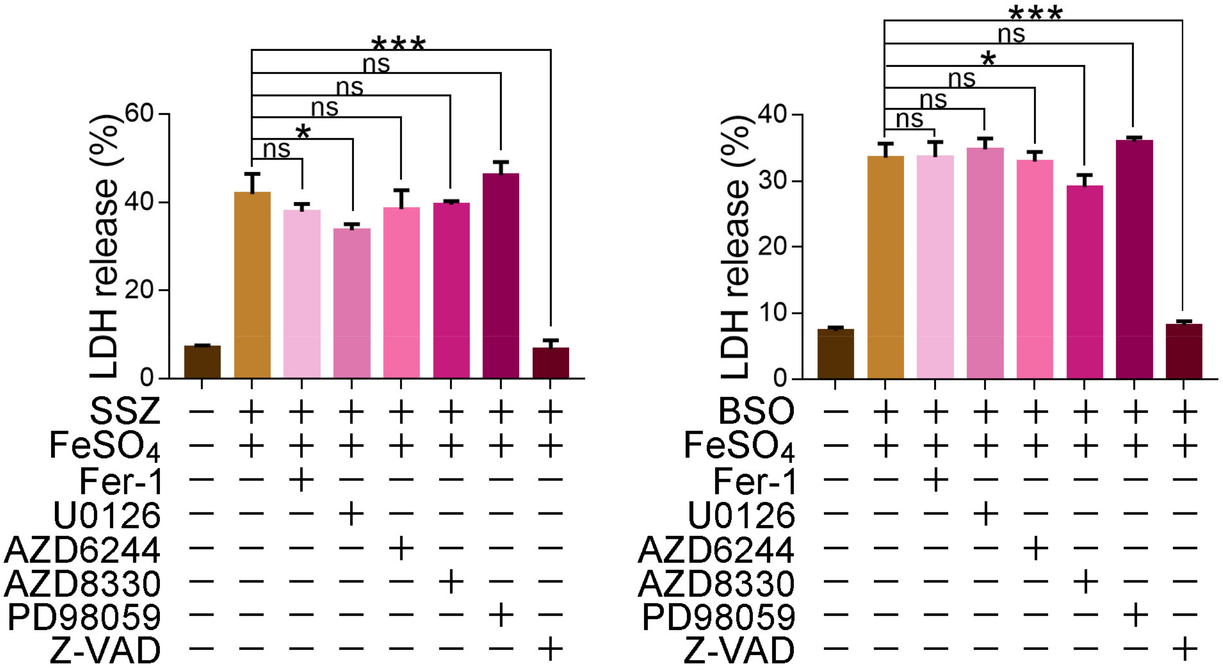

LDH release (%)
0
20
40
60
***
ns
ns
ns
*
ns
SSZ − + + + + + + +
FeSO4 − + + + + + + +
Fer-1 − − + − − − − −
U0126 − − − + − − − −
AZD6244 − − − − + − − −
AZD8330 − − − − − + − −
PD98059 − − − − − − + −
Z-VAD − − − − − − − +
LDH release (%)
0
10
20
30
40
***
ns
*
ns
ns
ns
BSO − + + + + + + +
FeSO4 − + + + + + + +
Fer-1 − − + − − − − −
U0126 − − − + − − − −
AZD6244 − − − − + − − −
AZD8330 − − − − − + − −
PD98059 − − − − − − + −
Z-VAD − − − − − − − +

Figure S6. Melanoma A375 cells were treated with BSO (DL-buthionine-sulfoximine, 250 μM) plus $FeSO_4$ (100 μM) for 6 h to detect the ROS level or 24 h to detect GSDME cleavage, LDH release, and the pyroptotic morphology in each experiment, unless specially defined. **a** Different drugs with or without $FeSO_4$ addition showed significant effects on ROS elevation, LDH release, and GSDME cleavage as indicated. Cells were treated with various drugs (including BSO, PEITC (phenylethyl isothiocyanate, 5 μM), EA (etacrynic acid, 50 μM), and TLK199 (ezatiostat, 20 μM)) plus $FeSO_4$ for 24 h, and treated with BAY (BAY 87-2243, 2.5 nM) plus $FeSO_4$ for 36 h. **b** Morphologies of A375 cells following treatment with various drugs with or without $FeSO_4$ addition as indicated. **c** CCCP/SSZ or CCCP/BSO could inhibit the clonogenic survival of A375 cells as efficiently as CCCP/$FeSO_4$ or CCCP/$Fe_2(SO_4)_3$ in the long-term colony formation assays. The cells were treated with different reagents as indicated for 10 days, and the colony formations were indicated. **d, e** Extensive treatment of $FeSO_4$ together with BSO as indicated induced Tom20 oxidation, accumulation **(d)** and GSDME cleavage **(e)**. **f-i** Separately knocking down Tom20 (F), caspase-3 (G), GSDME (H), or Bax (I) blocked BSO/$FeSO_4$-induced pyroptosis, GSDME cleavage, and LDH release as indicated. **j** Inhibitors of ferroptosis (ferrostatin-1 (0.5 μM), U0126 (20 μM), AZD6244 (5 μM), AZD8330 (2.5 μM) and PD98059 (5 μM)) could not impair the CCCP/$FeSO_4$-induced LDH release. Cells were pretreated with or without different inhibitors as indicated for 2 h, followed by treatment of SSZ/$FeSO_4$ or BSO/$FeSO_4$ for 24 h to detect the levels of LDH. Z-VAD was used as a positive control. Fer-1: ferrostatin-1. Tubulin was used to

determine the amount of loading proteins. All data are presented as the mean ± SEM of three independent experiments. **$P < 0.01$, ***$P < 0.001$.
