# Supplementary material for: Tom20 senses iron-activated ROS signaling to promote melanoma cell pyroptosis
Source: Cell Res. 2018 Oct 4;28(12):1171–85. doi: 10.1038/s41422-018-0090-y (PMC6274649; doi:10.1038/s41422-018-0090-y)
Supplement: Supplementary file 7 — Supplementary information, Figure S7 [file 41422_2018_90_MOESM7_ESM.pdf]

Supplementary Figure 7

a

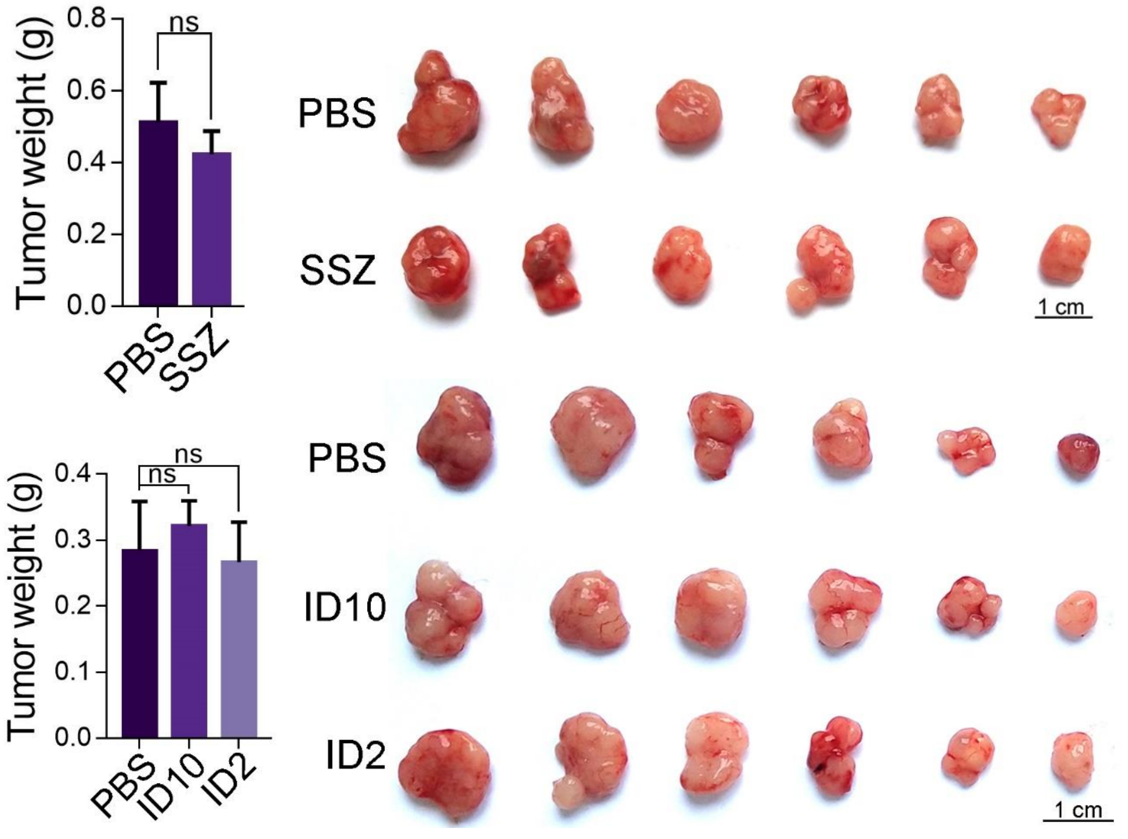

b

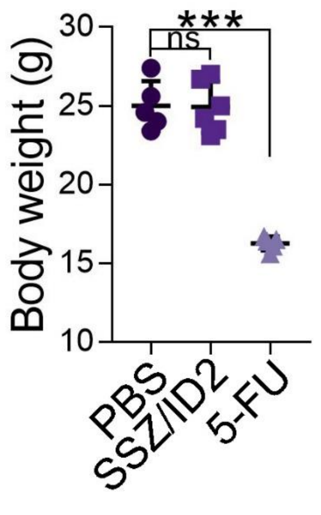

c

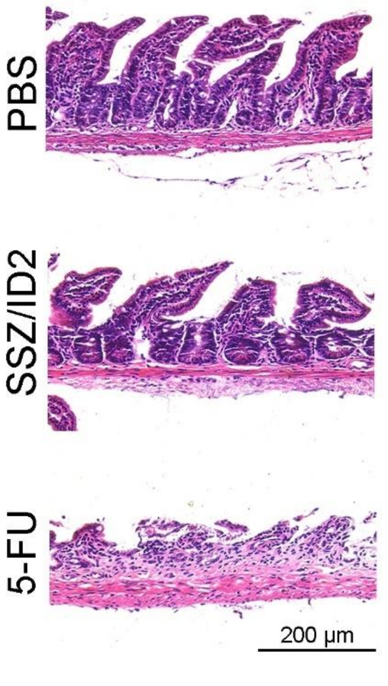

d

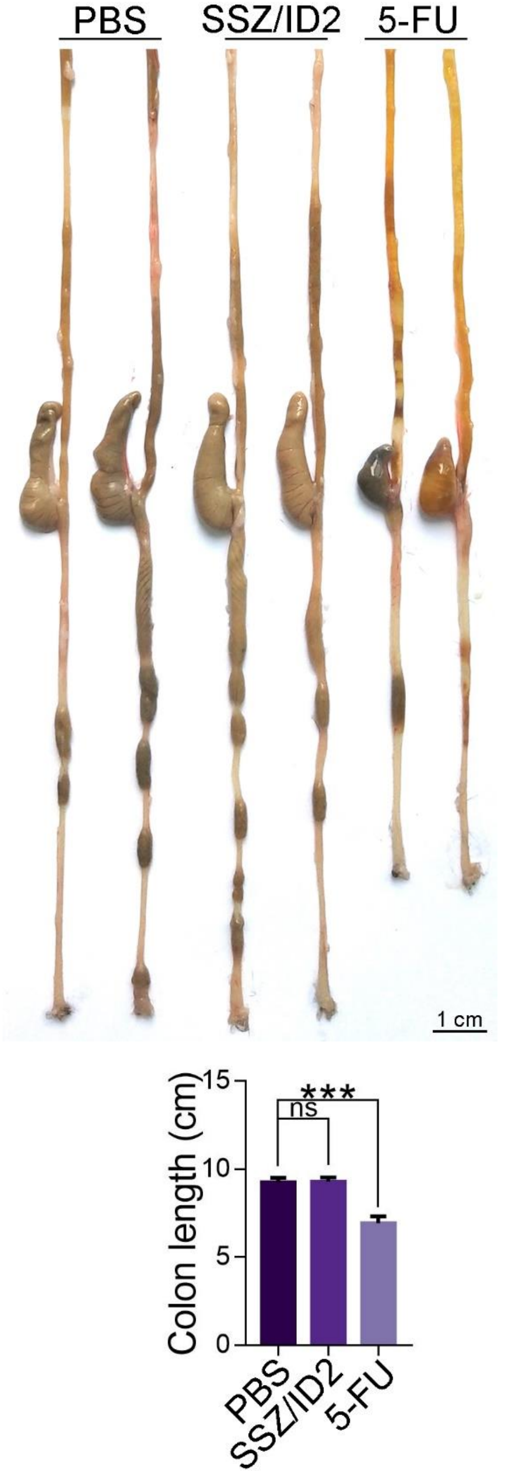
PBS
SSZ/ID2
5-FU
1 cm
Colon length (cm)
15
10
5
0
ns
***
PBS
SSZ/ID2
5-FU

e

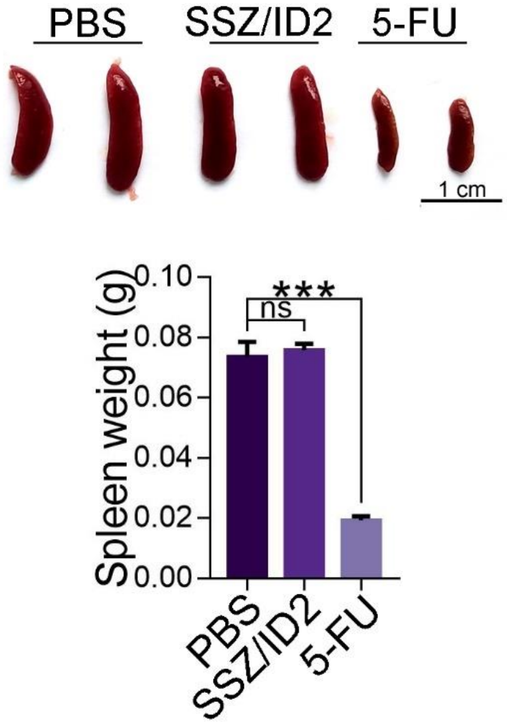
PBS
SSZ/ID2
5-FU
1 cm
Spleen weight (g)
0.10
0.08
0.06
0.04
0.02
0.00
ns
***
PBS
SSZ/ID2
5-FU

Figure S7. Iron showed no side effect on mice. **a** SSZ (sulfasalazine) or ID (iron dextran solution) administration had no effect on A375 cell-driven xenograft tumors. Melanoma A375 cells were injected subcutaneously into the posterior flanks of nude mice (n = 6). After four days, SSZ (50 mg/kg) or iron dextran (10 mg/kg) was intraperitoneally administered to the mice every other day for 2 weeks. The tumor volume was recorded, and images of xenograft tumors in nude mice were shown. **b-e** Administration of iron dextran solution with SSZ showed no side effects on mice. Mice were intraperitoneally injected with SSZ (50 mg/kg) and ID (2 mg/kg) or PBS control as indicated. Various side effects on mice, including mouse weight loss **(b)**, crypt disruption in the small intestine **(c)**, colon shortening (scale bar, 1 cm) **(d)**, and spleen atrophy **(e)**, were analyzed. Fluorouracil (5-FU) was used as a positive control.
