# Supplementary material for: Tom20 senses iron-activated ROS signaling to promote melanoma cell pyroptosis
Source: Cell Res. 2018 Oct 4;28(12):1171–85. doi: 10.1038/s41422-018-0090-y (PMC6274649; doi:10.1038/s41422-018-0090-y)
Supplement: Supplementary file 8 — Supplementary movie S1 legend [file 41422_2018_90_MOESM8_ESM.docx]

**Supplementary movie**. Pyroptosis induced by CCCP/FeSO_4_ in A375 cells. For time-lapse microscopy, A375 cells were grown on 35-mm glass bottom dish (Nest). DIC images were acquired with Zeiss LSM 780 after CCCP/FeSO_4_ treatment.
